# Stretch versus shortening contractions subsequently decrease versus increase neural drive to the human tibialis anterior

**DOI:** 10.1113/EP094110

**Published:** 2026-09-12

**Authors:** Brent James Raiteri, Karla Friederike Bosse, Marta Boccardo, Alain Charles Vandal, Daniel Hahn

**Affiliations:** ^1^ Human Movement Science, Faculty of Sport Science Ruhr University Bochum Bochum North Rhine‐Westphalia Germany; ^2^ School of Human Movement and Nutrition Sciences The University of Queensland Brisbane Queensland Australia; ^3^ Laboratory for Engineering of the Neuromuscular System Department of Electronics and Telecommunication Politecnico di Torino Turin Italy; ^4^ Department of Statistics Faculty of Science University of Auckland Auckland New Zealand

**Keywords:** contraction history, dorsiflexor, firing rate, force depression, force enhancement

## Abstract

EMG‐based muscle force predictions are often inaccurate following active muscle stretch or shortening because of residual force enhancement (rFE) or depression (rFD), respectively, which can alter the neural drive to a muscle. However, the extent of neural drive modulation attributable to rFE or rFD remains unknown owing to the limited spatial sampling of intramuscular EMG. Therefore, we combined two blind‐source separation approaches with high‐density surface EMG and mixed‐effects modelling to assess changes in neural drive from more representative motor unit samples. Seventeen participants performed dorsiflexion contractions at 20% and 40% of maximum voluntary torque in three conditions: stretch‐hold, shortening‐hold and fixed‐end reference (REF) conditions. The ankle dorsiflexion torques and angles were matched using dynamometry to the REF condition during a steady state following a 1‐s 25° stretch or shortening, during which we decomposed tibialis anterior high‐density EMG recordings. Normalised EMG amplitudes were ∼2% lower following stretch and 1% and 3% higher following shortening relative to REF at 20% and 40% of maximum voluntary torque (*P* ≤ 0.008), respectively. Discharge rates from 19 matched motor units per person on average obtained via DEMUSE and MUedit were similar (*P *= 0.666). Following stretch and shortening, discharge rates were ∼1 Hz lower (*P* ≤ 0.029) and 0 (*P *= 0.459) to 1 Hz higher (*P *< 0.001) relative to REF, respectively. More unique motor units were also detected following shortening. These findings indicate that to account for rFE or rFD, neural drive is respectively decreased or increased via reduced or additional motor unit recruitment and discharge rate modulation, with a contraction intensity‐specific rate modulation following active shortening.

## Abstract (highlights)

- **What is the central question of this study?**
  How does a muscle's contraction history (specifically, a prior active stretch or shortening) affect neural drive during steady‐state submaximal voluntary force production?
- **What is the main finding and its importance?**
  We found that the neural drive decreased after active stretch and increased after active shortening, which was confirmed at the motor‐unit level via two blind‐source separation approaches and linear mixed‐effects modelling. Our findings show that the strategy to deal with force deficits following active shortening is contraction‐intensity dependent, with muscle force prediction inaccuracies based on EMG amplitude increasing at a higher intensity.

## 1 INTRODUCTION

A key challenge in biomechanics is the accurate estimation of muscle force, given that direct in vivo measurements in humans remain impossible. Accurate muscle force predictions are crucial for estimating tissue and joint loading (Navacchia et al., 2016) and for informing injury prevention and rehabilitation strategies. However, accurate predictions are challenging because information is required on joint kinematics, activation dynamics and muscle lengths and their rates of change (Pandy et al., 1990; Zajac, 1989). Even for a relatively simple scenario where activation is fixed, steady‐state muscle force predictions are often inaccurate, with force being higher than predicted following active muscle stretch or lower than predicted following active muscle shortening (Edman et al., 1978; Granzier & Pollack, 1989; Leonard et al., 2010; Trecarten et al., 2015). These prediction inaccuracies arise because of two phenomena known as residual force enhancement (rFE) and residual force depression (rFD) (Abbott & Aubert, 1952). rFE and rFD respectively describe the enhanced or depressed steady‐state force following active muscle stretch or shortening relative to the force attained during fixed‐end reference contractions at a matched final muscle length and activation level.

rFE and rFD are typically observed in activation‐matched (*ex vivo*) or EMG‐amplitude‐matched (in vivo) experiments (Abbott & Aubert, 1952; Herzog et al., 1998; Pinniger & Cresswell, 2007), with reported rFE and rFD amplitudes ranging from 7% to 27% and from 6% to 24%, respectively. If force or torque is matched rather than activation/activity level, there is thus the potential for either rFE or rFD to alter the underlying neural drive for a given muscle force, which has implications for accurate prediction of muscle force based on EMG measurements (Lloyd & Besier, 2003). Previous in vivo results from force/torque‐matched experiments indicate lower (5%–44%) or higher (9%–48%) EMG amplitudes in the steady state following active muscle–tendon unit stretch or shortening, respectively (Altenburg et al., 2008; Dalton et al., 2018; Jakobi et al., 2020; Oskouei & Herzog, 2005; Paquin & Power, 2018; Rousanoglou et al., 2007). Consequently, neural drive might change in a similar manner following active stretch or shortening. However, changes in neural drive cannot be inferred reliably from the amplitude of conventional bipolar surface EMG. This limitation arises because peripheral muscle properties (such as muscle fibre length and orientation, motor unit size and territory, and metabolic state) confound EMG amplitude estimations (Dideriksen & Farina, 2019; Dimitrov et al., 2008; Martinez‐Valdes et al., 2018). Instead, to assess neural drive accurately, the individual discharge patterns of active spinal motor units must be identified (Caillet et al., 2024).

Only a couple of studies have used intramuscular EMG to assess neural drive changes following active muscle–tendon unit stretch or shortening. Altenburg et al. (2008) found that vastus lateralis motor unit discharge rates were similar [mean (SD): −0.1 (0.1) Hz; −1 (8)%] or higher [0.7 (0.4) Hz; 11 (9)%] following stretch or shortening, respectively, whereas EMG amplitudes were lower [−16 (12)%] or higher [9 (6)%–19 (14)%] following respective stretch or shortening relative to torque‐matched (4%–50% of maximum) fixed‐end reference contractions. More recently, Jakobi et al. (2020) found that tibialis anterior (TA) motor unit discharge rates were lower (mean range: −2.1 to −3.6 Hz; −19% to −27%) following stretch (shortening was not studied) relative to torque‐matched (10% and 20% of maximum) fixed‐end reference contractions. Additionally, these authors reported lower TA EMG amplitudes and reduced torque steadiness following active muscle–tendon unit stretch versus fixed‐end reference contractions, which was attributed to a net decline in the overall activity of the motoneuron pool [−24 (16)% to −44 (24)%] and not to increased discharge rate or neural drive variability (Jakobi et al., 2020). However, only a relatively limited number of motor units (up to seven) were studied per person, which is a general limitation of intramuscular EMG owing its high spatial selectivity (McGill & Dorfman, 1985). Further studies are thus needed to assess changes in neural drive based on a relatively larger number of active motor units to gain a better understanding of the general behaviour of the motoneuron pool following both stretch and shortening.

One way to identify discharge patterns non‐invasively from a larger number of individual motor units is through the use of decomposition algorithms alongside high‐density surface EMG measurements (Holobar & Zazula, 2004, 2007a). Non‐invasive high‐density surface EMG decomposition has comparable accuracy to intramuscular EMG decomposition for identifying individual motor unit discharge times (Holobar et al., 2009, 2010; Marateb et al., 2011; Negro et al., 2016) and could provide new insight into changes in neural drive following contraction‐history‐dependent changes to the force capacity of a muscle. Therefore, with high‐density surface EMG decomposition, we aimed to determine the extent of neural drive modulation from a larger population of active motor units than studied previously following muscle–tendon unit stretch or shortening contractions compared with torque‐ and joint angle‐matched fixed‐end reference contractions. To increase the likelihood of inducing rFE or rFD, we used an active preload phase prior to muscle–tendon unit stretch or shortening to ensure active muscle stretch or shortening during muscle–tendon unit displacement (Raiteri et al., 2024). We chose to study the human TA muscle because accurate single motor unit discharges can be decomposed up to 70% of maximal voluntary contraction (MVC) for this muscle (Holobar et al., 2014) and because the TA contributes to roughly half of the voluntary dorsiflexion torque based on its physiological cross‐sectional area (Brand et al., 1986). Additionally, the TA undergoes both active stretch and shortening contractions during walking (Maharaj et al., 2019), hence understanding how active length changes affect neural drive to the TA in a carefully controlled experiment could inform us about potential neural drive modulations during everyday locomotion.

Given that this was the first study of contraction history to use high‐density surface EMG, we implemented two distinct blind‐source separation approaches: DEMUSE (Holobar & Zazula, 2007a, 2007b) and MUedit (Avrillon et al., 2024). This allowed us to cross‐validate motor unit identification, quantify algorithm‐related variance and verify the physiological robustness of the neural drive findings. The decomposition comparison included: (1) a conventional, yet subjective, approach using DEMUSE and manual editing; and (2) an approach free of manual editing using MUedit and additional quality controls to eliminate subjectivity and save time. We also explored whether prediction errors of dorsiflexion torque based on EMG amplitude alone would be affected by contraction history and contraction intensity. We hypothesised that (1) the choice of decomposition algorithm would not significantly affect the discharge rate of matched motor units, owing to a high proportion of shared motor units between the two approaches. We also hypothesised that (2) normalised EMG amplitudes and the underlying motor unit discharge rates would be respectively lower or higher during the steady state following active muscle–tendon unit stretch or shortening compared with fixed‐end reference contractions. Additionally, we expected that (3) the decrease in neural drive following stretch would be similar among contraction intensities, whereas the increase in neural drive following shortening would be larger at 40% versus 20% of maximal voluntary torque (MVT). This is because rFE, in relative terms, appears to be independent of contraction intensity (Oskouei & Herzog, 2006; Seiberl et al., 2012), whereas relative rFD appears to increase with increasing contraction intensity (De Ruiter et al., 1998; Raiteri et al., 2024). Furthermore, we expected that (4) changes in EMG amplitudes and matched motor unit discharge rates would be strongly (but not perfectly) and positively correlated. Lastly, based on previous findings (Jakobi et al., 2020), we hypothesised that (5) torque variability, but not motor unit discharge rate variability, would be higher following stretch compared with the fixed‐end reference contractions.

## 2 MATERIALS AND METHODS

### 2.1 Ethical approval

All experimental procedures were approved by the ethics committee of the Faculty of Sport Science at Ruhr University Bochum (EKS V 09/2022) and were conducted in accordance with the *Declaration of Helsinki*, aside from database registration. Free written informed consent was obtained prior to the first testing session.

### 2.2 Sample size calculation

A minimum sample size of 16 was calculated with G*Power (v.3.1.9.7, RRID:SCR_01372637; Faul et al., 2009) to achieve ≥95% power to detect a minimum effect size of interest (*d*_z_) of one between the stretch/shortening‐hold conditions and fixed‐end reference contractions, with a two‐tailed α‐level of 5%. Consequently, 18 healthy and recreationally active participants (7 women) were tested to allow for two dropouts.

### 2.3 Participants

Participants were free of pain, injury and fatigue within their lower limbs at the time of testing. Each participant completed one familiarisation and one testing session, which were separated by 2–7 days [4.5 (1.8) days]. Only one female participant was considered a dropout because she was unable to match the torque traces accurately in the three experimental conditions described below, which resulted in 17 usable datasets {*n *= 17, age: 25 (3) years [min–max: 22–31 years], height: 1.77 (0.06) m [min–max: 1.65–1.85 m]; mass: 74 (9) kg [min–max: 58–90 kg]; 6 women, age: 24 (1) years, height: 1.71 (0.05) m, mass: 66 (7) kg; and 11 men, age: 26 (3) years, height: 1.80 (0.04) m, mass: 78 (7) kg}.

### 2.4 Experimental set‐up

**FIGURE 1:**
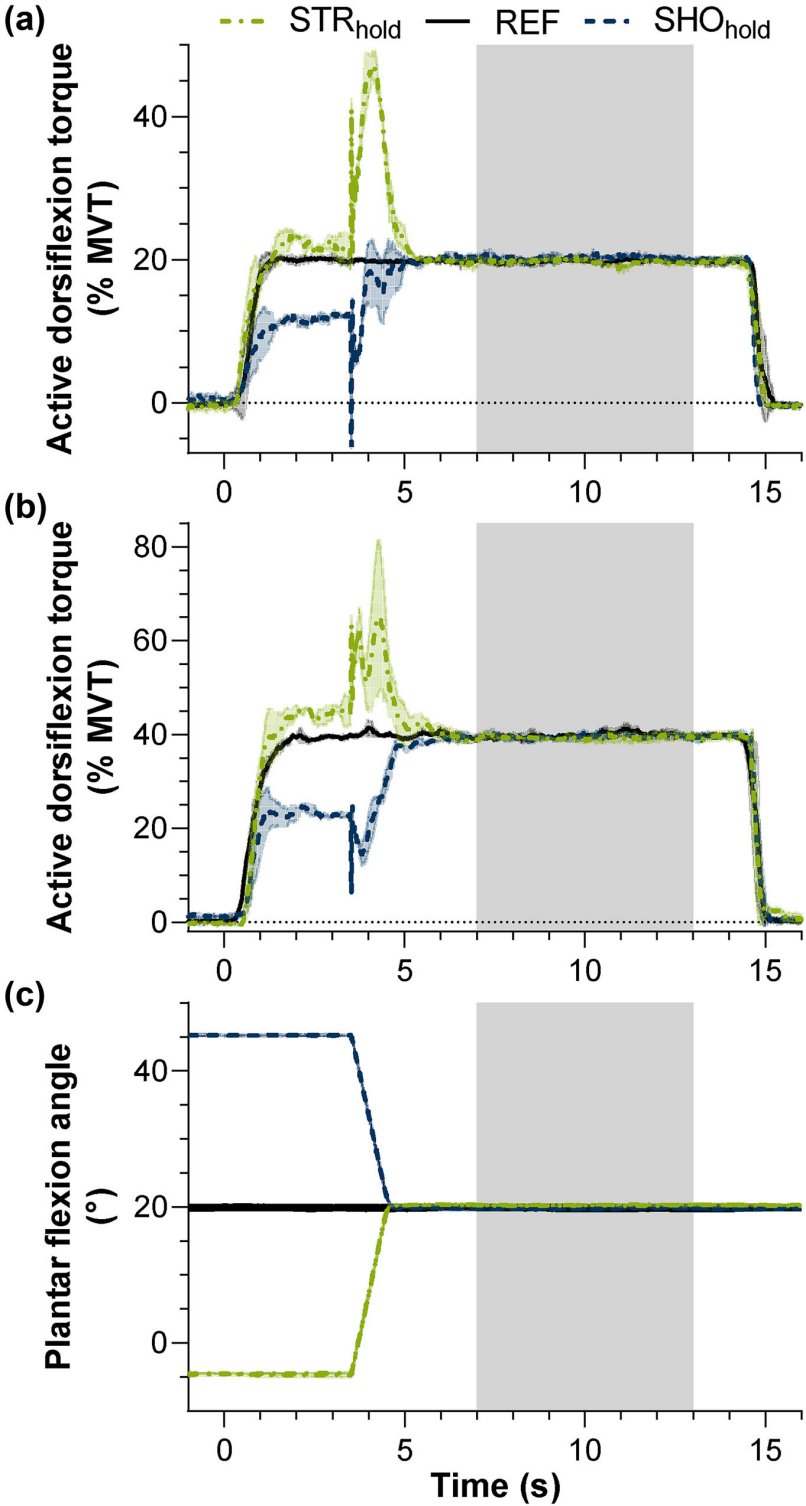
Overview of the tested contraction intensities: (a) 20% of maximal voluntary torque (MVT); (b) 40% MVT; and (c) experimental conditions. The mean traces of multiple trials per condition from one participant are shown, with shaded areas around the mean indicating the SD and the grey shaded block indicating the 6‐s steady‐state phase, which began ∼7 s after contraction onset. Participants matched contraction intensities at 20% MVT (a) and 40% MVT (b) (normalised to 20° plantar flexion) across three distinct conditions (c): stretch‐hold (STR_hold_), fixed‐end reference (REF) and shortening‐hold (SHO_hold_) contractions. For STR_hold_ and SHO_hold_, the contraction onset began with a 1 s ramp up to the desired intensity followed by 2 s of constant pre‐activation, after which the ∼1 s rotation commenced (i.e., ∼4  s before the analysed steady state). Additionally, torque in (a) and (b) prior to the rotation in (c) deviates from 20% and 40% MVT because the joint angle‐specific normalised torque was matched, whereas the illustrated torque traces have been normalised to the MVT produced at 20° plantar flexion; following the rotation, the joint angle‐specific normalised torque at 20° plantar flexion was matched.

The experimental set‐up was almost identical to that already described and shown in Figure 1 of the paper by Raiteri et al. (2023), hence only a brief description will be given here. Participants sat in a reclined posture, with their right hip and knee at ∼110° and ∼90° flexion, respectively, and the sole of their right foot secured to the dorsi/plantar flexion attachment of a motorised dynamometer (IsoMed2000, D&R Ferstl GmbH, Hemau, Germany). A dorsiflexion attachment over the metatarsals ensured that the participant's forefoot maintained constant contact with the dynamometer footplate during voluntary contractions of their right dorsiflexors. This dorsiflexion attachment also limited ankle joint rotation and force contributions from the toe extensors to the measured net ankle dorsiflexion torque owing to its rigidity and location over the foot. Net ankle joint torque and crank arm angle were sampled at 2 kHz by the dynamometer and stored digitally via a Spike2 data collection system (16‐bit Power1401‐3 and 64‐bit Spike2 v.8.23; Cambridge Electronic Design Ltd, Cambridge, UK).

Simultaneously, 64 high‐density surface EMG channels were sampled in monopolar mode from one two‐dimensional grid (13 rows × 5 columns of electrodes with one missing electrode in the top corner, 3 mm recording diameter, 8 mm interelectrode distance; GR08MM1305, OT Bioelettronica, Italy) over the mid‐belly of the TA muscle at 2 kHz by Sessantaquattro hardware (16‐bit resolution with a 10.5 Hz high‐pass first‐order recursive exponentially weighted moving average firmware filter and a 500 Hz low‐pass third‐order digital sinc filter also with frequency notches at 2000 Hz and its harmonics, 1 V/V gain) and stored via OT BioLab+ software (OT Bioelettronica, Italy). Antagonistic muscle activity from the soleus muscle was not assessed based on previous findings of similar EMG amplitudes following active muscle stretch or shortening relative to fixed‐end reference contractions at 20%, 40%, 60% and 80% of maximum (Paquin & Power, 2018). The reference electrode was located above the right fibular head (8 mm recording diameter, Ag/AgCl, Kendall, Mansfield, MA, USA). Electrode and grid placement occurred after the skin was shaved (Teqler, Wecker, Luxembourg), exfoliated (Nuprep, Weaver and Company, Aurora, CO, USA) and cleaned (Sterillium, Hartmann, Heidenheim, Germany), and after the holes of a disposable bioadhesive foam layer, which was positioned between the grid and skin, were filled with conductive cream to enable skin–electrode contact (AC Cream, Spes Medica, Genoa, Italy). EMG data were later synchronised with torque and angle data via a 5 V Transistor‐Transistor Logic  (TTL) pulse with rising and falling edges at the start and end of the recording, respectively, that was output by the Spike2 data collection system and recorded at 2 kHz by both systems.

### 2.5 Experimental protocol

Participants were familiarised with the different experimental conditions during a practice session to improve torque matching during the testing session. In both sessions, participants received real‐time visual feedback of their net ankle joint torque (i.e., 0.01 s moving average) via a screen positioned in front of them. The warm‐up consisted of five fixed‐end dorsiflexion contractions (1 s hold, 1 s rest) at ∼80% of the participant's perceived maximum at 20° plantar flexion (note that 0° plantar flexion represents a 90° angle between the tibia and sole of the foot). Participants then performed MVCs (3–5 s hold) with their right dorsiflexors at −5°, 20° and 45° plantar flexion in a randomised order with standardised verbal encouragement from the investigator. If the difference in peak‐to‐peak torque between two maximal‐effort trials at the same joint angle was >5%, additional maximal‐effort trials were performed until this difference was ≤5%, and a 3 min break was provided after each maximal‐effort trial. The trial with the highest peak‐to‐peak torque (MVT) that was within 5% of another trial at each joint angle was then taken so that participants could match joint‐angle‐specific contraction intensities of 20% and 40% MVT at −5°, 20° and 45° plantar flexion.

Participants subsequently performed three conditions at 20% and 40% MVT. The order of the conditions and the order of the contraction intensities was block randomised, such that all conditions at one contraction intensity were performed before the other contraction intensity, and trials from one condition were repeated before trials of another condition started. The three conditions included two dynamic conditions and one fixed‐end reference (REF) condition, and all conditions had a common final ankle joint angle of 20° plantar flexion. The REF condition involved a 1‐s ramp up to 20% or 40% MVT and a 13‐s hold phase (Figure 1). The stretch‐hold (STR_hold_) and shortening‐hold (SHO_hold_) conditions involved a 1‐s ramp up to 20% or 40% MVT and 2 s of pre‐activation before the ankle dorsiflexor muscle–tendon units were stretched or shortened. The stretch or shortening subsequently occurred over 1 s (i.e., 25° s^−1^) from an ankle joint angle of −5° or 45° plantar flexion to 20° plantar flexion. Following the dynamic phase, a 10‐s hold phase at 20% or 40% MVT was maintained at 20° plantar flexion.

In each condition, participants attempted to keep their net ankle joint torque between two traces that were located 5% above and below the desired contraction intensity. However, participants were instructed to maintain the same perceived effort during the 1‐s stretch or shortening phase owing to the difficulty of matching torque during the dynamic phase (Figure 1). Participants practised each condition once at 20% or 40% MVT before repeating four trials of each condition at the same contraction intensity, resulting in 24 test trials in total. Each trial was followed by a 1‐min break.

### 2.6 Data processing and analysis

#### 2.6.1 Synchronisation

The first data processing step involved using a custom‐written script to export individual trials within each Spike2 recording as separate files with a .mat extension. The recorded digital signals from each trial were then cropped in MATLAB (64‐bit v.R2022a, MathWorks, Natick, MA, USA) between a common start and end time using a voltage‐crossing threshold of 95% of the maximum 5 V TTL pulse recorded. The cropped torque and angle signals of each trial were then combined with the corresponding high‐density surface EMG signals, which were imported into MATLAB using OpenOTBfilesConvFact.m and cropped between a common start and end time using a voltage‐crossing threshold of 10 times the minimum TTL pulse recorded. Different voltage‐crossing thresholds were used to synchronise the data because of different temporal lags [0.5 ms for Spike2 and 33.2 (0.1) ms for OTB], voltage input ranges (10 V for Spike2 and 6.6 V for OTB) and amplitude inversions between systems (positive for Spike2 and negative for OTB).

#### 2.6.2 Filtering and normalisation

As in previous work (Raiteri et al., 2024), recorded torque (20 Hz) and crank arm angle (6 Hz) data were low‐pass filtered using dual‐pass second‐order Butterworth filters that were corrected for two passes (Winter, 2009). A steady‐state passive net ankle joint torque‐angle fit was then constructed to estimate active torque, which involved fitting a second‐order polynomial to the mean filtered crank arm angle and net ankle joint torque data calculated over 1 s at the start of each trial and over 0.25 s at the end of each trial when participants were instructed to relax. Active torques during each trial were subsequently estimated by evaluating the polynomial fit at the crank arm angles recorded during the trial and subtracting these values from the recorded net ankle joint torques. The EMG amplitudes of TA during each trial were calculated by bandpass filtering (20–400 Hz) single‐differential signals with a fourth‐order Butterworth filter, then smoothing these filtered and DC‐removed signals with a centred moving (250 ms or 99.8% overlap) 250.5‐ms root mean square amplitude calculation. The single‐differential signals were calculated from consecutive electrode pairs in the longitudinal direction of the grid, leading to 59 signals for TA EMG amplitude (four of the five columns with 12 bipolar electrode pairs and one column with 11 pairs). Each single‐differential signal was later normalised by its respective value from the MVC with the highest active torque at 20° plantar flexion to determine normalised TA EMG amplitudes. To calculate the 59 normalisation values, initially the time instant of the maximal root mean square amplitude of each signal was calculated, then the centred mean root mean square amplitude over a 500 ms window from this index was determined.

#### 2.6.3 Time averaging

Torque steadiness was calculated as the coefficient of variation (CV) in active torque during the steady state. The steady state was 6 s in duration, defined from 7 s after contraction onset to 1 s before the end of the hold phase (Figure 1). A trial was deemed valid if there was <1.0 Nm active torque difference between the first and last seconds of the steady‐state period. During the steady state of each valid trial, means were calculated for the primary outcome variables (i.e., active torque, torque steadiness, normalised TA EMG amplitude, and TA discharge rate variability), and a single mean of these trial‐specific means was subsequently calculated per condition and contraction intensity. An exception was made for TA discharge rate; to prevent outlier bias from distorting the central tendency, individual motor unit discharge rates were calculated as the median discharge rate during the steady state, followed by calculating a single mean of the trial‐specific medians across: (1) all matched motor units; and (2) all matched and unique motor units (see section 2.6.6).

#### 2.6.4 Motor unit decomposition

Only the steady‐state phase (grey area in Figure 1) of the valid trials was decomposed to improve accuracy by minimising temporal and spatial changes to the waveforms of motor unit action potentials. Trials were excluded from analysis if their mean normalised EMG amplitude fell outside one median absolute deviation of the median from valid trials in that condition to avoid outliers from biasing the results, which resulted in two to five valid trials per condition. The valid trials from each condition were then concatenated together at each contraction intensity to allow the same motor units to be tracked among conditions. Subsequently, the concatenated conditions for decomposition were reformatted (.mat) to be read by the decomposition software of interest and included 6–11 valid trials of 36–66 s duration. Notably, the concatenated trials for decomposition were 1 s longer (i.e., 37–67 s) in duration because the respective start and end of the first and last concatenated trials needed to be padded with 0.5 s of real data to avoid omission during EMG preprocessing by the decomposition software. These files were then imported into the decomposition software, and the raw high‐density surface EMG signals were inspected visually to remove channels with motion artefacts or bad signal‐to‐noise ratios (one channel was removed from two participants at 20% MVT, and one channel was removed from one participant at 40% MVT). The remaining channels (excluding the single missing electrode from the top corner of the 13 × 5 grid, leaving a maximum of 64 channels) were then preprocessed according to the default parameters of DEMUSE (Holobar & Zazula, 2007a, 2007b) and MUedit (Avrillon et al., 2024) (for details, see Supporting Information). The signals were then decomposed into individual motor unit action potentials via blind‐source separation. Of note, the MUedit source code was modified to remove manual cropping, ensuring automated processing across the full file duration. Contraction intensities were decomposed separately to avoid biasing the decomposition results towards higher‐threshold motor units active at 40% but not 20% MVT.

DEMUSE (Holobar & Zazula, 2007a, 2007b) and MUedit (Avrillon et al., 2024) were both used to enhance the robustness of the decomposition results. Their approaches are similar, but not identical, with DEMUSE (v.5.01; University of Maribor, Slovenia) using a gradient convolution kernel compensation algorithm (Holobar & Zazula, 2007b) and MUedit using the method developed by Negro et al. (2016) that incorporates fast independent component analysis (Chen & Zhou, 2016) and gradient convolution kernel‐based source refinement. Nevertheless, both approaches aim to identify a set of filters that distinguish between surface‐recorded motor unit action potentials based on their shape and location to isolate motor unit discharge patterns. Each approach has been validated previously up to 70% (Holobar et al., 2014) or 90% MVT (Negro et al., 2016) by identifying the same motor unit from intramuscular EMG signals and blind‐source separation of the high‐density surface EMG signals recorded from TA. We opted for an extension factor of ∼16 (i.e., 1000 extended signals) for all decompositions, based on the recommendation to reach 1000 signals to maximise the number of identified motor units (Negro et al., 2016), and performed 50 iterations. Other decomposition parameters for DEMUSE and MUedit can be found in the Supporting Information.

#### 2.6.5 Decomposition editing

The decomposition outputs from DEMUSE were edited manually by one expert (M.B.), who was blinded to the experimental conditions but not contraction intensities, and the motor unit discharge patterns were recalculated based on previous guidelines (Del Vecchio et al., 2020; Hug et al., 2021). In line with these guidelines, there was no subjective selection of motor unit discharges in the final decomposition output. Additionally, duplicate motor units in the final output that shared >30% of their identified discharge times (within a time interval of 0.5 ms) were removed, with the motor unit with the lower pulse‐to‐noise ratio being excluded (Holobar et al., 2010).

To eliminate subjective bias and save time, the decomposition outputs from MUedit were edited automatically. This approach was not viable for DEMUSE decompositions, because the CV‐based exclusion led to the removal of most motor units (i.e., leaving only 0–4 remaining, see below). The automatic editing pipeline involved: (1) removing duplicate motor units that shared >30% of their identified discharge times, as described above (Holobar et al., 2010); (2) excluding automatically edited motor units from the MUedit output if they exhibited a pulse‐to‐noise ratio of <28 dB, corresponding to an accuracy of <85% (Holobar et al., 2014); and (3) eliminating motor units with a CV in discharge rate of ≥40% (Kalc et al., 2023). This CV‐based exclusion functioned effectively for MUedit decompositions because, unlike DEMUSE, MUedit iteratively refines separation vectors until the CV of interspike intervals is minimised or falls below 30%. Given that we opted for a pulse‐to‐noise‐ratio metric that does not rely on the regularity of a motor unit discharge pattern to estimate identification accuracy, we also implemented the CV threshold to remove mixed or clustered discharge patterns selectively (Negro et al., 2016). The motor unit discharge patterns were subsequently inspected visually, but manual editing (i.e., addition of missing or removal of falsely identified discharge times) was not performed, in order to preserve objectivity. The agreement between the manually edited DEMUSE decomposition results and the automatically edited MUedit decomposition results can be found in the Supporting Information.

#### 2.6.6 Cumulative spike train

To characterise the neural drive of both matched (i.e., common across experimental conditions) and unique motor units, the cumulative spike train (CST) was calculated for each trial by summing the binarised spike trains across all decomposed and edited motor units from each decomposition approach. A unique motor unit was defined as a retained motor unit identified in only one or two conditions at a given contraction intensity. The resulting CST was smoothed via convolution with a normalised, 0.4 s Hann window. To eliminate boundary distortions caused by filter edge artefacts, 0.2 s were discarded from both the beginning and the end of each filtered CST. Subsequently, to calculate the mean motor unit discharge rate, the smoothed and cropped CST was summed, divided by the number of active motor units, and normalised over the 6 s steady state. Consistent with the median discharge rate analysis applied to matched motor units, the mean discharge rate was converted to a median discharge rate by multiplying the mean value by a trial‐specific median‐to‐mean correction factor (i.e., the median value divided by the mean value from the filtered and cropped CST). Lastly, a single mean of the trial‐specific medians was calculated per condition and contraction intensity for the CST‐derived discharge rate.

### 2.7 Statistics

A two‐tailed α‐level was set at 5%, and statistical analysis was performed in R (v.4.2.2; R Core Team, 2022). To account for the nested structure of the data and the varying numbers of motor units identified per participant, we fitted linear mixed‐effects models using the lme4 package (Bates et al., 2015). Estimation was performed via maximum likelihood with the nloptwrap optimiser. Active torque, torque steadiness, normalised EMG amplitude, discharge rate and discharge rate variability served as primary outcomes, with participant identity specified as a random intercept to account for repeated measures.

For active torque and torque steadiness, the models included random intercepts and random slopes for contraction intensity, condition and their two‐way interaction, grouped by participant (Table 1). For normalised EMG amplitude, the model included random intercepts for participant and channel nested within participant, with fixed slopes for contraction intensity, condition and their two‐way interaction (Table 2); fixed slopes were used because the number of observations was insufficient to support a more complex, maximal random‐effects structure. For the discharge rate and discharge rate variability of matched motor units, the models included random intercepts and random slopes for the decomposition algorithm, contraction intensity, condition and the contraction intensity × condition interaction, allowing these effects to vary uniquely across motor units nested within participants (Table 3). The decomposition algorithm was modelled as a fixed effect with a random slope to capture the average algorithmic difference and simultaneously account for how this difference varied randomly across participants. For the CST‐derived discharge rate of matched and unique motor units, the model included random intercepts and random slopes for the same fixed factors, grouped by participant (Table 3).

**TABLE 1:** Summary of mixed‐effects models results from the active torque measurements.

Parameter	Intercept	40% MVT	STR_hold_	SHO_hold_	40% MVT × STR_hold_	40% MVT × SHO_hold_
Active torque, Nm						
Estimate (SE)	8.10 (0.463)	8.06 (0.421)	−0.08 (0.066)	0.07 (0.059)	−0.01 (0.073)	−0.03 (0.074)
*P*‐value	<0.001	<0.001	0.228	0.287	0.943	0.650
CV in active torque, %						
Estimate (SE)	1.64 (0.131)	−0.07 (0.159)	0.16 (0.067)	0.10 (0.084)	−0.16 (0.080)	−0.05 (0.104)
*P*‐value	<0.001	0.666	0.029	0.238	0.061	0.639

*Note*: The mixed‐effects models evaluated the fixed effects of contraction intensity (20% and 40% MVT), condition (REF, STR_hold_ and SHO_hold_) and the contraction intensity × condition interaction. The models incorporated random intercepts and random slopes grouped by participant [R formula: outcome ∼ intensity * condition + (intensity * condition | participant_ID)]. Sandwich variance estimation‐based standard errors and *P*‐values are presented. The reference level was set to the REF condition at 20% MVT. *n *= 17.

Abbreviations: CV, coefficient of variation; MVT, maximum voluntary torque; SE, robust standard error.

**TABLE 2:** Summary of mixed‐effects model results from the high‐density EMG amplitude measurements.

Parameter	Intercept	40% MVT	STR_hold_	SHO_hold_	40% MVT × STR_hold_	40% MVT × SHO_hold_
Normalised EMG amplitude, % MVC						
Estimate (SE)	18.40 (1.110)	11.60 (0.753)	−1.55 (0.399)	1.21 (0.458)	−0.19 (0.683)	2.03 (0.738)
*P*‐value	<0.001	<0.001	0.001	0.018	0.788	0.014

*Note*: The mixed‐effects model evaluated the fixed effects of contraction intensity (20% and 40% MVT), condition (REF, STR_hold_ and SHO_hold_) and the contraction intensity × condition interaction. The model incorporated random intercepts and fixed slopes grouped by participant, with individual channels nested within participants [R formula: outcome ∼ intensity * condition + (1 | participant_ID/channel_ID)]. Sandwich variance estimation‐based standard errors and *P*‐values are presented. The reference level was set to the REF condition at 20% MVT. *n *= 17.

Abbreviations: MVC, maximal voluntary contraction; MVT, maximum voluntary torque; SE, robust standard error.

**TABLE 3:** Summary of mixed‐effects models results from the high‐density EMG decompositions.

Parameter	Intercept	MUedit	40% MVT	STR_hold_	SHO_hold_	40% MVT × STR_hold_	40% MVT × SHO_hold_
Discharge rate of matched motor units, Hz							
Estimate (SE)	12.50 (0.361)	−0.04 (0.085)	2.29 (0.216)	−1.27 (0.239)	−0.01 (0.182)	0.43 (0.366)	1.00 (0.251)
*P*‐value	<0.001	0.666	<0.001	<0.001	0.970	0.259	0.001
CV in discharge rate of matched motor units, %							
Estimate (SE)	12.40 (0.617)	3.65 (0.272)	2.88 (0.503)	0.73 (0.604)	−0.40 (0.374)	−0.72 (0.668)	−0.53 (0.417)
*P*‐value	<0.001	<0.001	<0.001	0.248	0.302	0.296	0.222
CST‐derived discharge rate of matched and unique motor units, Hz							
Estimate (SE)	11.50 (0.352)	−0.68 (0.105)	1.98 (0.242)	−1.16 (0.244)	0.12 (0.160)	0.49 (0.381)	1.02 (0.224)
*P*‐value	<0.001	<0.001	<0.001	<0.001	0.460	0.219	<0.001

*Note*: The mixed‐effects models evaluated the fixed effects of decomposition algorithm (DEMUSE and MUedit), contraction intensity (20% and 40% MVT), condition (REF, STR_hold_ and SHO_hold_) and the contraction intensity × condition interaction. To analyse matched motor units, models incorporated random intercepts and random slopes grouped by participant, with individual motor units nested within participants [R formula: outcome ∼ decomposition + intensity * condition + (decomposition + intensity * condition | participant_ID / motor_unit_ID)]. To analyse matched and unique motor units via the cumulative spike train, the model incorporated random intercepts and random slopes grouped by participant [R formula: outcome ∼ decomposition + intensity * condition + (decomposition + intensity * condition | participant_ID)]. Sandwich variance estimation‐based standard errors and *P*‐values are presented. The reference level was set to the REF condition at 20% MVT following DEMUSE decomposition. *n *= 17.

Abbreviations: CV, coefficient of variation; CST, cumulative spike train; MVT, maximum voluntary torque; SE, robust standard error.

To address potential violations of residual normality or homoscedasticity across contraction intensities, cluster‐robust standard errors (type ‘CR2’, owing to the small number of clusters) and *P*‐values were calculated using the clubSandwich package (Pustejovsky et al., 2025). Significant contraction intensity × condition interactions were decomposed by conducting three *post hoc* pairwise comparisons at each contraction intensity using the emmeans package (Lenth et al., 2025). Holm‐adjusted *P*‐values were used to control the family‐wise error rate at 5%. Degrees of freedom were estimated using the Satterthwaite approximation within the lmerTest package (Kuznetsova et al., 2017). To predict the normalised active torque from normalised EMG amplitude and quantify the error when using EMG amplitude alone to predict the steady‐state torques in STR_hold_ and SHO_hold_ from the REF condition, a linear mixed‐effects model was fitted. The model included fixed slopes for normalised EMG amplitude and random intercepts grouped by participant. Lastly, four repeated‐measures Pearson correlation coefficients were calculated using the rmcorr package (Bakdash & Marusich, 2017) to assess the strength of the linear relations between mean normalised EMG amplitude and mean discharge rate of matched motor units at each contraction intensity for each decomposition approach. Unless stated otherwise, data are presented as the mean (SD).

## 3 RESULTS

### 3.1 Trial exclusion

A total of 25 (1) [range: 24–29] submaximal voluntary contractions per participant were recorded and subsequently reduced to 17 (2) [range: 15–21] based on the exclusion criteria for motor unit decomposition. This resulted in an average of 3 (1) analysed trials [range: 2–5] per condition. No additional trials were excluded owing to an active torque difference of ≥1 Nm between the first and last seconds of the analysed steady state, probably because participants were familiarised with the conditions and torque matching in a prior session.

### 3.2 Experimental order

Participants performed 7 (1) MVCs [range: 6–8] in total prior to the submaximal voluntary trials. Although the order of contraction intensities was counterbalanced across participants, with 9 of 17 participants completing the 20% MVT condition first, an order effect emerged across conditions. At 20% MVT, SHO_hold_ was most frequently performed last (first: *n *= 4, second: *n *= 6, last: *n *= 7). The reverse was true for STR_hold_ (first: *n *= 7, second/last: *n *= 5 each). At 40% MVT, this pattern reversed: SHO_hold_ was performed predominantly first (first: *n *= 8, second: *n *= 5, last: *n *= 4), and STR_hold_ was least frequently performed first (first: *n *= 3, second/last: *n *= 7 each).

### 3.3 Active torque

The maximum active dorsiflexion torques at the reference angles of −5°, 20° and 45° plantar flexion were 41.3 (9.7), 41.0 (9.6) and 27.5 (6.9) Nm, respectively. At 20% MVT, mean active torque was 7.6 (7.1) Nm (∼50%) higher during stretch and −5.4 (4.6) Nm lower (∼−35%) during shortening compared with REF. At 40% MVT, active torque was 13.4 (10.9) Nm (∼50%) higher during stretch and −5.1 (7.1) Nm lower (∼−20%) during shortening compared with REF. Importantly, because torque was an independent variable that participants matched across conditions during the steady state, statistical analysis confirmed that contraction intensity significantly affected steady‐state active torque (*P *< 0.001), whereas condition had no significant effect (*P* ≥ 0.228; Table 1). Additionally, no significant interaction was found between contraction intensity and condition (*P* ≥ 0.650; Table 1), and the mean steady‐state active torque differences among conditions (∼0.1 Nm) fell within the measurement error of the torque sensor. The random‐effects estimates revealed that the active torque at 20% MVT in REF was slightly less consistent across participants [σ^2^ = 3.4 Nm^2^ (1.8 Nm)] than the individual responses to contraction intensity [σ^2^ = 2.8 Nm^2^ (1.7 Nm); see Supporting Information]. Unsurprisingly, participants who produced higher active torques at 20% MVT also produced a larger increase in active torque to attain 40% MVT (*r *= 0.98).

### 3.4 Torque steadiness

In line with our expectations, condition significantly affected torque steadiness in STR_hold_ (*P = *0.029), but there was no significant effect of contraction intensity (*P *= 0.666), and no significant interaction was found between contraction intensity and condition (*P* ≥ 0.061; Table 1). However, *post hoc* pairwise comparisons revealed that the CV in active torque was not significantly higher (i.e., lower torque steadiness) in STR_hold_ than REF at 20% [∆CV = 0.16 (0.28), *P *= 0.053] or 40% MVT [∆CV < 0.01 (0.22), *P *= 0.999], against our expectations. The CV in active torque was also not significantly higher in SHO_hold_ than REF at 20% [∆CV = 0.10 (0.35), *P *= 0.470] or 40% MVT [∆CV = 0.05 (0.28), *P *= 0.999]. The random‐effects estimates revealed that contraction intensity had the least consistent effect on torque steadiness across participants [σ^2^ = 0.32%^2^ (0.57%)], and surprisingly, participants who produced lower torque steadiness (higher CV) at 20% MVT generally showed the largest increase in torque steadiness when transitioning to 40% MVT (*r *= −0.82; see Supporting Information).

### 3.5 EMG amplitude

**FIGURE 2:**
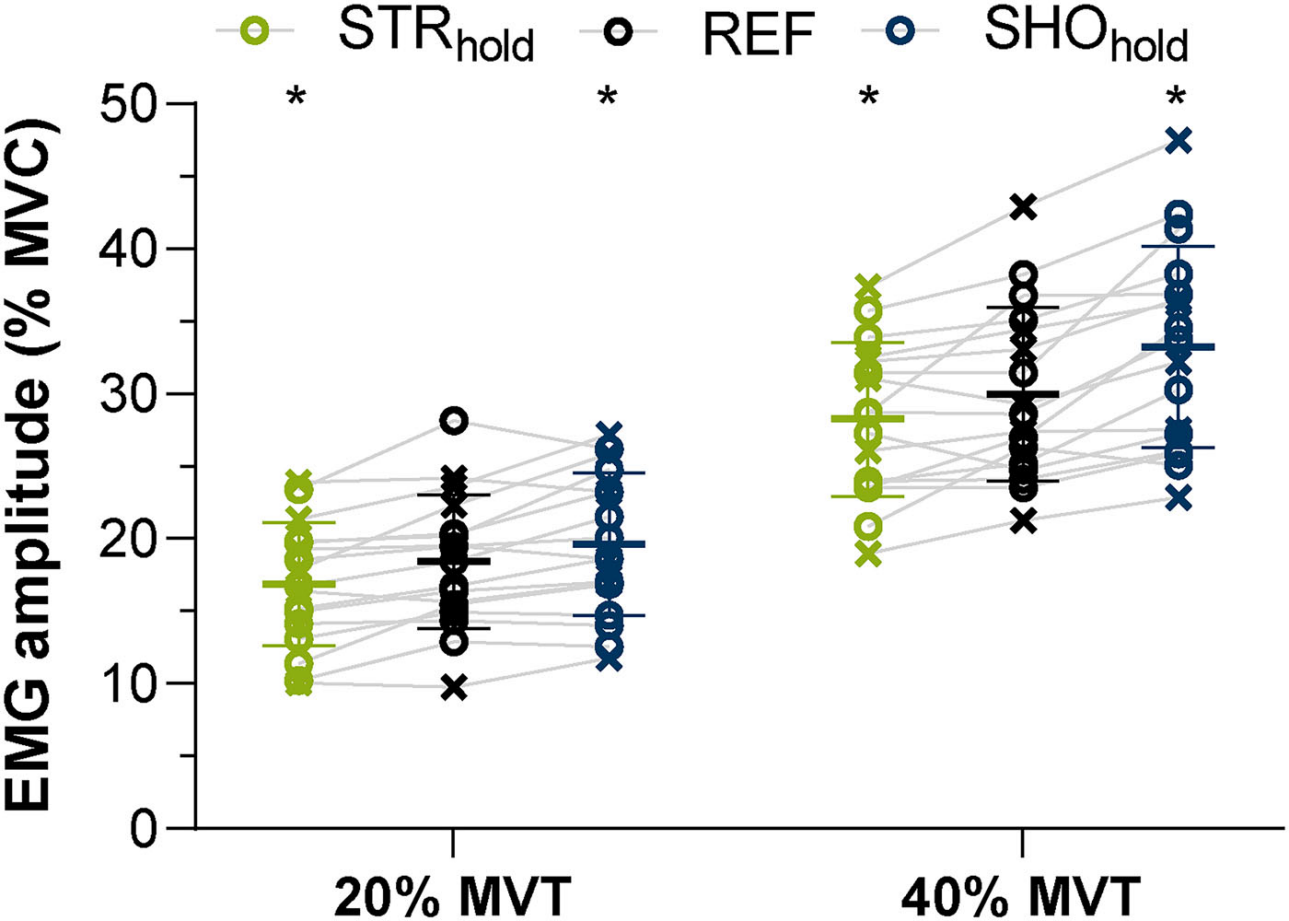
Normalised tibialis anterior EMG amplitude at 20% and 40% of maximum voluntary torque (MVT) during the steady‐state phase in three conditions: stretch‐hold (STR_hold_), fixed‐end reference (REF) and shortening‐hold (SHO_hold_) contractions. Individual participant means (dots; *n *= 17), estimated marginal means (thick horizontal lines) and SDs (thin horizontal lines) are shown, with grey lines linking each participant across conditions. Women are represented by crosses (*n *= 6) and men by open circles (*n *= 11). Asterisks indicate a significant mean difference compared with REF at that specific contraction intensity (*P* ≤ 0.008; see Results text). There was a significantly increased slope between REF and SHO_hold_ at 40% versus 20% MVT (*P* = 0.014).

Contraction intensity (*P *< 0.001) and condition (*P* ≤ 0.018; Table 2) both significantly affected normalised TA EMG amplitude. The effect of condition also depended significantly on the contraction intensity in SHO_hold_ (*P *= 0.014; Table 2). *Post hoc* pairwise comparisons revealed that the increase in EMG amplitude from 20% to 40% MVT was greater in SHO_hold_ [∆ = 13.6 (3.5)% MVC, *t*(5015) = 15.97, *P *< 0.001] than in REF [∆ = 11.6 (3.1)% MVC, *t*(5015) = 15.37, *P *< 0.001] and STR_hold_ [∆ = 11.4 (2.4)% MVC, *t*(5015) = 19.48, *P *< 0.001], as expected. Additionally, EMG amplitude was lower in STR_hold_ than REF at both contraction intensities {20% MVT: ∆ = −1.6 (1.6)% MVC, *t*(5015) = −3.88, *P *< 0.001, 95% confidence interval (CI) [−2.5 to −0.6]; and 40% MVT: ∆ = −1.7 (2.7)% MVC, *t*(5015) = −2.64, *P *= 0.008, 95% CI [−3.3 to −0.2]}, and EMG amplitude was higher in SHO_hold_ than REF at both contraction intensities {20% MVT: ∆ = 1.2 (1.9)% MVC, *t*(5015) = 2.64, *P *= 0.008, 95% CI [0.1 to 2.3]; and 40% MVT: ∆ = 3.2 (2.7)% MVC, *t*(5015) = 4.87, *P *< 0.001, 95% CI [1.6 to 4.8]; Figure 2}.

### 3.6 Torque error

Normalised EMG amplitude was a significant, positive predictor of normalised active torque in the REF condition {*r*_rm_(16) = 0.97, 95% CI [0.91 to 0.99]}. For every 1% increase in EMG amplitude, active torque increased by 1.45% MVT {SE = 0.12; *P *< 0.001, 95% CI [1.22 to 1.69]}. Using this global predictive model and the normalised EMG amplitude differences with respect to REF led to mean torque errors of −2.2% and −2.5% MVT at 20% and 40% MVT in STR_hold_, respectively, whereas mean errors in SHO_hold_ were 1.8% and 4.7% MVT, respectively. Consequently, the torque error magnitude in SHO_hold_ at 40% MVT was almost double that observed in STR_hold_ and in SHO_hold_ at 20% MVT. Across individual participants, the torque error range in STR_hold_ was −7% to 1% MVT at 20% MVT and −12% to 4% MVT at 40% MVT, whereas the error range in SHO_hold_ shifted to −3% to 7% MVT at 20% MVT and to −2% to 14% MVT at 40% MVT. Notably, the individual error ranges expanded at the higher contraction intensity, indicating that subject‐specific torque prediction variance increased from 20% to 40% MVT.

### 3.7 Decomposition output

The number of identified motor units and their respective discharges were arithmetically higher for MUedit than DEMUSE, and arithmetically more motor units were identified at 20% than 40% MVT across both algorithms (Table 4). Regarding the quality of the source separation, the pulse‐to‐noise ratio was arithmetically higher for MUedit than DEMUSE, whereas the reverse was true for the CV in discharge rate (Table 5). The quality of the source separation based on the silhouette values was arithmetically identical between the two algorithms (Table 5). CV‐based exclusion of decomposed motor units from MUedit resulted in the removal of 5 (2) motor units at 20% MVT and 4 (3) motor units at 40% MVT. In comparison, pulse‐to‐noise ratio‐based exclusion led to the removal of 5 (5) and 5 (4) motor units, respectively. The percentage of motor units common to both exclusion metrics was 42 (33)% at 20% MVT and 62 (33)% at 40% MVT.

**TABLE 4:** Number of motor units and identified discharges between participants for each algorithm and shared between algorithms at each contraction intensity and combined.

Algorithm	Combined		20% MVT		40% MVT	
	Total MU #	Total discharge #	MU #	Discharge #	MU #	Discharge #
DEMUSE	503	340 581	16 (5) [8–31]	10 103 (3958)	13 (5) [5–22]	9932 (3614)
MUedit	778	512 550	24 (9) [8–37]	14 650 (6173)	21 (9) [8–35]	15 500 (6664)
Shared	456	91%	15 (6) [6–30]	91 (7) [75%–100%]	12 (5) [4–21]	87 (10) [67%–100%]

*Note*: The shared row under discharge # indicates the percentage of shared motor units. Values are between‐subject means (SD) [minimum–maximum] except for combined, which is the between‐subject sum; *n* = 17.

Abbreviations: MU, motor unit; MVT, maximum voluntary torque.

**TABLE 5:** Pulse‐to‐noise ratios and silhouette values of the identified motor units between participants for each algorithm at each contraction intensity.

Algorithm	20% MVT			40% MVT		
	PNR (dB)	SIL	CV in DR (%)	PNR (dB)	SIL	CV in DR (%)
DEMUSE	32 (3) [28–37]	0.93 (0.02) [0.87–0.96]	13 (2) [10–17]	31 (2) [27–35]	0.92 (0.02) [0.88–0.94]	14 (3) [10–19]
MUedit	34 (2) [31–38]	0.93 (0.01) [0.91–0.95]	16 (2) [14–20]	33 (2) [29–35]	0.92 (0.01) [0.91–0.94]	19 (2) [15–25]

*Note*: Values are between‐subject means (SD) [minimum—maximum]; *n *= 17.

Abbreviations: CV, coefficient of variation; DR, discharge rate; MVT, maximum voluntary torque; PNR, pulse‐to‐noise ratio; SIL, silhouette value.

At the same contraction intensity, both decomposition outputs shared a large proportion of their identified motor units [range: 67%−100%] (Table 4), which was expected given their similar blind‐source separation approaches. Unsurprisingly, a low proportion of shared motor units was observed using the same algorithm across the two contraction intensities, yielding 18 (8)% [range: 0%−29%] shared units for DEMUSE and 18 (11)% [range: 0%−42%] shared units for MUedit. This low yield of shared motor units across contraction intensities justifies the methodological decision to perform separate decompositions at each contraction intensity.

### 3.8 Discharge rate

#### 3.8.1 Matched motor units

Calculating the mean versus the median discharge rate per motor unit across participants revealed that the mean shifted the estimated discharge rate upwards by 0.12 (0.15) Hz [range: −0.99 to 1.75 Hz] for the manually edited DEMUSE decompositions and by 0.02 (0.25) Hz [range: −1.46 to 1.56 Hz] for the automatically edited MUedit decompositions. Given that this overestimation introduced by the mean calculation was inconsistent between the two decomposition approaches, the median discharge rate per motor unit was used in the analysis to avoid introducing a mean‐driven algorithm bias.

The choice of decomposition algorithm did not significantly affect the TA motor unit discharge rate of matched units (*P *= 0.666; Table 3). Consequently, the estimated marginal means reported below reflect the average between decomposition algorithms. Contraction intensity (*P *< 0.001) and condition (STR_hold_: *P *< 0.001, SHO_hold_: *P *= 0.970; Table 3) both significantly affected discharge rate. The effect of condition also depended significantly on the contraction intensity in SHO_hold_ (*P *= 0.001). *Post hoc* pairwise comparisons revealed that the increase in discharge rate from 20% to 40% MVT was greater in SHO_hold_ [∆ = 3.3 (0.9) Hz, *t*(13.3) = 15.30, *P *< 0.001] than in REF [∆ = 2.3 (0.9) Hz, *t*(16.7) = 10.60, *P *< 0.001] and STR_hold_ [∆ = 2.7 (1.2) Hz, *t*(15.0) = 9.76, *P *< 0.001], as expected. Additionally, discharge rate was lower in STR_hold_ than REF at both contraction intensities {20% MVT: ∆ = −1.3 (1.0) Hz, *t*(16.7) = −5.31, *P *< 0.001, 95% CI [−1.9 to −0.6]; and 40% MVT: ∆ = −0.8 (1.1) Hz, *t*(17.2) = −3.23, *P *= 0.005, 95% CI [−1.5 to −0.2]}. Surprisingly, however, discharge rate was higher in SHO_hold_ than REF only at 40% MVT, but not at 20% MVT {20% MVT: ∆ = 0.0 (0.8) Hz, *t*(16.9) = 0.04, *P *= 0.970, 95% CI [−0.5 to 0.5]; and 40% MVT: ∆ = 1.0 (1.0) Hz, *t*(16.1) = 4.08, *P *= 0.002, 95% CI [0.3 to 1.6]}.

The random‐effects estimates revealed that contraction intensity had the least consistent effect on discharge rate across individual motor units [σ^2^ = 3.4 Hz^2^ (1.9 Hz)], followed closely by decomposition algorithm [σ^2^ = 3.0 Hz^2^ (1.7 Hz); see Supporting Information]. Across participants, contraction intensity [σ^2^ = 0.5 Hz^2^ (0.7 Hz)] and decomposition algorithm [σ^2^ < 0.1 Hz^2^ (0.2 Hz)] had more consistent effects on discharge rate based on lower variance. The opposite pattern emerged for condition, whereby condition had a less consistent effect on discharge rate across participants [STR_hold_: σ^2^ = 0.9 Hz^2^ (0.9 Hz), SHO_hold_: σ^2^ = 0.5 Hz^2^ (0.7 Hz)], but a remarkably consistent effect across individual motor units [STR_hold_: σ^2^ < 0.1 Hz^2^ (0.2 Hz), SHO_hold_: σ^2^ < 0.1 Hz^2^ (0.1 Hz)].

**FIGURE 3:**
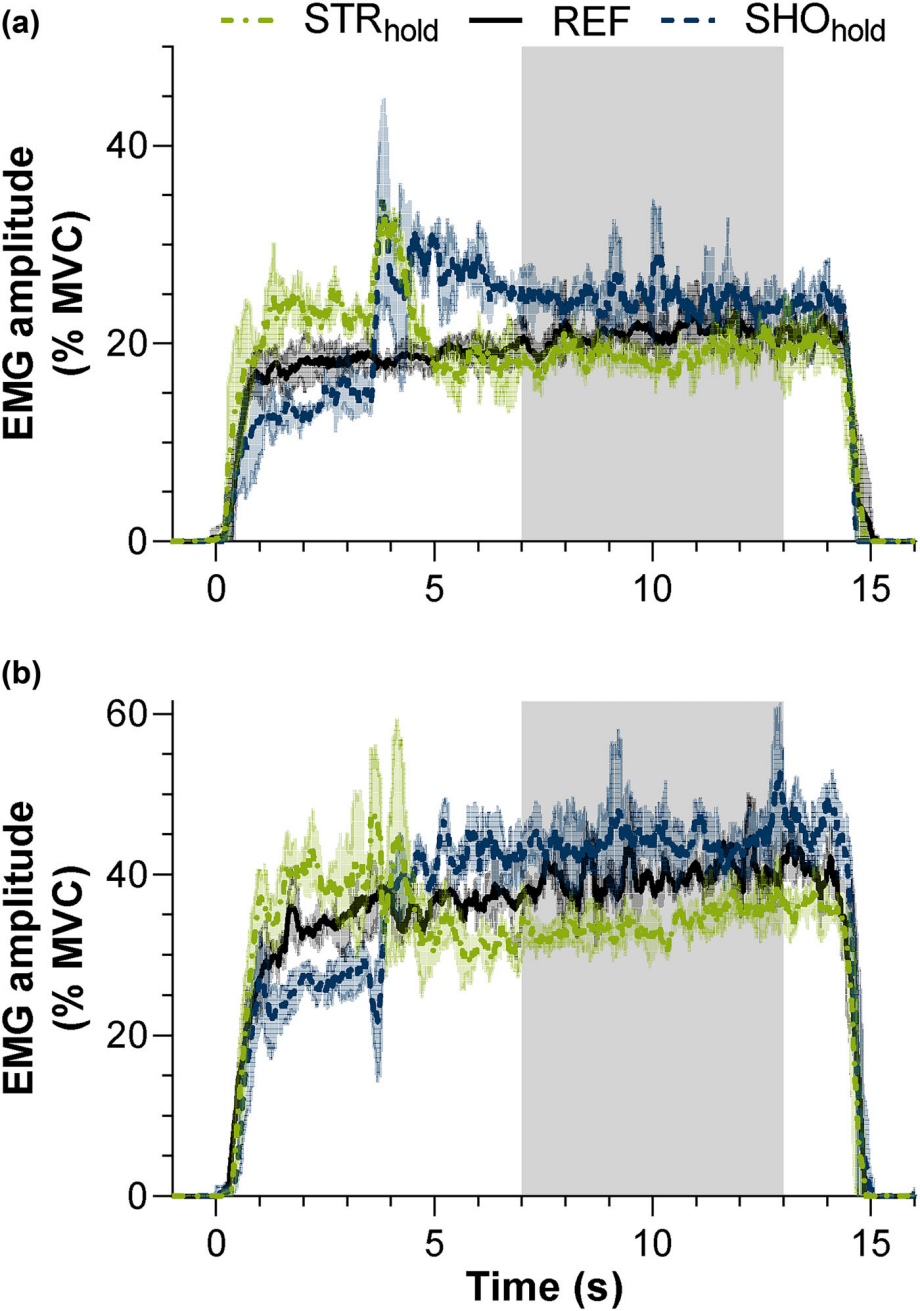
Normalised tibialis anterior EMG amplitudes over time at 20% of maximum voluntary torque (MVT; a) and 40% of MVT (b) in three conditions: stretch‐hold (STR_hold_), fixed‐end reference (REF) and shortening‐hold (SHO_hold_) contractions. The mean traces of multiple trials per condition from one participant (same as in Figure 1) are shown, with shaded areas around the means indicating the SD and the grey shaded area indicating the 6‐s steady‐state phase. The normalised EMG amplitude was calculated as the mean of the 59 single‐differential EMG signals, which were previously normalised to the channel‐specific maximal EMG amplitude from the MVC with the highest active torque at 20° plantar flexion.

The repeated‐measures linear relationships between normalised TA EMG amplitude (for representative time traces, see Figure 3) and TA discharge rate of matched motor units were strong and positive at each contraction intensity and for each decomposition algorithm. However, these relationships were slightly weaker at 20% MVT {DEMUSE: *r*_rm_(33) = 0.84, 95% CI [0.70 to 0.92], *P *< 0.001; and MUedit: *r*_rm_(33) = 0.85, 95% CI [0.71 to 0.92], *P *< 0.001} than at 40% MVT {DEMUSE: *r*_rm_(33) = 0.92, 95% CI [0.85 to 0.96], *P *< 0.001; and MUedit: *r*_rm_(33) = 0.93, 95% CI [0.86 to 0.96], *P *< 0.001}. This discrepancy probably occurred because discharge rate did not increase in line with EMG amplitude at 20% MVT in SHO_hold_.

#### 3.8.2 Matched and unique motor units

**FIGURE 4:**
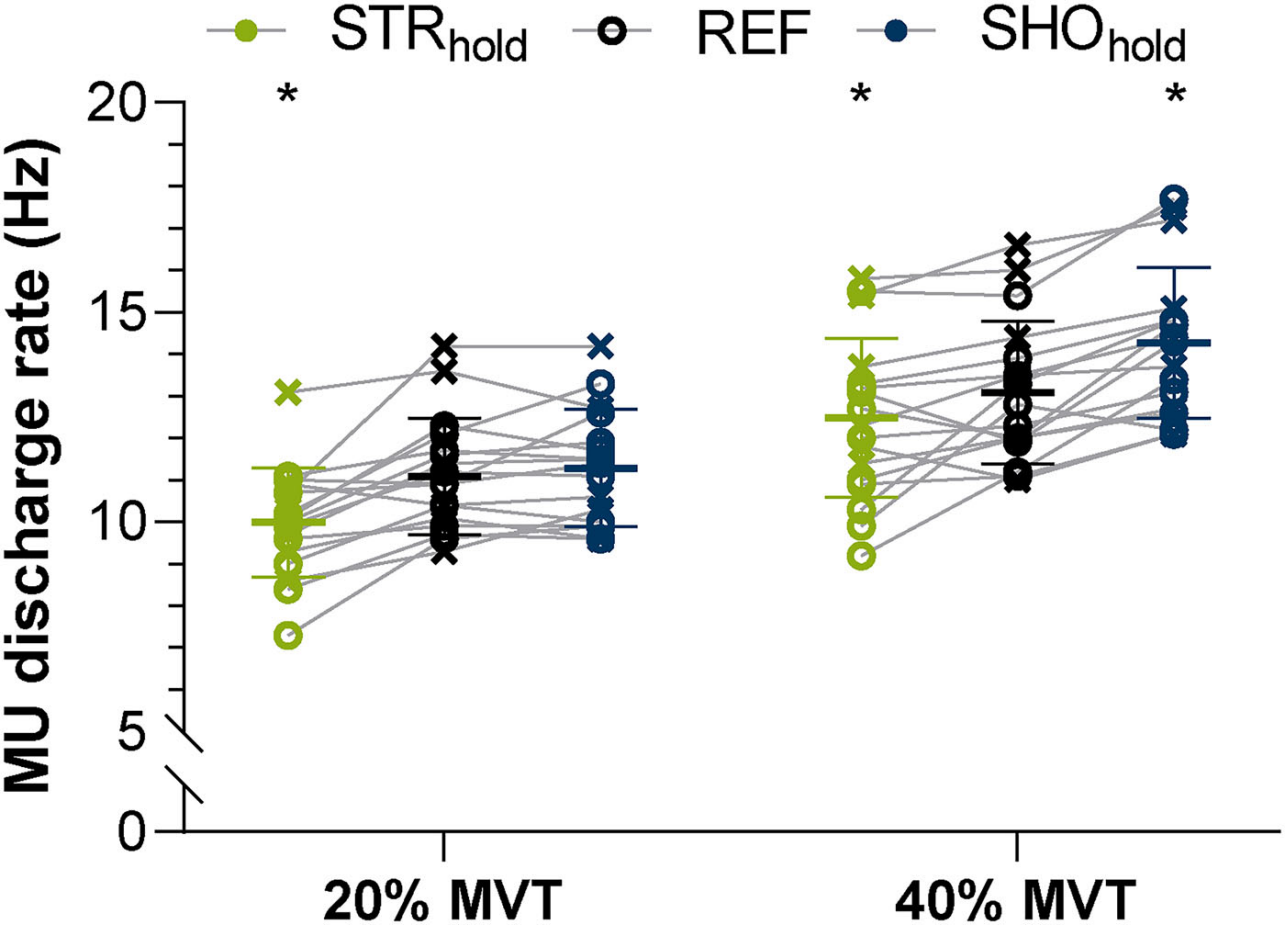
Tibialis anterior motor unit (MU) discharge rates of matched and unique units at 20% and 40% of maximum voluntary torque (MVT) during the steady‐state phase in three conditions: stretch‐hold (STR_hold_), fixed‐end reference (REF) and shortening‐hold (SHO_hold_) contractions. Individual participant means (dots; *n *= 17), estimated marginal means (thick horizontal lines) and SDs (thin horizontal lines) are shown, with grey lines linking each participant across conditions. Women are represented by crosses (*n *= 6) and men by open circles (*n *= 11). Asterisks indicate a significant mean difference compared with REF at that specific contraction intensity (*P* ≤ 0.029; see Results text). There was a significantly increased slope between REF and SHO_hold_ at 40% versus 20% MVT (*P *< 0.001).

The neural drive of matched and unique motor units was estimated based on the individual median discharge rates determined via the smoothed CST. The choice of decomposition algorithm significantly affected the CST‐derived TA motor unit discharge rate (*P *< 0.001; Table 3), with MUedit showing a consistently lower rate than DEMUSE among conditions. Contraction intensity (*P *< 0.001) and condition (STR_hold_: *P *< 0.001, SHO_hold_: *P *= 0.460; Table 3) also both significantly affected the CST‐derived TA discharge rate. The effect of condition also depended significantly on the contraction intensity in SHO_hold_ (*P *< 0.001). *Post hoc* pairwise comparisons averaged between decomposition algorithms revealed the same significant results as the discharge rate analysis on matched motor units, including a greater increase in discharge rate from 20% to 40% MVT in SHO_hold_ [∆ = 3.0 (0.7) Hz, *t*(17.0) = 17.19, *P *< 0.001] than in REF [∆ = 2.0 (1.0) Hz, *t*(17.1) = 8.17, *P *< 0.001] and STR_hold_ [∆ = 2.5 (1.4) Hz, *t*(17.0) = 7.41, *P *< 0.001]. Additionally, discharge rate was lower in STR_hold_ than REF at both contraction intensities {20% MVT: ∆ = −1.2 (1.0) Hz, *t*(17.0) = −4.74, *P *= 0.001, 95% CI [−1.8 to −0.5]; and 40% MVT: ∆ = −0.7 (1.2) Hz, *t*(17.0) = −2.39, *P *= 0.029, 95% CI [−1.4 to −0.1]}, and discharge rate was higher in SHO_hold_ than REF only at 40% MVT, but not at 20% MVT {20% MVT: ∆ = 0.1 (0.7) Hz, *t*(17.6) = 0.76, *P *= 0.459, 95% CI [−0.3 to 0.5]; and 40% MVT: ∆ = 1.1 (0.9) Hz, *t*(17.0) = 5.52, *P *< 0.001, 95% CI [0.6 to 1.7]; Figure 4}.

### 3.9 Discharge rate variability

The choice of decomposition algorithm significantly affected discharge rate variability of the matched motor units (*P *< 0.001; Table 3), with MUedit exhibiting a higher CV in discharge rate than DEMUSE (Table 5). Contraction intensity also significantly affected discharge rate variability (*P *< 0.001; Table 3), with increased variability at 40% than 20% MVT (Table 5). However, against our expectations, condition did not significantly affect discharge rate variability (*P ≥* 0.248; Table 3). Furthermore, no significant interaction was found between contraction intensity and condition (*P* ≥ 0.222; Table 3).

The random‐effects estimates revealed that decomposition algorithm [σ^2^ = 8.56%^2^ (2.93%)] and contraction intensity [σ^2^ = 6.71%^2^ (2.59%); see Supporting Information] had the least consistent effect on discharge rate variability across individual motor units. However, across participants, condition [STR_hold_: σ^2^ = 5.12%^2^ (2.26%), SHO_hold_: σ^2^ = 1.66%^2^ (1.29%)] and the interaction between contraction intensity and STR_hold_ [σ^2^ = 5.65%^2^ (2.38%)] had the least consistent effects on discharge rate variability. Nevertheless, a substantial amount of residual variance within the model remained unexplained [σ^2^ = 12.65%^2^ (3.56%)].

### 3.10 Unique motor units

**FIGURE 5:**
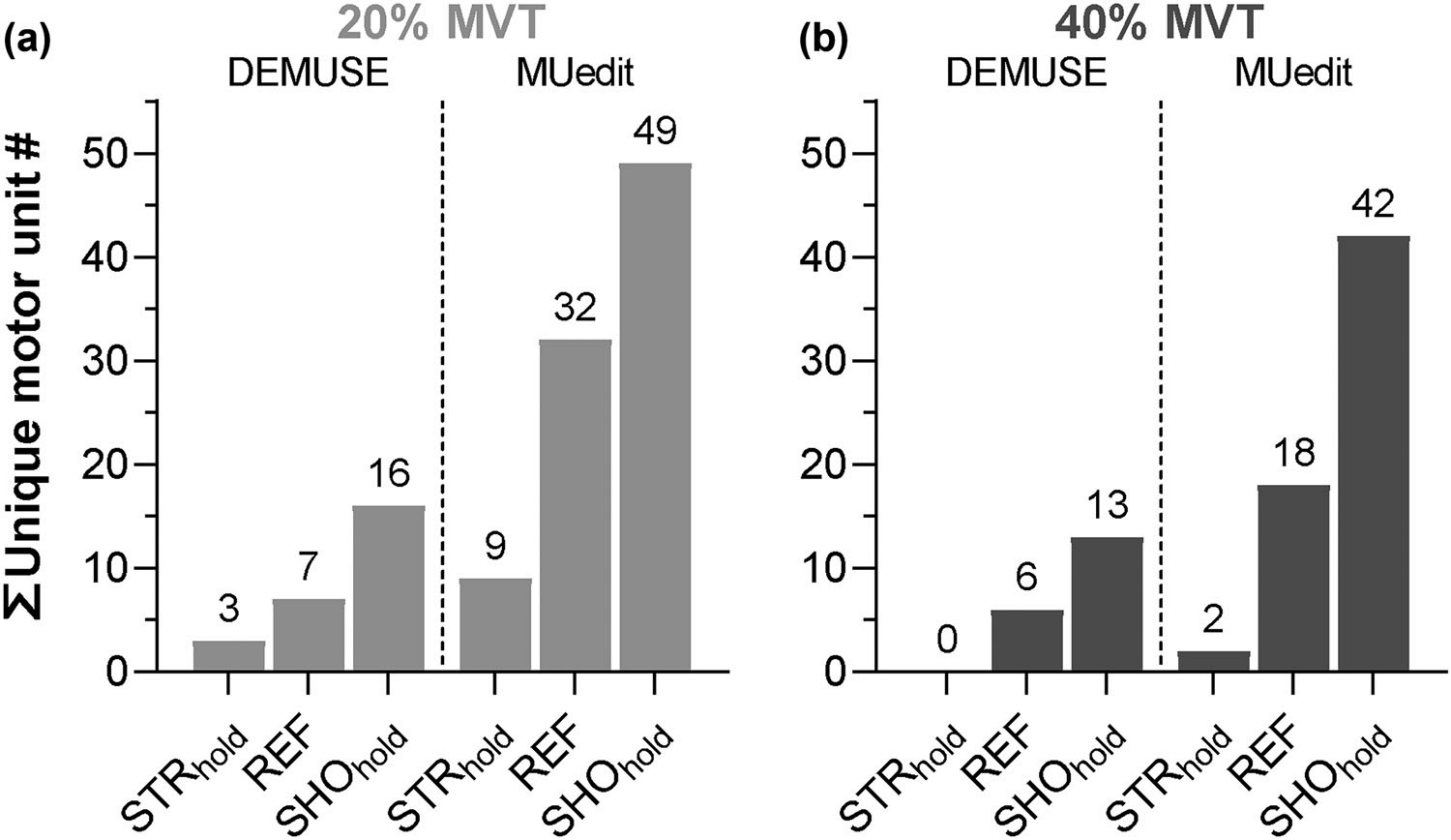
Sum of unique tibialis anterior motor units identified across participants (*n *= 17). Each unique motor unit identified was summed per decomposition approach (DEMUSE or MUedit), contraction intensity [(a) 20% of maximum voluntary torque (MVT); and (b) 40% of MVT] and condition [stretch‐hold (STR_hold_), fixed‐end reference (REF) and shortening‐hold (SHO_hold_) contractions] to show the higher number of unique motor units identified in SHO_hold_ and the lower number identified in STR_hold_ relative to REF.

By design, the number of matched motor units was consistent among conditions at each contraction intensity in the matched motor unit analysis. However, more unique motor units were identified in SHO_hold_ than in REF and STR_hold_ at each contraction intensity, independent of the decomposition algorithm (Figure 5). Additionally, more unique motor units were identified in REF than in STR_hold_ (Figure 5), but variability was high across participants. With DEMUSE at 20% and 40% MVT, only 7 and 4 of 17 participants, respectively, had unique motor units identified exclusively in SHO_hold_ and/or REF. Conversely, with MUedit at 20% and 40% MVT, 12 and 12 of 17 participants, respectively, had unique motor units identified exclusively in SHO_hold_ and/or REF.

## 4 DISCUSSION

The present study was designed to determine the extent of neural drive modulation within the human TA following active muscle stretch or shortening, compared with torque‐ and joint angle‐matched fixed‐end reference contractions. Neural drive differences were assessed using two decomposition approaches by comparing the discharge rates of matched motor units, in addition to the CST‐derived discharge rates of matched and unique motor units among conditions. We found negligible differences in the discharge rate of matched motor units using a conventional versus automatic decomposition approach. However, the automatic versus subjective approach systematically underestimated the CST‐derived discharge rate of matched and unique motor units, which has implications for more in‐depth analysis of motor unit spike trains. As expected, with either decomposition approach, we found that neural drive decreased by similar amounts following active stretch at two contraction intensities (20% and 40% MVT), whereas neural drive increased more following active shortening at 40% MVT, owing to increases in discharge rate that were not apparent at 20% MVT. These results suggest that the neural drive modulation following active shortening, but not active stretch, is contraction‐intensity specific, presumably to account for differences in rFD between contraction intensities (De Ruiter et al., 1998; Raiteri et al., 2024) that might not exist for rFE (Oskouei & Herzog, 2006; Seiberl et al., 2012). We also found indirect evidence of additional motor unit recruitment following active shortening and reduced motor unit recruitment following active stretch based on a respectively higher and lower number of unique motor units relative to the reference condition at both contraction intensities. Against our expectations, we did not find increased torque variability following active stretch contractions, which might be because torque steadiness is not affected by a net decline in neural drive alone. Lastly, we found that EMG‐amplitude‐based muscle force predictions were inaccurate on average by 2%–5% and ≤14% in individual cases following an active muscle length change, owing to changes in the neural drive to the muscle to account for changes in its force capacity.

### 4.1 Neural drive modulations

Following active stretch, we observed fewer unique motor units at 20% and 40% MVT, and similar respective decreases of 9% and 6% in EMG amplitude and 10% and 5% in the CST‐derived discharge rate relative to the reference condition. Such reductions in EMG amplitude fall within the range of 5%–44% from previous in vivo torque‐matched experiments on the TA (Jakobi et al., 2020; Paquin & Power, 2018), soleus (Dalton et al., 2018), superficial quadriceps (Altenburg et al., 2008) and adductor pollicis (Oskouei & Herzog, 2005). Like the present study, these studies (except one; Altenburg et al., 2008) permitted increases in torque during active stretch. If we therefore ignore the study by Altenburg et al. (2008) and compare the other studies with contraction intensities from 20% to 40% MVT, reductions in EMG amplitude ranged from 5% (Dalton et al., 2018) and 12% (Oskouei & Herzog, 2005) to 24% (Jakobi et al., 2020; Paquin & Power, 2018) and 35% (Paquin & Power, 2018). Consequently, the previous studies on TA indicate larger reductions in EMG amplitude following active stretch compared with the present study.

These between‐study differences in EMG amplitude reductions following active stretch might arise for several reasons. Firstly, we used high‐density surface EMG to attain a more representative overview of the activity of the muscle, and by changing the spatial selectivity with conventional bipolar EMG or intramuscular EMG, previous studies might have overestimated the size of the muscle activity reduction. Secondly, we did not exclude ‘non‐responders’ from the statistical analysis, because all participants exhibited reductions in EMG amplitude following stretch in some trials. However, if we excluded trials with no mean EMG amplitude reduction, then the mean EMG amplitude reductions still do not exceed 10%. Thirdly, we also tested at a comparably shorter reference muscle–tendon unit length compared with previous studies. Given that rFE appears to increase with an increase in the final muscle length attained during submaximal voluntary contractions (Bakenecker et al., 2022), it is possible that our shorter reference length (i.e., 20° plantar flexion versus 25° or 40° plantar flexion) led to less rFE than previous studies on the TA (Jakobi et al., 2020; Paquin & Power, 2018) and, correspondingly, attenuated the reduction in EMG amplitude following active stretch. Notably, we did not test at a relatively longer reference length, because we wanted to match the ranges of motion of the stretch and shortening contractions.

Our observed reduction in discharge rate of 10% following active stretch at 20% MVT is also likely to be less than the 27% reduction reported by Jakobi et al. (2020) at 20% MVT for the reasons outlined above (specifically, reasons 1 and 3). Our discharge rate findings are also at odds with findings in vastus lateralis reported by Altenburg et al. (2008), who found non‐significant reductions in discharge rate of 1% following active stretch. Again, this might be because those authors (Altenburg et al., 2008) used intramuscular EMG and tested at a comparably shorter reference length than we did (i.e., on the plateau versus descending limb of the force–length relationship of the muscle), which might have led to less rFE following active stretch. The torque‐matching strategy of Altenburg et al. (2008) was also different, in that participants were instructed to match a constant torque during active muscle–tendon unit stretch. This torque‐matching strategy might have reduced the rFE following active stretch, because rFE and peak fascicle force during stretch are positively correlated, at least within the TA during MVCs {95% CI [0.30 to 0.82]; Tecchio et al., 2024}, and we found 50% higher mean torques during active stretch compared with our fixed‐end reference contractions. Consequently, we speculate that the amount of neural drive modulation following active stretch was likely to be higher in our study because of the higher forces attained during stretch and our relatively longer reference muscle length.

Following active shortening, we observed additional unique motor units at 20% and 40% MVT, and dissimilar respective increases of 7% and 11% in EMG amplitude and 2% and 9% in the CST‐derived discharge rate relative to the reference condition. Such increases in EMG amplitude fall within the range of 9%–48% from previous in vivo torque‐matched experiments on the TA (Paquin & Power, 2018), superficial quadriceps (Altenburg et al., 2008) and adductor pollicis (Rousanoglou et al., 2007). However, the larger increase in neural drive at 40% versus 20% MVT in the present study is at odds with one previous study, which found similar increases in EMG amplitude (9%–25%) and discharge rate (3%–10%) across contraction intensities ranging from 4% to 50% MVT (Altenburg et al., 2008).

This discrepancy in neural drive results most probably arises because the previous study did not investigate different contraction intensities within the same participants (i.e., only a single contraction intensity was studied at ∼20% above the recruitment threshold of the identified motor units, leading to an investigated contraction intensity range across participants of 4%–47% MVT). That study also yielded a lower mean motor unit yield of around four per participant via intramuscular EMG (Altenburg et al., 2008), which limits the generalisability of those findings to the broader active motor unit pool. More recent findings from Paquin & Power (2018) on the TA support those of our study, because those authors reported increasing rFD effect sizes with increasing contraction intensity. However, Paquin & Power (2018) used conventional bipolar surface EMG and thus could not provide data‐driven insights into the neural drive modulation strategies following active shortening. Interestingly, we found that the neural drive modulation strategy differed between contraction intensities, with discharge rate increasing only at 40% MVT.

The lack of discharge rate modulation at 20% MVT and corresponding increase in EMG amplitude following active shortening, and the additional unique motor units detected compared with the reference condition, potentially indicates that additional motor unit recruitment was the primary strategy used by the CNS to overcome the rFD following shortening. A motor unit recruitment strategy might be ideal, because it recruits new muscle fibres that do not have depressed force capacities owing to active shortening, and this strategy also prevents additional active cycling of depressed cross‐bridges within the already‐recruited fibres. However, this statement remains speculative, because we obtained no direct evidence that new motor units were recruited following versus during active shortening.

The neural drive modulation strategy at 20% MVT was clearly different to the combined motor unit recruitment and discharge rate modulation strategy observed following shortening at 40% MVT, where it is unclear how much the additional increase in discharge rate helped to compensate for additional rFD; with additional rFD being likely following higher‐force shortening based on previous findings from the cat soleus (Herzog & Leonard, 2007; Leonard & Herzog, 2005), human adductor pollicis (De Ruiter & De Haan, 2003) and human TA (Raiteri et al., 2024). We speculate that a combined motor unit recruitment and discharge rate modulation strategy to mitigate rFD at higher contraction intensities might not be optimal, but emerges naturally as the pool of non‐active, non‐depressed fibres diminishes, limiting the ability to recruit additional motor units. Consequently, the CNS strategy used to mitigate rFD following active shortening might face unexplored constraints that depend on both neural drive strength and rFD magnitude. However, additional analysis of the motor unit recruitment threshold and coherence analysis of low‐frequency neural drive oscillations are required to confirm this.

In line with our third hypothesis, we found that repeated‐measures changes in TA motor unit discharge rates and EMG amplitudes were strongly and positively correlated at 20% and 40% MVT. However, the correlation was not perfect, which is to be expected, because EMG amplitudes should increase with increases in discharge rate of the same motor units across conditions and with additional motor unit recruitment of unique and unidentified motor units (Merletti & Farina, 2016). Owing to the low and variable number of unique motor units detected (Figure 5), we did not analyse their discharge rates statistically. Based on random chance, we would expect a 33.3% probability of detecting unique motor units in each condition. However, we found respective distributions in STR_hold_, REF and SHO_hold_ of 11%, 31% and 58% at 20% MVT, and 2%, 30% and 68% at 40% MVT. This distribution suggests additional motor unit recruitment in SHO_hold_ relative to REF, alongside reduced recruitment in STR_hold_. Although this pattern varied between participants, these observations indicate that comparing the discharge rates of matched motor units exclusively leads to a loss of information about unique motor units and potential differences in recruitment strategies following active stretch versus shortening.

### 4.2 Torque steadiness

We did not find support for our hypothesis that torque steadiness would be reduced following muscle–tendon unit stretch compared with the fixed‐end reference contractions. The increase in the CV in active torque at 20% MVT following active stretch was only 0.2% higher than in REF, and across all conditions the mean CV did not exceed 2%. These findings are at odds with previous work, which found higher mean CVs in torque of 5%–9% and lower torque steadiness in the steady state following active stretch compared with fixed‐end reference contractions at 10% and 20% MVT (Jakobi et al., 2020).

The discrepancy in torque steadiness results between studies might be explained by recent findings from our group; we observed that large torque differences during a contraction can reduce torque steadiness (Raiteri et al., 2026), and in the previous study (Jakobi et al., 2020), the active torque difference was ∼4‐fold at the start versus end of the STR_hold_ contractions (see their figure 1A), unlike in our study (see our Figure 1a). Additionally, the previously observed mean reduction in torque steadiness following stretch was smaller at 20% MVT (∼1.1%, unreported SD) than at 10% MVT (∼2.6%). This finding is surprising if driven by rFE‐based mechanisms, because rFE appears to be independent of contraction intensity (Oskouei & Herzog, 2006; Seiberl et al., 2012), whereas the reduction in torque steadiness differed significantly across contraction intensities (Jakobi et al., 2020). Nevertheless, given that none of the studies discussed here directly assessed the relationship between rFE magnitude and torque steadiness (i.e., rFE was not quantified), additional EMG amplitude‐matched studies at different contraction intensities and final muscle–tendon unit lengths are needed to confirm whether rFE‐based mechanisms drive reductions in torque steadiness following active muscle stretch.

### 4.3 Sex differences

We did not use null‐hypothesis testing to investigate sex differences in the present study because only a very large effect size of >1.76 (Cohen's *d*_z_) could have been detected with a two‐tailed α‐level of 5% and a desired statistical power of 90% with 6 women and 11 men. With that in mind, there appeared to be three to seven fewer motor units identified per person in women compared with men, which is in line with some previous reports (Jenz et al., 2023; Lulic‐Kuryllo & Inglis, 2022; Taylor et al., 2022), but not others (Inglis & Gabriel, 2020, 2021; Luckey et al., 2026; Yacyshyn et al., 2025). We also observed greater sex differences in the motor unit yield with MUedit than DEMUSE (Table 6), which might help to explain discrepancies between previous studies. The mean TA motor unit discharge rate and discharge rate variability were also slightly higher in women than men (0.9 Hz and 0.5%; Table 7), which is similar to previous reports of higher TA discharge rates (Inglis & Gabriel, 2020; Kowalski & Christie, 2020; Taylor et al., 2022) and CVs in discharge rate (Inglis & Gabriel, 2021; Kowalski & Christie, 2020) in women compared with men at similar contraction intensities. However, it is unclear whether these differences in our study are meaningful, because we also found higher normalised EMG amplitudes in women compared with men (1.8% MVC; Table 7), which might have led to the slightly higher discharge rates and CVs in discharge rate in women. Future studies with between 23 and 86 women and the same number of men are needed to confirm whether there are large to modest (Cohen's *d* ≤1 to ≥0.5) sex differences in motor unit behaviour (α_two‐tailed_ = 5%, β = 10%, two independent groups).

**TABLE 6:** Number of matched motor units identified between sexes for each algorithm at each contraction intensity and combined.

Algorithm	Combined		20% MVT		40% MVT	
	Total MU #		MU #		MU #	
	Women	Men	Women	Men	Women	Men
DEMUSE	148	355	14 (5) [8–20]	18 (5) [12–31]	11 (5) [6–19]	14 (4) [5–22]
MUedit	217	561	20 (11) [8–35]	27 (8) [17–37]	17 (10) [9–35]	24 (8) [8–34]

*Notes*: Values are between‐subject means (SD) [minimum–maximum] except for combined, which is the between‐subject sum; *n *= 6 (women) and *n *= 11 (men).

Abbreviations: MU, motor unit; MVT, maximum voluntary torque.

**TABLE 7:** Sex differences in matched tibialis anterior motor unit discharge rates, discharge rate variability and normalised tibialis anterior EMG amplitudes during the torque‐matched steady state of the six tested conditions.

Subjects	Discharge rate (Hz)		Discharge rate variability (%)		EMG (% MVC)	
	20% MVT	40% MVT	20% MVT	40% MVT	20% MVT	40% MVT
Women	12.6 (1.9)	15.3 (2.0)	14.7 (2.2)	17.3 (2.6)	19.5 (5.9)	31.6 (6.6)
Men	11.7 (1.2)	14.4 (1.3)	14.2 (1.8)	16.8 (2.0)	17.7 (4.3)	29.8 (5.0)

*Note*: Discharge rate and discharge rate variability were averaged from the two decomposition algorithms (DEMUSE and MUedit), and all three variables were averaged among conditions. Values are between‐subject means (SD); *n *= 6 (women) and *n *= 11 (men).

Abbreviations: MVT, maximum voluntary torque; MVC, maximal voluntary contraction.

### 4.4 Limitations

The neural drive changes we observed are likely to be muscle length specific, because rFE and rFD are both affected by muscle length (Bakenecker et al., 2022; Van Noten & Van Leemputte, 2011). Owing to our study design, we were limited to comparing motor unit discharge rates, because each condition started at a different muscle length, preventing us from attaining other important insights, such as differences in motor unit recruitment thresholds. We were also limited to decomposing only the steady state of the contractions owing to current limitations with tracking temporal and spatial changes in the waveforms of the surface‐recorded motor unit action potentials. Furthermore, it is important to note that differences in contraction intensity during the active stretch or shortening phase of our experiments might have influenced the motor unit discharge properties in the following steady‐state phase, independent of changes attributable to active stretch or shortening (i.e., if dynamic torques were matched, the neural drive reduction or increase might be attenuated or intensified following stretch or shortening, respectively). We also limited the number of decomposition iterations to 50 to limit processing time to ∼10 min. In a subset of six random participants, increasing to 250 iterations increased the mean motor unit yield by 2 units (range: −2 to 7 units), but did not materially change discharge rates [mean (SD) difference: −0.2 (0.4) Hz] and increased mean computation time to 6 h (range: 4–8 h). Lastly, we omitted an in‐depth analysis of motor unit spike trains (i.e., intramuscular coherence) because the differences among conditions were algorithm specific (see Supporting Information), thereby limiting the generalisability of the additional physiological insights gained.

Our finding that the discharge rate response to contraction intensity and decomposition algorithm was more variable at the motor unit level than at the participant level, whereas the reverse was true for condition, is probably because we performed separate decompositions at each contraction intensity. We found that grouping the contraction intensities together biased the decomposition to higher‐threshold motor units active at 40% but not 20% MVT, subsequently reducing the motor unit yield at 20% MVT, which is likely to be because decompositions are biased to identify sources with the highest energy (Negro et al., 2016). Our approach to overcome this limitation, by performing contraction intensity‐specific decompositions, probably explains why motor units responded less consistently to contraction intensity than condition; i.e., we tracked different motor units between contraction intensities, but the same motor units across conditions. Consequently, the opposite effects of contraction intensity and condition on the discharge rate variance at the motor‐unit level versus participant level are not surprising and do not compromise our main conclusions about neural drive modulations following active stretch versus shortening.

Although we found a potential order effect among conditions, it is unlikely to affect our main conclusions. Specifically, we did not observe a significantly increased discharge rate following shortening relative to the reference condition at 20% MVT, despite a higher likelihood of fatigue, given that the former condition was most frequently performed last at 20% MVT. Additionally, at 40% MVT, we observed an increased discharge rate following shortening relative to the reference condition, although the influence of fatigue was minimised with the former condition being performed predominantly first at 40% MVT. Following stretch, the wider 95% CI of the mean discharge rate differences relative to the reference condition at 40% MVT might stem from a higher likelihood of fatigue, given that the former condition was less frequently performed first at 40% versus 20% MVT.

### 4.5 Relevance

Our results add to the growing body of evidence that muscle force predictions based on EMG measurements are likely to be inaccurate following dynamic contractions because active muscle stretch and shortening lead to rFE and rFD, which alter the neural drive to a muscle to attain a given force. Although the direction (i.e., overestimation or underestimation) of force error is presumably easy to predict based on the direction of muscle length change, our data show that the magnitude of this error is inconsistent between contraction intensities and participants. Consequently, contraction intensity‐specific and subject‐specific calibration is likely to be required to improve EMG‐based muscle force predictions within musculoskeletal modelling and simulation frameworks. Further work should investigate other muscles and the effect of different preloads, ranges of motion and speeds, and whether changes in neural drive *during* active stretch versus shortening mirror or oppose those *following* active stretch versus shortening.

## 5 CONCLUSION

In conclusion, we found that to account for the enhanced force capacity of muscle following active stretch, neural drive to the TA is reduced to attain a given dorsiflexion torque. Conversely, to account for the depressed force capacity of muscle following active shortening, neural drive to the TA is increased. These findings are based on a robust two‐method decomposition approach, yielding 19 decomposed motor units per person on average, and suggest that the neural drive to a muscle depends on the history‐dependent force capacity of the muscle. Importantly, the way in which neural drive is modulated differs among contraction types, being independent of contraction intensity following active muscle stretch, but being dependent on contraction intensity following active muscle shortening. We speculate that the neural drive strategy of the CNS effectively reduces force deficits following active shortening at a low contraction intensity by recruiting new muscle fibres without depressed force capacities. Conversely, at a relatively higher contraction intensity, this strategy becomes less effective as the pool of non‐active, non‐depressed fibres diminishes, and a combined motor unit recruitment and discharge rate modulation strategy emerges.

## AUTHOR CONTRIBUTIONS

Brent James Raiteri, Karla Friederike Bosse and Daniel Hahn conceived and designed research, Karla Friederike Bosse performed experiments, Brent James Raiteri analysed data, Marta Boccardo manually edited DEMUSE decompositions, Alain Charles Vandal assisted with the statistical analysis and interpretation, Brent James Raiteri prepared figures and drafted the manuscript. All authors interpreted results of experiments, edited and revised the manuscript, approved the final version of the manuscript and agree to be accountable for all aspects of the work in ensuring that questions related to the accuracy or integrity of any part of the work are appropriately investigated and resolved. All persons designated as authors qualify for authorship, and all those who qualify for authorship are listed.

## CONFLICT OF INTEREST

The authors declare no conflicts of interest.

## GENERATIVE AI STATEMENT

A large language model developed by Google was used to improve the structural clarity, language and overall readability of the original manuscript text. All suggestions were independently reviewed, verified and edited as required by the authors, who maintain full accountability for the final content of this work.

## Supporting information



Supporting Information

## Data Availability

Datasets generated and analysed during the present study are available in the following Zenodo repository: https://doi.org/10.5281/zenodo.18922801.
