# Supplementary material for: Stretch versus shortening contractions subsequently decrease versus increase neural drive to the human tibialis anterior
Source: Exp Physiol. 2026 Sep 12;111(10):4793–813. doi: 10.1113/EP094110 (PMC13570787; doi:10.1113/EP094110)
Supplement: Supplementary file 1 [file EPH-111-4793-s001.docx]

SUPPORTING INFORMATION

[Agreement between decomposition approaches	5](#_Toc233988450)

[Rate of agreement	5](#_Toc233988451)

[Limits of agreement	6](#_Toc233988452)

[Decomposition settings	7](#_Toc233988453)

[DEMUSE	7](#_Toc233988454)

[MUedit	8](#_Toc233988455)

[Active torque model fitting and results	9](#_Toc233988456)

[Random-intercept model with no interaction and with/without individual trials nested under participants	9](#_Toc233988457)

[Random-intercept model with interaction	10](#_Toc233988458)

[Model summary	10](#_Toc233988459)

[Model- & sandwich-based standard errors and p-values	11](#_Toc233988460)

[Interaction, intensity & condition p-values	12](#_Toc233988461)

[Post-hoc comparisons	12](#_Toc233988462)

[Random-slope-and-intercept model with interaction	13](#_Toc233988463)

[Model summary	13](#_Toc233988464)

[Model- & sandwich-based standard errors and p-values	14](#_Toc233988465)

[Post-hoc comparisons	15](#_Toc233988466)

[Coefficient of variation in active torque (torque steadiness) model fitting and results	16](#_Toc233988467)

[Random-intercept model with no interaction and with/without individual trials nested under participants	16](#_Toc233988468)

[Random-intercept model with interaction	17](#_Toc233988469)

[Model summary	17](#_Toc233988470)

[Model- & sandwich-based standard errors and p-values	18](#_Toc233988471)

[Interaction, intensity & condition p-values	19](#_Toc233988472)

[Post-hoc comparisons	19](#_Toc233988473)

[Random-slope-and-intercept model with interaction	20](#_Toc233988474)

[Model summary	20](#_Toc233988475)

[Model- & sandwich-based standard errors and p-values	21](#_Toc233988476)

[Post-hoc comparisons	21](#_Toc233988477)

[Normalised high-density EMG amplitude model fitting and results	23](#_Toc233988478)

[Random-intercept model with no interaction and with/without individual trials nested under participants	23](#_Toc233988479)

[Random-intercept model with interaction	24](#_Toc233988480)

[Model summary	24](#_Toc233988481)

[Model- & sandwich-based standard errors and p-values	25](#_Toc233988482)

[Motor unit discharge rate of matched motor units model fitting and results	26](#_Toc233988483)

[Random-intercept model with no interaction and with/without individual motor units nested under participants	26](#_Toc233988484)

[Random-intercept model with interaction	27](#_Toc233988485)

[Model summary	27](#_Toc233988486)

[Model- & sandwich-based standard errors and p-values	28](#_Toc233988487)

[Interaction, intensity & condition p-values	29](#_Toc233988488)

[Post-hoc comparisons	29](#_Toc233988489)

[Random-slope-and-intercept model with interaction	30](#_Toc233988490)

[Model summary	30](#_Toc233988491)

[Model- & sandwich-based standard errors and p-values	32](#_Toc233988492)

[Interaction, intensity & condition p-values	33](#_Toc233988493)

[Post-hoc comparisons	33](#_Toc233988494)

[Coefficient of variation in motor unit discharge rate model fitting and results	35](#_Toc233988495)

[Random-intercept model with no interaction and with/without individual motor units nested under participants	35](#_Toc233988496)

[Random-intercept model with interaction	36](#_Toc233988497)

[Model summary	36](#_Toc233988498)

[Model- & sandwich-based standard errors and p-values	37](#_Toc233988499)

[Interaction, intensity & condition p-values	38](#_Toc233988500)

[Post-hoc comparisons	38](#_Toc233988501)

[Random-slope-and-intercept model with interaction	39](#_Toc233988502)

[Model summary	39](#_Toc233988503)

[Model- & sandwich-based standard errors and p-values	41](#_Toc233988504)

[Interaction, intensity & condition p-values	42](#_Toc233988505)

[Post-hoc comparisons	42](#_Toc233988506)

[Motor unit discharge rate of matched and unique motor units based on the smoothed cumulative spike train model fitting and results	44](#_Toc233988507)

[Random-intercept model with no interaction	44](#_Toc233988508)

[Random-intercept model with interaction	45](#_Toc233988509)

[Model summary	45](#_Toc233988510)

[Model- & sandwich-based standard errors and p-values	46](#_Toc233988511)

[Interaction, intensity & condition p-values	46](#_Toc233988512)

[Post-hoc comparisons	47](#_Toc233988513)

[Random-slope-and-intercept model with interaction	48](#_Toc233988514)

[Model summary	48](#_Toc233988515)

[Model- & sandwich-based standard errors and p-values	50](#_Toc233988516)

[Interaction, intensity & condition p-values	50](#_Toc233988517)

[Post-hoc comparisons	50](#_Toc233988518)

[Active torque estimation from EMG amplitude in the REF condition model fitting and results	53](#_Toc233988519)

[Random-intercept model with no interaction and without individual trials nested under participants	53](#_Toc233988520)

[Model summary	53](#_Toc233988521)

[Model- & sandwich-based standard errors and p-values	54](#_Toc233988522)

[Intramuscular coherence analysis: Area under the curve within the delta band model fitting and results for algorithms combined	56](#_Toc233988523)

[Random-intercept model with no interaction	56](#_Toc233988524)

[Random-intercept model with interaction	57](#_Toc233988525)

[Model summary	57](#_Toc233988526)

[Model- & sandwich-based standard errors and p-values	58](#_Toc233988527)

[Interaction, intensity & condition p-values	58](#_Toc233988528)

[Post-hoc comparisons	59](#_Toc233988529)

[Random-slope-and-intercept model with interaction	60](#_Toc233988530)

[Intramuscular coherence analysis: Area under the curve within the delta band following the manual DEMUSE approach	61](#_Toc233988531)

[Random-intercept model with no interaction	61](#_Toc233988532)

[Random-intercept model with interaction	62](#_Toc233988533)

[Model summary	62](#_Toc233988534)

[Model- & sandwich-based standard errors and p-values	63](#_Toc233988535)

[Interaction, intensity & condition p-values	63](#_Toc233988536)

[Post-hoc comparisons	64](#_Toc233988537)

[Intramuscular coherence analysis: Area under the curve within the delta band following the automatic MUedit approach	65](#_Toc233988538)

[Random-intercept model with no interaction	65](#_Toc233988539)

[Random-intercept model with interaction	66](#_Toc233988540)

[Model summary	66](#_Toc233988541)

[Model- & sandwich-based standard errors and p-values	67](#_Toc233988542)

[Interaction, intensity & condition p-values	67](#_Toc233988543)

[Post-hoc comparisons	68](#_Toc233988544)

[References	69](#_Toc233988545)

## Agreement between decomposition approaches

### Rate of agreement

For each motor unit shared between DEMUSE and MUedit at one contraction intensity, the rate of agreement (RoA) between approaches (i.e. manual editing for DEMUSE decompositions and automatic editing for MUedit decompositions) was calculated as described in Hug et al. (2021). A maximal lag of 50 ms was allowed between motor unit discharge patterns to find the best overlap, and a ±1 time point discharge time tolerance was used (i.e. discharge times were considered to agree if they did not differ by more than 0.5 ms).

A total of 456 RoA values were calculated (i.e. 2 contraction intensities × 5-31 common motor units). RoA decreased slightly with contraction intensity, with mean (standard deviation) values of 81.7 (2.7)% and 80.1 (1.9)% at 20 and 40% MVT, respectively. RoA ranged from 43.7 to 97.2%, with 294 out of 456 values (64.5%) being lower than 85%. These RoA values are low compared with previous research (Hug *et al.*, 2021), indicating relatively poor agreement in discharge rate timings between the two approaches used in this study. Notably though, the RoA between the automatic editing approach and the manual editing approach in DEMUSE was higher than that between the automatic editing approach and the unedited DEMUSE decompositions (20% MVT: 75.3 (4.2)%; 40% MVT: 74.9 (3.6)%; Fig. S1). In line with previous research (Murks *et al.*, 2025), the number of common motor units also decreased with increasing contraction level (15 (6) and 12 (5) at 20 and 40% MVT, respectively) and at least 2 (1) common motor units were found across contraction intensities with one decomposition algorithm.

**Figure S1.** Frequency distributions of the rate of agreement values for shared motor units between (A) manually edited DEMUSE decompositions and automatically edited MUedit decompositions and (B) unedited DEMUSE decompositions and automatically edited MUedit decompositions.

### Limits of agreement

Limits of agreement between the mean discharge rates in each condition and at each contraction intensity between decomposition approaches was assessed via repeated-measures Bland-Altman analysis (note that the code was modified to fit linear mixed-effects models and to use Satterthwaite’s approximation to compute the denominator degrees of freedom for the *t*-statistics of the fixed-effects coefficients; Wade *et al.*, 2023).

**Figure S2.** Agreement in mean discharge rates between manual editing of DEMUSE decompositions and automatic editing of MUedit decompositions at (A) 20% of maximum voluntary torque (MVT) and (B) 40% MVT based on repeated-measures Bland-Altman analysis. The agreement was high, with negligible bias and relatively narrow 95% limits of agreement (LOA; see text below for numbers).

There was negligible systematic bias at 20 (0.02 (0.30) Hz) and 40% MVT (0.18 (0.42) Hz) between decomposition approaches, with random error being more prominent than systematic bias. The 95% limits of agreement were also narrow for both contraction intensities, but narrower at 20 (-0.62 to 0.65 Hz) than 40% MVT (-0.72 to 1.07 Hz; Fig. S2). These findings indicate high agreement in mean discharge rates between the two decomposition approaches used in this study. Consequently, due to the relatively minor differences between the manual and automatic editing approaches, the automatic editing approach may be favoured for calculating mean discharge rate during steady-state torque production (following visual verification of the motor unit pulse trains) due to its reduced subjectivity and time cost. Importantly though, the use of an automatic editing approach for assessing metrics requiring accurate discharge rate timings and for calculating mean discharge rates when force varies, remains questionable. In such cases, manual editing based on strict, well-defined rules incorporating muscle physiology would be preferable (e.g., Del Vecchio *et al.*, 2020; Hug *et al.*, 2021).

## Decomposition settings

### DEMUSE

**Figure S3.** Screenshot of the [DEMUSE](https://demuse.feri.um.si/) (v5.01; Holobar & Zazula, 2007*a*, 2007*b*) decomposition settings used in MATLAB, with a default decomposition speed of “slower and accurate”. Other decomposition settings included: Smart PT orthogonalization = on; Sparse decomposition = off; Minimal number of motor unit firings = 10; Maximal coefficient of variation of motor unit inter-spike interval = 1.5; Pulse height = 1.2. DEMUSE filtered the high-density surface electromyography signals with a bandpass filter between 20 and 500 Hz using a dual-pass fourth-order Butterworth filter. The “diff mode” option was selected to enhance local signal components and suppress common-mode noise by converting monopolar signals into adjacent-row (longitudinal) single-differential signals. 5% of the high-density surface electromyography channels (i.e. 3 channels) were then removed based on the lowest ranking correlation coefficients between each channel and its immediate neighbours, which is a standard preprocessing step in DEMUSE before decomposition. Following decomposition, the motor unit discharge patterns were manually edited according to previous guidelines (Del Vecchio *et al.*, 2020; Hug *et al.*, 2021) and duplicate motor units were removed if two motor units shared more than 30% of their identified discharge times (within a time interval of 0.5 ms), with the lower pulse-to-noise ratio motor unit being excluded (Holobar *et al.*, 2010).

### MUedit

**Figure S4.** Screenshot of the [MUedit](https://github.com/simonavrillon/MUedit) (v1.2; Avrillon *et al.*, 2024) decomposition settings used in MATLAB. By default, MUedit filters the high-density surface electromyography signals with an adaptive notch filter to remove frequencies with abnormal peaks, as well as a bandpass filter between 20 and 500 Hz using a dual-pass second-order Butterworth filter. A “random” initialisation was used based on the description in Avrillon et al. (2024) that random weights with the function “skew” resulted in slightly better performance than with an initialization with the maximal EMG values (“EMG max”). Following decomposition with MUedit, duplicate motor units were removed if there were more than 30% of shared discharge times (within a time interval of 0.5 ms) between two motor units, with the lower pulse-to-noise ratio motor unit being excluded (Holobar *et al.*, 2010). Subsequently, the motor unit discharge patterns were automatically edited (see the “Decomposition editing” section within the manuscript) and visually verified.

## Active torque model fitting and results

Fixed effects were fitted using a linear mixed-effects model. Model-based standard errors and p-values are presented. Sandwich variance estimation-based standard errors and p-values were also produced; these do not assume normality of the data. A Wald statistic based on the sandwich variance estimate was also produced to test the effect of factor condition.

### Random-intercept model with no interaction and with/without individual trials nested under participants

This model fits the factors intensity and condition with random intercepts and fixed slopes. We first compared fitting the model by REML vs. ML, and ML resulted in a better fit without nesting individual trials under participants:

```
## $statistics
##                         aic     bic bayes.factor
## no_interaction_REML 894.617 916.698        0.046
## no_interaction_ML   888.461 910.542       21.718
##
## $predicted_differences
##    0%   25%   50%   75%  100%
## 0.000 0.001 0.001 0.001 0.001
## $statistics
##                             aic     bic bayes.factor
## no_interaction_ML       888.461 910.542       17.117
## no_interaction_ML_trial 890.461 916.222        0.058
##
## $predicted_differences
##   0%  25%  50%  75% 100%
##    0    0    0    0    0
```

### Random-intercept model with interaction

This model fits the factors intensity, condition, and the intensity x condition interaction with random intercepts and fixed slopes. The model with an intensity:condition interaction did not result in a better fit:

```
## $statistics
##                       aic     bic bayes.factor
## no_interaction_ML 888.461 910.542      278.323
## interaction_ML    892.358 921.799        0.004
##
## $predicted_differences
##    0%   25%   50%   75%  100%
## 0.003 0.008 0.016 0.024 0.031
```

#### Model summary

```
## Linear mixed model fit by maximum likelihood . t-tests use Satterthwaite's
##   method [lmerModLmerTest]
## Formula: outcome ~ intensity * condition + (1 | pid)
##    Data: d
##
##      AIC      BIC   logLik deviance df.resid
##    892.4    921.8   -438.2    876.4      285
##
## Scaled residuals:
##     Min      1Q  Median      3Q     Max
## -2.0099 -0.7015  0.0710  0.7121  1.7738
##
## Random effects:
##  Groups   Name        Variance Std.Dev.
##  pid      (Intercept) 7.4029   2.7208
##  Residual             0.8727   0.9342
## Number of obs: 293, groups:  pid, 17
##
```

```
## Fixed effects:
##                        Estimate Std. Error        df t value Pr(>|t|)
## (Intercept)             8.14265    0.67364  18.21694  12.088 3.87e-10 ***
## intensity2              8.08558    0.19578 276.04174  41.298  < 2e-16 ***
## condition2             -0.12164    0.18898 276.05444  -0.644    0.520
## condition3              0.06329    0.19298 276.05836   0.328    0.743
## intensity2:condition2  -0.06503    0.27006 276.04584  -0.241    0.810
## intensity2:condition3  -0.08346    0.27230 276.04858  -0.307    0.759
## ---
## Signif. codes:  0 '***' 0.001 '**' 0.01 '*' 0.05 '.' 0.1 ' ' 1
##
## Correlation of Fixed Effects:
##             (Intr) intns2 cndtn2 cndtn3 int2:2
## intensity2  -0.139
## condition2  -0.145  0.497
## condition3  -0.141  0.488  0.508
## intnsty2:c2  0.101 -0.726 -0.699 -0.356
## intnsty2:c3  0.100 -0.720 -0.359 -0.708  0.524
```

#### Model- & sandwich-based standard errors and p-values

```
## Model- & sandwich-based standard errors and p-values
## Click on the arrow for sandwich-based p-values.
## # A tibble: 6 × 6
##   term                  estimate `Model-based s.e.` `Model-based p-value`
##   <chr>                    <dbl>              <dbl>                 <dbl>
## 1 (Intercept)             8.14                0.674             1.96e- 27
## 2 intensity2              8.09                0.196             2.63e-122
## 3 condition2             -0.122               0.189             5.20e-  1
## 4 condition3              0.0633              0.193             7.43e-  1
## 5 intensity2:condition2  -0.0650              0.270             8.10e-  1
## 6 intensity2:condition3  -0.0835              0.272             7.59e-  1
##   `Sandwich s.e.` `Sandwich p-value`
##             <dbl>              <dbl>
## 1           0.448           4.10e-12
## 2           0.464           1.22e-11
## 3           0.113           2.99e- 1
## 4           0.107           5.63e- 1
## 5           0.138           6.43e- 1
## 6           0.139           5.57e- 1
```

#### Interaction, intensity & condition p-values

The Wald statistics testing for an effect of the intensity:condition interaction term, for an effect of the intensity term and the intensity:condition interaction term, and for an effect of the condition term and the intensity:condition interaction term are:

```
##  test Fstat df_num df_denom  p_val sig
##   HTZ  0.17      2     14.7 0.8451
##  test Fstat df_num df_denom   p_val sig
##   HTZ   105      3     13.7 < 1e-04 ***
##  test Fstat df_num df_denom   p_val sig
##   HTZ  4.39      4     12.7 0.01884   *
```

#### Post-hoc comparisons

Estimated marginal means (EMMs) for the interaction between intensity and condition for the random-intercept model, while explicitly using cluster-robust standard errors (which accounts for non-independent data and heteroskedasticity), are reported below. This approach was used to ensure that the confidence intervals and subsequent p-values for the EMMs are more accurate and reliable.

```
##  intensity condition emmean    SE   df lower.CL upper.CL
##  1         1           8.14 0.448 18.2     7.20     9.08
##  2         1          16.23 0.899 18.4    14.34    18.11
##  1         2           8.02 0.464 18.1     7.05     8.99
##  2         2          16.04 0.903 18.1    14.15    17.94
##  1         3           8.21 0.460 18.3     7.24     9.17
##  2         3          16.21 0.940 18.1    14.23    18.18
##
## Degrees-of-freedom method: satterthwaite
## Confidence level used: 0.95
```

\## condition = 1:
\## contrast	estimate	SE df t.ratio p.value
\## intensity1 - intensity2	-8.09 0.464 276 -17.423 <.0001
\##
\## condition = 2:
\## contrast	estimate	SE df t.ratio p.value
\## intensity1 - intensity2	-8.02 0.452 276 -17.736 <.0001
\##
\## condition = 3:
\## contrast	estimate	SE df t.ratio p.value
\## intensity1 - intensity2	-8.00 0.493 276 -16.245 <.0001
\##
\## Degrees-of-freedom method: satterthwaite

### Random-slope-and-intercept model with interaction

This model fits the factors intensity, condition, and the intensity x condition interaction with random intercepts and random slopes. The random-slope-and-intercept model resulted in a better fit than the random-intercept model:

```
## $statistics
##                                 aic     bic  bayes.factor
## interaction_ML              892.358 921.799  0.000000e+00
## interaction_ML_randomSlope -178.358 -75.313 3.312791e+216
##
## $predicted_differences
##    0%   25%   50%   75%  100%
## 0.020 0.474 0.635 1.241 1.778
```

#### Model summary

```
## Linear mixed model fit by maximum likelihood . t-tests use Satterthwaite's
##   method [lmerModLmerTest]
## Formula: outcome ~ intensity * condition + (intensity * condition | pid)
##    Data: d
##
##      AIC      BIC   logLik deviance df.resid
##   -178.4    -75.3    117.2   -234.4      265
##
## Scaled residuals:
##     Min      1Q  Median      3Q     Max
## -3.2171 -0.5326  0.0315  0.4660  4.5874
##
## Random effects:
##  Groups   Name                  Variance Std.Dev. Corr
##  pid      (Intercept)           3.42168  1.8498
##           intensity2            2.82285  1.6801    0.98
##           condition2            0.06300  0.2510   -0.35 -0.22
##           condition3            0.04891  0.2212   -0.22 -0.05  0.94
##           intensity2:condition2 0.07018  0.2649    0.60  0.46 -0.83 -0.83
##           intensity2:condition3 0.07315  0.2705    0.47  0.32 -0.91 -0.91  0.98
##  Residual                       0.01051  0.1025
## Number of obs: 293, groups:  pid, 17
##
```

```
## Fixed effects:
##                        Estimate Std. Error        df t value Pr(>|t|)
## (Intercept)            8.100268   0.448892 16.999783  18.045 1.60e-12 ***
## intensity2             8.060598   0.408076 17.004588  19.753 3.65e-13 ***
## condition2            -0.082972   0.064394 17.140824  -1.289    0.215
## condition3             0.065322   0.057760 16.912796   1.131    0.274
## intensity2:condition2 -0.005281   0.070928 17.145902  -0.074    0.942
## intensity2:condition3 -0.034255   0.072247 17.069114  -0.474    0.641
## ---
## Signif. codes:  0 '***' 0.001 '**' 0.01 '*' 0.05 '.' 0.1 ' ' 1
##
## Correlation of Fixed Effects:
##             (Intr) intns2 cndtn2 cndtn3 int2:2
## intensity2   0.974
## condition2  -0.335 -0.195
## condition3  -0.214 -0.034  0.887
## intnsty2:c2  0.548  0.400 -0.808 -0.753
## intnsty2:c3  0.436  0.276 -0.834 -0.874  0.895
## optimizer (nloptwrap) convergence code: 0 (OK)
## boundary (singular) fit: see help('isSingular')
```

#### Model- & sandwich-based standard errors and p-values

```
## Model- & sandwich-based standard errors and p-values
## Click on the arrow for sandwich-based p-values.
## # A tibble: 6 × 6
##   term                  estimate `Model-based s.e.` `Model-based p-value`
##   <chr>                    <dbl>              <dbl>                 <dbl>
## 1 (Intercept)            8.10                0.449               5.00e-48
## 2 intensity2             8.06                0.408               5.18e-54
## 3 condition2            -0.0830              0.0644              1.99e- 1
## 4 condition3             0.0653              0.0578              2.59e- 1
## 5 intensity2:condition2 -0.00528             0.0709              9.41e- 1
## 6 intensity2:condition3 -0.0343              0.0722              6.36e- 1
##   `Sandwich s.e.` `Sandwich p-value`
##             <dbl>              <dbl>
## 1          0.463            7.37e-12
## 2          0.421            1.85e-12
## 3          0.0661           2.28e- 1
## 4          0.0593           2.87e- 1
## 5          0.0726           9.43e- 1
## 6          0.0741           6.50e- 1
```

#### Post-hoc comparisons

Estimated marginal means (EMMs) for the interaction between intensity and condition for the random-slope-and-intercept model, while explicitly using cluster-robust standard errors (which accounts for non-independent data and heteroskedasticity), are reported below. This approach was used to ensure that the confidence intervals and subsequent p-values for the EMMs are more accurate and reliable.

```
##  intensity condition emmean    SE df lower.CL upper.CL
##  1         1           8.10 0.463 17     7.12     9.08
##  2         1          16.16 0.877 17    14.31    18.01
##  1         2           8.02 0.445 17     7.08     8.96
##  2         2          16.07 0.896 17    14.18    17.96
##  1         3           8.17 0.454 17     7.21     9.12
##  2         3          16.19 0.897 17    14.30    18.09
##
## Degrees-of-freedom method: satterthwaite
## Confidence level used: 0.95
```

\## condition = 1:
\## contrast	estimate	SE df t.ratio p.value
\## intensity1 - intensity2	-8.06 0.421 17 -19.164 <.0001
\##
\## condition = 2:
\## contrast	estimate	SE df t.ratio p.value
\## intensity1 - intensity2	-8.06 0.455 17 -17.714 <.0001
\##
\## condition = 3:
\## contrast	estimate	SE df t.ratio p.value
\## intensity1 - intensity2	-8.03 0.447 17 -17.962 <.0001
\##
\## Degrees-of-freedom method: satterthwaite

## Coefficient of variation in active torque (torque steadiness) model fitting and results

Fixed effects were fitted using a linear mixed-effects model. Model-based standard errors and p-values are presented. Sandwich variance estimation-based standard errors and p-values were also produced; these do not assume normality of the data. A Wald statistic based on the sandwich variance estimate was also produced to test the effect of factor condition.

### Random-intercept model with no interaction and with/without individual trials nested under participants

This model fits the factors intensity and condition with random intercepts and fixed slopes. We first compared fitting the model by REML vs. ML, and ML resulted in a better fit´without individual trials nested under participants:

```
## $statistics
##                         aic     bic bayes.factor
## no_interaction_REML 460.823 482.904        0.001
## no_interaction_ML   446.371 468.452     1374.160
##
## $predicted_differences
##    0%   25%   50%   75%  100%
## 0.000 0.000 0.001 0.002 0.005
```

```
## $statistics
##                             aic     bic bayes.factor
## no_interaction_ML       446.371 468.452       17.117
## no_interaction_ML_trial 448.371 474.133        0.058
##
## $predicted_differences
##   0%  25%  50%  75% 100%
##    0    0    0    0    0
```

### Random-intercept model with interaction

This model fits the factors intensity, condition, and the intensity x condition interaction with random intercepts and fixed slopes. The model with an intensity:condition interaction did not result in a better fit:

```
## $statistics
##                       aic     bic bayes.factor
## no_interaction_ML 446.371 468.452      133.201
## interaction_ML    448.795 478.236        0.008
##
## $predicted_differences
##    0%   25%   50%   75%  100%
## 0.014 0.022 0.032 0.045 0.052
```

#### Model summary

```
## Linear mixed model fit by maximum likelihood . t-tests use Satterthwaite's
##   method [lmerModLmerTest]
## Formula: outcome ~ intensity * condition + (1 | pid)
##    Data: d
##
##      AIC      BIC   logLik deviance df.resid
##    448.8    478.2   -216.4    432.8      285
##
## Scaled residuals:
##     Min      1Q  Median      3Q     Max
## -2.4258 -0.6477 -0.0309  0.5837  3.1038
##
## Random effects:
##  Groups   Name        Variance Std.Dev.
##  pid      (Intercept) 0.09033  0.3005
##  Residual             0.22764  0.4771
## Number of obs: 293, groups:  pid, 17
##
```

```
## Fixed effects:
##                        Estimate Std. Error        df t value Pr(>|t|)
## (Intercept)             1.63325    0.10044  44.88256  16.260   <2e-16 ***
## intensity2             -0.05857    0.09994 276.43851  -0.586   0.5584
## condition2              0.16331    0.09645 276.67838   1.693   0.0915 .
## condition3              0.10219    0.09849 276.75716   1.038   0.3004
## intensity2:condition2  -0.15953    0.13785 276.51876  -1.157   0.2482
## intensity2:condition3  -0.02603    0.13899 276.57436  -0.187   0.8516
## ---
## Signif. codes:  0 '***' 0.001 '**' 0.01 '*' 0.05 '.' 0.1 ' ' 1
##
## Correlation of Fixed Effects:
##             (Intr) intns2 cndtn2 cndtn3 int2:2
## intensity2  -0.476
## condition2  -0.495  0.497
## condition3  -0.484  0.488  0.507
## intnsty2:c2  0.346 -0.726 -0.699 -0.356
## intnsty2:c3  0.343 -0.720 -0.359 -0.708  0.524
```

#### Model- & sandwich-based standard errors and p-values

```
## Model- & sandwich-based standard errors and p-values
## Click on the arrow for sandwich-based p-values.
```

\## # A tibble: 6 × 6
\##	term	estimate `Model-based s.e.` `Model-based p-value`
\##	<chr>	<dbl>	<dbl>	<dbl>
\## 1 (Intercept)	1.63	0.100	1.63e-42
\## 2 intensity2	-0.0586	0.0999	5.58e- 1
\## 3 condition2	0.163	0.0965	9.15e- 2
\## 4 condition3	0.102	0.0985	3.00e- 1
\## 5 intensity2:condition2 -0.160	0.138	2.48e- 1
\## 6 intensity2:condition3 -0.0260	0.139	8.52e- 1
\##	`Sandwich s.e.` `Sandwich p-value`
\##	<dbl>	<dbl>
\## 1	0.133	0.00000000171
\## 2	0.155	0.711
\## 3	0.0680	0.0290
\## 4	0.0794	0.217
\## 5	0.0806	0.0655
\## 6	0.0918	0.780

#### Interaction, intensity & condition p-values

The Wald statistics testing for an effect of the intensity:condition interaction term, for an effect of the intensity term and the intensity:condition interaction term, and for an effect of the condition term and the intensity:condition interaction term are:

```
##  test Fstat df_num df_denom  p_val sig
##   HTZ  2.05      2     14.7 0.1637
##  test Fstat df_num df_denom  p_val sig
##   HTZ  1.41      3     13.7 0.2829
##  test Fstat df_num df_denom  p_val sig
##   HTZ  1.55      4     12.7 0.2473
```

#### Post-hoc comparisons

Estimated marginal means (EMMs) for the interaction between intensity and condition for the random-intercept model, while explicitly using cluster-robust standard errors (which accounts for non-independent data and heteroskedasticity), are reported below. This approach was used to ensure that the confidence intervals and subsequent p-values for the EMMs are more accurate and reliable.

```
##  intensity condition emmean     SE   df lower.CL upper.CL
##  1         1           1.63 0.1330 44.9     1.37     1.90
##  2         1           1.57 0.0869 48.5     1.40     1.75
##  1         2           1.80 0.1290 42.5     1.54     2.06
##  2         2           1.58 0.1020 42.6     1.37     1.78
##  1         3           1.74 0.1440 45.9     1.45     2.03
##  2         3           1.65 0.0898 42.0     1.47     1.83
##
## Degrees-of-freedom method: satterthwaite
## Confidence level used: 0.95
```

\## intensity = 1:
\## contrast	estimate	SE df t.ratio p.value
\## condition1 - condition2 -0.16331 0.0680 277 -2.401 0.0510
\## condition1 - condition3 -0.10219 0.0794 277 -1.286 0.3989
\## condition2 - condition3 0.06113 0.0787 276	0.777 0.4377
\##
\## intensity = 2:
\## contrast	estimate	SE df t.ratio p.value
\## condition1 - condition2 -0.00379 0.0593 276 -0.064 0.9491
\## condition1 - condition3 -0.07616 0.0624 276 -1.221 0.6691
\## condition2 - condition3 -0.07237 0.0654 276 -1.107 0.6691
\##
\## Degrees-of-freedom method: satterthwaite
\## P value adjustment: holm method for 3 tests

### Random-slope-and-intercept model with interaction

This model fits the factors intensity, condition, and the intensity x condition interaction with random intercepts and random slopes. The random-slope-and-intercept model did not result in a better fit than the random-intercept model:

```
## $statistics
##                                aic     bic bayes.factor
## interaction_ML             448.795 478.236      1673516
## interaction_ML_randomSlope 403.852 506.897            0
##
## $predicted_differences
##    0%   25%   50%   75%  100%
## 0.001 0.087 0.191 0.298 0.619
```

#### Model summary

```
## Linear mixed model fit by maximum likelihood . t-tests use Satterthwaite's
##   method [lmerModLmerTest]
## Formula: outcome ~ intensity * condition + (intensity * condition | pid)
##    Data: d
##
##      AIC      BIC   logLik deviance df.resid
##    403.9    506.9   -173.9    347.9      265
##
## Scaled residuals:
##     Min      1Q  Median      3Q     Max
## -2.5352 -0.6171 -0.1403  0.6235  3.7938
##
## Random effects:
##  Groups   Name                  Variance Std.Dev. Corr
##  pid      (Intercept)           0.229146 0.47869
##           intensity2            0.324478 0.56963  -0.82
##           condition2            0.002697 0.05193  -0.12  0.56
##           condition3            0.007567 0.08699   0.39 -0.29  0.52
##           intensity2:condition2 0.001894 0.04352   0.35 -0.69 -0.97 -0.46
##           intensity2:condition3 0.006037 0.07770   0.05 -0.38 -0.94 -0.75  0.93
##  Residual                       0.146239 0.38241
## Number of obs: 293, groups:  pid, 17
##
## Fixed effects:
##                        Estimate Std. Error        df t value Pr(>|t|)
## (Intercept)             1.64034    0.12874  17.30959  12.742 3.17e-10 ***
## intensity2             -0.06999    0.15985  17.24948  -0.438    0.667
## condition2              0.16117    0.07877 118.73122   2.046    0.043 *
## condition3              0.10329    0.08231  19.30419   1.255    0.224
## intensity2:condition2  -0.16014    0.11159 193.86557  -1.435    0.153
## intensity2:condition3  -0.04953    0.11358  76.01681  -0.436    0.664
## ---
## Signif. codes:  0 '***' 0.001 '**' 0.01 '*' 0.05 '.' 0.1 ' ' 1
##
## Correlation of Fixed Effects:
##             (Intr) intns2 cndtn2 cndtn3 int2:2
## intensity2  -0.790
## condition2  -0.325  0.325
## condition3  -0.203  0.171  0.508
## intnsty2:c2  0.247 -0.420 -0.702 -0.355
## intnsty2:c3  0.220 -0.410 -0.378 -0.709  0.530
## optimizer (nloptwrap) convergence code: 0 (OK)
## boundary (singular) fit: see help('isSingular')
```

#### Model- & sandwich-based standard errors and p-values

```
## Model- & sandwich-based standard errors and p-values
## Click on the arrow for sandwich-based p-values.
## # A tibble: 6 × 6
##   term                  estimate `Model-based s.e.` `Model-based p-value`
##   <chr>                    <dbl>              <dbl>                 <dbl>
## 1 (Intercept)             1.64               0.129               2.51e-29
## 2 intensity2             -0.0700             0.160               6.62e- 1
## 3 condition2              0.161              0.0788              4.17e- 2
## 4 condition3              0.103              0.0823              2.11e- 1
## 5 intensity2:condition2  -0.160              0.112               1.52e- 1
## 6 intensity2:condition3  -0.0495             0.114               6.63e- 1
##   `Sandwich s.e.` `Sandwich p-value`
##             <dbl>              <dbl>
## 1          0.131       0.00000000119
## 2          0.159       0.666
## 3          0.0670      0.0287
## 4          0.0843      0.238
## 5          0.0795      0.0612
## 6          0.104       0.639
```

#### Post-hoc comparisons

Estimated marginal means (EMMs) for the interaction between intensity and condition for the random-slope-and-intercept model, while explicitly using cluster-robust standard errors (which accounts for non-independent data and heteroskedasticity), are reported below. This approach was used to ensure that the confidence intervals and subsequent p-values for the EMMs are more accurate and reliable.

```
##  intensity condition emmean     SE   df lower.CL upper.CL
##  1         1           1.64 0.1310 17.3     1.36     1.92
##  2         1           1.57 0.0950 17.0     1.37     1.77
##  1         2           1.80 0.1280 17.1     1.53     2.07
##  2         2           1.57 0.0963 17.9     1.37     1.77
##  1         3           1.74 0.1420 17.3     1.44     2.04
##  2         3           1.62 0.0878 17.3     1.44     1.81
##
## Degrees-of-freedom method: satterthwaite
## Confidence level used: 0.95
```

\## intensity = 1:
\## contrast	estimate	SE	df t.ratio p.value
\## condition1 - condition2 -0.16117 0.0670 118.7 -2.406 0.0531
\## condition1 - condition3 -0.10329 0.0843 19.3 -1.226 0.4700
\## condition2 - condition3 0.05788 0.0723 53.4	0.800 0.4700
\##
\## intensity = 2:
\## contrast	estimate	SE	df t.ratio p.value
\## condition1 - condition2 -0.00103 0.0537 229.9 -0.019 1.0000
\## condition1 - condition3 -0.05376 0.0681 120.4 -0.790 1.0000
\## condition2 - condition3 -0.05272 0.0648 117.9 -0.813 1.0000
\##
\## Degrees-of-freedom method: satterthwaite
\## P value adjustment: holm method for 3 tests

## Normalised high-density EMG amplitude model fitting and results

Fixed effects were fitted using a linear mixed-effects model. Model-based standard errors and p-values are presented. Sandwich variance estimation-based standard errors and p-values were also produced; these do not assume normality of the data. A Wald statistic based on the sandwich variance estimate was also produced to test the effect of factor condition.

### Random-intercept model with no interaction and with/without individual trials nested under participants

This model fits the factors intensity and condition with random intercepts and fixed slopes. We first compared fitting the model by REML vs. ML, and the models were similar, but not equivalent, with ML resulting in the better fit. The model with individual EMG channels nested under participants resulted in a better fit compared with a participant-level model only:

```
## $statistics
##                          aic      bic bayes.factor
## no_interaction_REML 38865.71 38905.92        0.236
## no_interaction_ML   38862.82 38903.03        4.245
##
## $predicted_differences
##    0%   25%   50%   75%  100%
## 0.000 0.001 0.001 0.001 0.002
```

```
## $statistics
##                                aic      bic bayes.factor
## no_interaction_ML         38862.82 38903.03            0
## no_interaction_ML_channel 33875.84 33922.76          Inf
##
## $predicted_differences
##     0%    25%    50%    75%   100%
##  0.001  1.213  2.625  4.363 54.565
```

### Random-intercept model with interaction

This model fits the factors intensity, condition, and the intensity x condition interaction with random intercepts and fixed slopes. The random-intercept model with an intensity:condition interaction resulted in a better fit:

```
## $statistics
##                                aic      bic bayes.factor
## no_interaction_ML_channel 33875.84 33922.76 0.000000e+00
## interaction_ML_channel    33725.99 33786.31 4.246506e+29
##
## $predicted_differences
##    0%   25%   50%   75%  100%
## 0.206 0.312 0.401 0.703 0.810
```

#### Model summary

```
## Linear mixed model fit by maximum likelihood . t-tests use Satterthwaite's
##   method [lmerModLmerTest]
## Formula: outcome ~ intensity * condition + (1 | pid/channel)
##    Data: d
##
##      AIC      BIC   logLik deviance df.resid
##  33726.0  33786.3 -16854.0  33708.0     6009
##
## Scaled residuals:
##     Min      1Q  Median      3Q     Max
## -9.2449 -0.5198 -0.0324  0.4769  9.6938
##
## Random effects:
##  Groups      Name        Variance Std.Dev.
##  channel:pid (Intercept) 27.115   5.207
##  pid         (Intercept) 23.498   4.848
##  Residual                 9.707   3.116
## Number of obs: 6018, groups:  channel:pid, 1003; pid, 17
##
```

```
## Fixed effects:
##                        Estimate Std. Error        df t value Pr(>|t|)
## (Intercept)             18.3795     1.1912   17.1949  15.429 1.66e-11 ***
## intensity2              11.5778     0.1391 5015.0002  83.220  < 2e-16 ***
## condition2              -1.5455     0.1391 5015.0002 -11.109  < 2e-16 ***
## condition3               1.2108     0.1391 5015.0002   8.703  < 2e-16 ***
## intensity2:condition2   -0.1869     0.1967 5015.0002  -0.950    0.342
## intensity2:condition3    2.0302     0.1967 5015.0002  10.318  < 2e-16 ***
## ---
## Signif. codes:  0 '***' 0.001 '**' 0.01 '*' 0.05 '.' 0.1 ' ' 1
##
## Correlation of Fixed Effects:
##             (Intr) intns2 cndtn2 cndtn3 int2:2
## intensity2  -0.058
## condition2  -0.058  0.500
## condition3  -0.058  0.500  0.500
## intnsty2:c2  0.041 -0.707 -0.707 -0.354
## intnsty2:c3  0.041 -0.707 -0.354 -0.707  0.500
```

#### Model- & sandwich-based standard errors and p-values

```
## Model- & sandwich-based standard errors and p-values
## Click on the arrow for sandwich-based p-values.
```

\## # A tibble: 6 × 6
\##	term	estimate `Model-based s.e.` `Model-based p-value`
\##	<chr>	<dbl>	<dbl>	<dbl>
\## 1 (Intercept)	18.4	1.19	1.05e-52
\## 2 intensity2	11.6	0.139	0
\## 3 condition2	-1.55	0.139	2.15e-28
\## 4 condition3	1.21	0.139	4.12e-18
\## 5 intensity2:condition2	-0.187	0.197	3.42e- 1
\## 6 intensity2:condition3	2.03	0.197	9.35e-25
\##	`Sandwich s.e.` `Sandwich p-value`
\##	<dbl>	<dbl>
\## 1	1.11	1.80e-11
\## 2	0.753	5.31e-11
\## 3	0.399	1.33e- 3
\## 4	0.458	1.77e- 2
\## 5	0.683	7.88e- 1
\## 6	0.738	1.42e- 2

## Motor unit discharge rate of matched motor units model fitting and results

Fixed effects were fitted using a linear mixed-effects model with individual motor units nested under participants. Model-based standard errors and p-values are presented. Sandwich variance estimation-based standard errors and p-values were also produced; these do not assume normality of the data. A Wald statistic based on the sandwich variance estimate was also produced to test the effect of factor condition.

### Random-intercept model with no interaction and with/without individual motor units nested under participants

This model fits the factors decomp (i.e. decomposition algorithm), intensity, and condition with random intercepts and fixed slopes. We first compared fitting the model by REML vs. ML, and ML resulted in a better fit with individual motor units nested under participants:

```
## $statistics
##                          aic      bic bayes.factor
## no_interaction_REML 15145.24 15195.27        0.000
## no_interaction_ML   15129.50 15179.54     2610.779
##

```

```
## $predicted_differences
##    0%   25%   50%   75%  100%
## 0.000 0.000 0.000 0.001 0.005
## $statistics
##                            aic      bic bayes.factor
## no_interaction_ML     15129.50 15179.54  1.65922e+47
## no_interaction_ML_pid 15353.21 15396.99  0.00000e+00
##
## $predicted_differences
##    0%   25%   50%   75%  100%
## 0.000 0.136 0.325 0.601 1.851
```

### Random-intercept model with interaction

This model fits the factors decomp, intensity, condition, and the intensity x condition interaction with random intercepts and fixed slopes. The random-intercept model with an intensity:condition interaction resulted in a better fit:

```
## $statistics
##                        aic      bic bayes.factor
## no_interaction_ML 15129.50 15179.54            0
## interaction_ML    15078.11 15140.65    277456497
##
## $predicted_differences
##    0%   25%   50%   75%  100%
## 0.002 0.043 0.204 0.236 0.299
```

#### Model summary

```
## Linear mixed model fit by maximum likelihood . t-tests use Satterthwaite's
##   method [lmerModLmerTest]
## Formula: outcome ~ decomp + intensity * condition + (1 | pid/muid)
##    Data: d
##
##      AIC      BIC   logLik deviance df.resid
##  15078.1  15140.7  -7529.1  15058.1     3833
##
## Scaled residuals:
##     Min      1Q  Median      3Q     Max
## -5.7371 -0.5693  0.0247  0.6186  3.5884
##
## Random effects:
##  Groups   Name        Variance Std.Dev.
##  muid:pid (Intercept) 0.5358   0.732
##  pid      (Intercept) 2.1004   1.449
##  Residual             2.5837   1.607
## Number of obs: 3843, groups:  muid:pid, 432; pid, 17
##
```

```
## Fixed effects:
##                         Estimate Std. Error         df t value Pr(>|t|)
## (Intercept)             12.48555    0.36069   18.03581  34.616  < 2e-16 ***
## decompMUedit            -0.06328    0.05566 3733.40749  -1.137  0.25566
## intensity2               2.24128    0.09100 3450.54130  24.629  < 2e-16 ***
## condition2              -1.15337    0.08617 3381.13078 -13.386  < 2e-16 ***
## condition3              -0.04339    0.08617 3381.13078  -0.504  0.61459
## intensity2:condition2    0.35230    0.12751 3381.13078   2.763  0.00576 **
## intensity2:condition3    0.94287    0.12751 3381.13078   7.395 1.78e-13 ***
## ---
## Signif. codes:  0 '***' 0.001 '**' 0.01 '*' 0.05 '.' 0.1 ' ' 1
##
## Correlation of Fixed Effects:
##             (Intr) dcmpMU intns2 cndtn2 cndtn3 int2:2
## decompMUedt -0.096
## intensity2  -0.111 -0.023
## condition2  -0.119  0.000  0.473
## condition3  -0.119  0.000  0.473  0.500
## intnsty2:c2  0.081  0.000 -0.701 -0.676 -0.338
## intnsty2:c3  0.081  0.000 -0.701 -0.338 -0.676  0.500
```

#### Model- & sandwich-based standard errors and p-values

```
## Model- & sandwich-based standard errors and p-values
## Click on the arrow for sandwich-based p-values.
```

```
## # A tibble: 7 × 6
##   term                  estimate `Model-based s.e.` `Model-based p-value`
##   <chr>                    <dbl>              <dbl>                 <dbl>
## 1 (Intercept)            12.5                0.361              1.03e-228
## 2 decompMUedit           -0.0633             0.0557             2.56e-  1
## 3 intensity2              2.24               0.0910             1.81e-124
## 4 condition2             -1.15               0.0862             5.73e- 40
## 5 condition3             -0.0434             0.0862             6.15e-  1
## 6 intensity2:condition2   0.352              0.128              5.75e-  3
## 7 intensity2:condition3   0.943              0.128              1.73e- 13
```

```
##   `Sandwich s.e.` `Sandwich p-value`
##             <dbl>              <dbl>
## 1          0.350            1.13e-16
## 2          0.0713           3.89e- 1
## 3          0.251            3.37e- 7
## 4          0.226            1.54e- 4
## 5          0.195            8.27e- 1
## 6          0.379            3.68e- 1
## 7          0.252            2.14e- 3
```

#### Interaction, intensity & condition p-values

The Wald statistics testing for an effect of the intensity:condition interaction term, for an effect of the intensity term and the intensity:condition interaction term, and for an effect of the condition term and the intensity:condition interaction term are:

```
##  test Fstat df_num df_denom     p_val sig
##   HTZ  15.3      2     13.3 0.0003606 ***
##  test Fstat df_num df_denom   p_val sig
##   HTZ  99.1      3     12.2 < 1e-04 ***
##  test Fstat df_num df_denom   p_val sig
##   HTZ  17.5      4     11.2 < 1e-04 ***
```

#### Post-hoc comparisons

Estimated marginal means (EMMs) for the interaction between intensity and condition for the random-intercept model, while explicitly using cluster-robust standard errors (which accounts for non-independent data and heteroskedasticity), are reported below. This approach was used to ensure that the confidence intervals and subsequent p-values for the EMMs are more accurate and reliable.

```
##  intensity condition emmean    SE   df lower.CL upper.CL
##  1         1           12.5 0.352 17.7     11.7     13.2
##  2         1           14.7 0.444 17.9     13.8     15.6
##  1         2           11.3 0.362 17.7     10.5     12.1
##  2         2           13.9 0.465 17.9     12.9     14.9
##  1         3           12.4 0.365 17.7     11.6     13.2
##  2         3           15.6 0.438 17.9     14.7     16.5
##
## Results are averaged over the levels of: decomp
## Degrees-of-freedom method: satterthwaite
## Confidence level used: 0.95
```

```
## condition = 1:
##  contrast                estimate    SE   df t.ratio p.value
##  intensity1 - intensity2    -2.24 0.251 3451  -8.932  <.0001
##
## condition = 2:
##  contrast                estimate    SE   df t.ratio p.value
##  intensity1 - intensity2    -2.59 0.261 3451  -9.943  <.0001
##
## condition = 3:
##  contrast                estimate    SE   df t.ratio p.value
##  intensity1 - intensity2    -3.18 0.173 3451 -18.373  <.0001
##
## Results are averaged over the levels of: decomp
## Degrees-of-freedom method: satterthwaite
## intensity = 1:
##  contrast                estimate    SE   df t.ratio p.value
##  condition1 - condition2   1.1534 0.226 3381   5.097  <.0001
##  condition1 - condition3   0.0434 0.195 3381   0.223  0.8237
##  condition2 - condition3  -1.1100 0.254 3381  -4.362  <.0001
##
## intensity = 2:
##  contrast                estimate    SE   df t.ratio p.value
##  condition1 - condition2   0.8011 0.322 3381   2.485  0.0130
##  condition1 - condition3  -0.8995 0.205 3381  -4.381  <.0001
##  condition2 - condition3  -1.7006 0.278 3381  -6.124  <.0001
##
## Results are averaged over the levels of: decomp
## Degrees-of-freedom method: satterthwaite
## P value adjustment: holm method for 3 tests
```

### Random-slope-and-intercept model with interaction

This model fits the factors decomp, intensity, condition, and the intensity x condition interaction with random intercepts and random slopes. This random-slope-and-intercept model resulted in a better fit than the random-intercept model with an intensity:condition interaction:

```
## $statistics
##                                 aic      bic bayes.factor
## interaction_ML             15078.11 15140.65            0
## interaction_ML_randomSlope 12470.06 12870.31          Inf
##
## $predicted_differences
##    0%   25%   50%   75%  100%
## 0.001 0.371 0.810 1.432 6.894
```

#### Model summary

```
## Linear mixed model fit by maximum likelihood . t-tests use Satterthwaite's
##   method [lmerModLmerTest]
## Formula: outcome ~ decomp + intensity * condition + (decomp + intensity *
##     condition | pid/muid)
##    Data: d
##
##      AIC      BIC   logLik deviance df.resid
##  12470.1  12870.3  -6171.0  12342.1     3779
##
## Scaled residuals:
##     Min      1Q  Median      3Q     Max
## -4.6614 -0.4376  0.0154  0.4743  3.2429
##
## Random effects:
##  Groups   Name                  Variance Std.Dev. Corr
##  muid:pid (Intercept)           1.566513 1.2516
##           decompMUedit          3.002157 1.7327   -0.70
##           intensity2            3.433880 1.8531   -0.38 -0.11
##           condition2            0.022519 0.1501    0.67  0.05 -0.54
##           condition3            0.007345 0.0857    0.82 -0.17 -0.58  0.97
##           intensity2:condition2 0.055016 0.2346   -0.67  0.06  0.92 -0.80 -0.85
##           intensity2:condition3 0.008760 0.0936   -0.89  0.34  0.40 -0.92 -0.97
##  pid      (Intercept)           2.004604 1.4158
##           decompMUedit          0.023477 0.1532    0.17
##           intensity2            0.543085 0.7369    0.07 -0.53
##           condition2            0.874670 0.9352   -0.33 -0.32  0.33
##           condition3            0.491262 0.7009   -0.18 -0.78  0.35  0.09
##           intensity2:condition2 2.052045 1.4325    0.39  0.36 -0.76 -0.70 -0.33
##           intensity2:condition3 0.924246 0.9614    0.33  0.36 -0.66 -0.49 -0.39
##  Residual                       0.680998 0.8252
##
##
##
##
##
##
##
##   0.73
##
##
##
##
##
##
##   0.81
##
## Number of obs: 3843, groups:  muid:pid, 432; pid, 17
##
```

```
## Fixed effects:
##                        Estimate Std. Error        df t value Pr(>|t|)
## (Intercept)           12.479601   0.353224 16.609398  35.331  < 2e-16 ***
## decompMUedit          -0.037544   0.105607 48.376502  -0.356  0.72375
## intensity2             2.290151   0.210202 16.693069  10.895 5.27e-09 ***
## condition2            -1.268866   0.231855 16.735303  -5.473 4.36e-05 ***
## condition3            -0.007047   0.176445 16.850675  -0.040  0.96861
## intensity2:condition2  0.428169   0.354794 16.874634   1.207  0.24415
## intensity2:condition3  0.997032   0.243569 16.398367   4.093  0.00081 ***
## ---
## Signif. codes:  0 '***' 0.001 '**' 0.01 '*' 0.05 '.' 0.1 ' ' 1
##
## Correlation of Fixed Effects:
##             (Intr) dcmpMU intns2 cndtn2 cndtn3 int2:2
## decompMUedt -0.094
## intensity2   0.011 -0.201
## condition2  -0.326 -0.114  0.290
## condition3  -0.185 -0.272  0.306  0.113
## intnsty2:c2  0.380  0.126 -0.650 -0.704 -0.332
## intnsty2:c3  0.312  0.130 -0.577 -0.477 -0.409  0.789
## optimizer (nloptwrap) convergence code: 0 (OK)
## boundary (singular) fit: see help('isSingular')
```

#### Model- & sandwich-based standard errors and p-values

```
## Model- & sandwich-based standard errors and p-values
## Click on the arrow for sandwich-based p-values.
```

```
## # A tibble: 7 × 6
##   term                  estimate `Model-based s.e.` `Model-based p-value`
##   <chr>                    <dbl>              <dbl>                 <dbl>
## 1 (Intercept)           12.5                  0.353             1.61e-236
## 2 decompMUedit          -0.0375               0.106             7.22e-  1
## 3 intensity2             2.29                 0.210             3.08e- 27
## 4 condition2            -1.27                 0.232             4.72e-  8
## 5 condition3            -0.00705              0.176             9.68e-  1
## 6 intensity2:condition2  0.428                0.355             2.28e-  1
## 7 intensity2:condition3  0.997                0.244             4.34e-  5
```

```
##   `Sandwich s.e.` `Sandwich p-value`
##             <dbl>              <dbl>
## 1          0.361            1.82e-16
## 2          0.0853           6.66e- 1
## 3          0.216            1.39e- 8
## 4          0.239            7.06e- 5
## 5          0.182            9.70e- 1
## 6          0.366            2.59e- 1
## 7          0.251            1.10e- 3
```

#### Interaction, intensity & condition p-values

The Wald statistics testing for an effect of the intensity:condition interaction term, for an effect of the intensity term and the intensity:condition interaction term, and for an effect of the condition term and the intensity:condition interaction term are:

```
##  test Fstat df_num df_denom     p_val sig
##   HTZ  12.1      2       15 0.0007342 ***
##  test Fstat df_num df_denom   p_val sig
##   HTZ  81.4      3     13.8 < 1e-04 ***
##  test Fstat df_num df_denom   p_val sig
##   HTZ  16.2      4       13 < 1e-04 ***
```

#### Post-hoc comparisons

Estimated marginal means (EMMs) for the interaction between intensity and condition for the random-slope-and-intercept model, while explicitly using cluster-robust standard errors (which accounts for non-independent data and heteroskedasticity), are reported below. This approach was used to ensure that the confidence intervals and subsequent p-values for the EMMs are more accurate and reliable.

```
##  intensity condition emmean    SE   df lower.CL upper.CL
##  1         1           12.5 0.363 16.7     11.7     13.2
##  2         1           14.8 0.419 16.8     13.9     15.6
##  1         2           11.2 0.359 16.8     10.4     12.0
##  2         2           13.9 0.466 16.7     12.9     14.9
##  1         3           12.5 0.367 16.7     11.7     13.2
##  2         3           15.7 0.476 16.4     14.7     16.7
##
## Results are averaged over the levels of: decomp
## Degrees-of-freedom method: satterthwaite
## Confidence level used: 0.95
```

```
## condition = 1:
##  contrast                estimate    SE   df t.ratio p.value
##  intensity1 - intensity2    -2.29 0.216 16.7 -10.598  <.0001
##
## condition = 2:
##  contrast                estimate    SE   df t.ratio p.value
##  intensity1 - intensity2    -2.72 0.279 15.0  -9.758  <.0001
##
## condition = 3:
##  contrast                estimate    SE   df t.ratio p.value
##  intensity1 - intensity2    -3.29 0.215 13.3 -15.296  <.0001
##
## Results are averaged over the levels of: decomp
## Degrees-of-freedom method: satterthwaite
## intensity = 1:
##  contrast                estimate    SE   df t.ratio p.value
##  condition1 - condition2  1.26887 0.239 16.7   5.309  0.0002
##  condition1 - condition3  0.00705 0.182 16.9   0.039  0.9695
##  condition2 - condition3 -1.26182 0.284 16.8  -4.450  0.0007
##
## intensity = 2:
##  contrast                estimate    SE   df t.ratio p.value
##  condition1 - condition2  0.84070 0.260 17.2   3.227  0.0049
##  condition1 - condition3 -0.98998 0.242 16.1  -4.084  0.0017
##  condition2 - condition3 -1.83068 0.271 16.9  -6.756  <.0001
##
## Results are averaged over the levels of: decomp
## Degrees-of-freedom method: satterthwaite
## P value adjustment: holm method for 3 tests
```

\## intensity = 1:
\## contrast	estimate	SE	df lower.CL upper.CL
\## condition1 - condition2 1.26887 0.239 16.7	0.633	1.904
\## condition1 - condition3 0.00705 0.182 16.9	-0.476	0.490
\## condition2 - condition3 -1.26182 0.284 16.8	-2.015	-0.508
\##
\## intensity = 2:
\## contrast	estimate	SE	df lower.CL upper.CL
\## condition1 - condition2 0.84070 0.260 17.2	0.150	1.531
\## condition1 - condition3 -0.98998 0.242 16.1	-1.637	-0.343
\## condition2 - condition3 -1.83068 0.271 16.9	-2.551	-1.111
\##
\## Results are averaged over the levels of: decomp
\## Degrees-of-freedom method: satterthwaite
\## Confidence level used: 0.95
\## Conf-level adjustment: bonferroni method for 3 estimates

## Coefficient of variation in motor unit discharge rate model fitting and results

Fixed effects were fitted using a linear mixed-effects model with individual motor units nested under participants. Model-based standard errors and p-values are presented. Sandwich variance estimation-based standard errors and p-values were also produced; these do not assume normality of the data. A Wald statistic based on the sandwich variance estimate was also produced to test the effect of factor condition.

### Random-intercept model with no interaction and with/without individual motor units nested under participants

This fixed-effects model fits the factors decomp (i.e. decomposition algorithm), intensity, and condition with random intercepts and fixed slopes. We first compared fitting the model by REML vs. ML, and ML resulted in a better fit with individual motor units nested under participants:

```
## $statistics
##                          aic      bic bayes.factor
## no_interaction_REML 22662.33 22712.36        0.024
## no_interaction_ML   22654.85 22704.88       42.054
##

```

```
## $predicted_differences
##    0%   25%   50%   75%  100%
## 0.000 0.001 0.002 0.004 0.009
```

```
## $statistics
##                            aic      bic bayes.factor
## no_interaction_ML     22654.85 22704.88  2.28552e+31
## no_interaction_ML_pid 22805.51 22849.29  0.00000e+00
##
## $predicted_differences
##    0%   25%   50%   75%  100%
## 0.001 0.396 0.861 1.382 5.907
```

### Random-intercept model with interaction

This model fits the factors decomp, intensity, condition, and the intensity x condition interaction with random intercepts and fixed slopes. The random-intercept model with an intensity:condition interaction did not result in a better fit:

```
## $statistics
##                        aic      bic bayes.factor
## no_interaction_ML 22654.85 22704.88      687.343
## interaction_ML    22655.40 22717.94        0.001
##
## $predicted_differences
##    0%   25%   50%   75%  100%
## 0.002 0.008 0.145 0.170 0.183
```

#### Model summary

```
## Linear mixed model fit by maximum likelihood . t-tests use Satterthwaite's
##   method [lmerModLmerTest]
## Formula: outcome ~ decomp + intensity * condition + (1 | pid/muid)
##    Data: d
##
##      AIC      BIC   logLik deviance df.resid
##  22655.4  22717.9 -11317.7  22635.4     3833
##
## Scaled residuals:
##     Min      1Q  Median      3Q     Max
## -2.2830 -0.6232 -0.1498  0.4147 11.2493
##
## Random effects:
##  Groups   Name        Variance Std.Dev.
##  muid:pid (Intercept)  3.029   1.740
##  pid      (Intercept)  2.795   1.672
##  Residual             19.009   4.360
## Number of obs: 3843, groups:  muid:pid, 432; pid, 17
##
```

```
## Fixed effects:
##                        Estimate Std. Error        df t value Pr(>|t|)
## (Intercept)             12.6212     0.4579   23.4719  27.564   <2e-16 ***
## decompMUedit             3.4761     0.1502 3757.7769  23.143   <2e-16 ***
## intensity2               2.9287     0.2466 3444.0004  11.876   <2e-16 ***
## condition2               0.4956     0.2337 3365.3257   2.120   0.0340 *
## condition3              -0.4943     0.2337 3365.3257  -2.115   0.0345 *
## intensity2:condition2   -0.6414     0.3459 3365.3257  -1.855   0.0638 .
## intensity2:condition3   -0.3417     0.3459 3365.3257  -0.988   0.3233
## ---
## Signif. codes:  0 '***' 0.001 '**' 0.01 '*' 0.05 '.' 0.1 ' ' 1
##
## Correlation of Fixed Effects:
##             (Intr) dcmpMU intns2 cndtn2 cndtn3 int2:2
## decompMUedt -0.204
## intensity2  -0.239 -0.022
## condition2  -0.255  0.000  0.474
## condition3  -0.255  0.000  0.474  0.500
## intnsty2:c2  0.172  0.000 -0.701 -0.676 -0.338
## intnsty2:c3  0.172  0.000 -0.701 -0.338 -0.676  0.500
```

#### Model- & sandwich-based standard errors and p-values

```
## Model- & sandwich-based standard errors and p-values
## Click on the arrow for sandwich-based p-values.
```

```
## # A tibble: 7 × 6
##   term                  estimate `Model-based s.e.` `Model-based p-value`
##   <chr>                    <dbl>              <dbl>                 <dbl>
## 1 (Intercept)             12.6                0.458             9.57e-153
## 2 decompMUedit             3.48               0.150             5.05e-111
## 3 intensity2               2.93               0.247             5.68e- 32
## 4 condition2               0.496              0.234             3.40e-  2
## 5 condition3              -0.494              0.234             3.45e-  2
## 6 intensity2:condition2   -0.641              0.346             6.37e-  2
## 7 intensity2:condition3   -0.342              0.346             3.23e-  1
```

```
##   `Sandwich s.e.` `Sandwich p-value`
##             <dbl>              <dbl>
## 1           0.594           4.23e-13
## 2           0.286           5.32e- 9
## 3           0.549           1.01e- 4
## 4           0.614           4.32e- 1
## 5           0.376           2.09e- 1
## 6           0.719           3.87e- 1
## 7           0.427           4.36e- 1
```

#### Interaction, intensity & condition p-values

The Wald statistics testing for an effect of the intensity:condition interaction term, for an effect of the intensity term and the intensity:condition interaction term, and for an effect of the condition term and the intensity:condition interaction term are:

```
##  test Fstat df_num df_denom  p_val sig
##   HTZ 0.402      2     13.3 0.6771
##  test Fstat df_num df_denom     p_val sig
##   HTZ    11      3     12.2 0.0008918 ***
##  test Fstat df_num df_denom  p_val sig
##   HTZ  1.36      4     11.2 0.3082
```

#### Post-hoc comparisons

Estimated marginal means (EMMs) for the interaction between intensity and condition for the random-intercept model, while explicitly using cluster-robust standard errors (which accounts for non-independent data and heteroskedasticity), are reported below. This approach was used to ensure that the confidence intervals and subsequent p-values for the EMMs are more accurate and reliable.

```
##  intensity condition emmean    SE   df lower.CL upper.CL
##  1         1           14.4 0.575 21.6     13.2     15.6
##  2         1           17.3 0.707 23.0     15.8     18.8
##  1         2           14.9 0.587 21.6     13.6     16.1
##  2         2           17.1 0.740 23.0     15.6     18.7
##  1         3           13.9 0.419 21.6     13.0     14.7
##  2         3           16.5 0.506 23.0     15.4     17.5
##
## Results are averaged over the levels of: decomp
## Degrees-of-freedom method: satterthwaite
## Confidence level used: 0.95
```

```
## condition = 1:
##  contrast                estimate    SE   df t.ratio p.value
##  intensity1 - intensity2    -2.93 0.549 3444  -5.333  <.0001
##
## condition = 2:
##  contrast                estimate    SE   df t.ratio p.value
##  intensity1 - intensity2    -2.29 0.859 3444  -2.664  0.0078
##
## condition = 3:
##  contrast                estimate    SE   df t.ratio p.value
##  intensity1 - intensity2    -2.59 0.515 3444  -5.022  <.0001
##
## Results are averaged over the levels of: decomp
## Degrees-of-freedom method: satterthwaite
## intensity = 1:
##  contrast                estimate    SE   df t.ratio p.value
##  condition1 - condition2   -0.496 0.614 3365  -0.808  0.4193
##  condition1 - condition3    0.494 0.376 3365   1.315  0.3773
##  condition2 - condition3    0.990 0.459 3365   2.157  0.0932
##
## intensity = 2:
##  contrast                estimate    SE   df t.ratio p.value
##  condition1 - condition2    0.146 0.460 3365   0.317  0.7515
##  condition1 - condition3    0.836 0.460 3365   1.816  0.2084
##  condition2 - condition3    0.690 0.511 3365   1.351  0.3538
##
## Results are averaged over the levels of: decomp
## Degrees-of-freedom method: satterthwaite
## P value adjustment: holm method for 3 tests
```

### Random-slope-and-intercept model with interaction

This model fits the factors decomp, intensity, condition, and the intensity x condition interaction with random intercepts and random slopes. This random-slope-and-intercept model resulted in a better fit than the random-intercept model with an intensity:condition interaction:

```
## $statistics
##                                 aic      bic bayes.factor
## interaction_ML             22655.40 22717.94   0.0000e+00
## interaction_ML_randomSlope 22119.42 22519.68   1.1307e+43
##
## $predicted_differences
##     0%    25%    50%    75%   100%
##  0.000  0.622  1.307  2.265 14.419
```

#### Model summary

```
## Linear mixed model fit by maximum likelihood . t-tests use Satterthwaite's
##   method [lmerModLmerTest]
## Formula: outcome ~ decomp + intensity * condition + (decomp + intensity *
##     condition | pid/muid)
##    Data: d
##
##      AIC      BIC   logLik deviance df.resid
##  22119.4  22519.7 -10995.7  21991.4     3779
##
## Scaled residuals:
##     Min      1Q  Median      3Q     Max
## -3.9715 -0.5359 -0.1364  0.3580  9.7362
##
## Random effects:
##  Groups   Name                  Variance Std.Dev. Corr
##  muid:pid (Intercept)            3.5346  1.8800
##           decompMUedit           8.5600  2.9257   -0.52
##           intensity2             6.7104  2.5904   -0.17 -0.20
##           condition2             0.7211  0.8492    0.98 -0.34 -0.14
##           condition3             0.1444  0.3801    0.74 -0.25 -0.78  0.69
##           intensity2:condition2  0.8810  0.9386   -0.67  0.36  0.79 -0.58 -0.98
##           intensity2:condition3  0.9858  0.9929   -0.74  0.20 -0.40 -0.82 -0.16
##  pid      (Intercept)            5.4637  2.3375
##           decompMUedit           0.5901  0.7682   -0.28
##           intensity2             2.9403  1.7147   -0.08 -0.28
##           condition2             5.1223  2.2632   -0.49 -0.38 -0.01
##           condition3             1.6637  1.2898   -0.80  0.12 -0.32  0.74
##           intensity2:condition2  5.6484  2.3766    0.22  0.81 -0.01 -0.78 -0.46
##           intensity2:condition3  1.5289  1.2365    0.47  0.58 -0.47 -0.72 -0.34
##  Residual                       12.6492  3.5566
##
##
##
##
##
##
##
##   0.02
##
##
##
##
##
##
##   0.76
##
## Number of obs: 3843, groups:  muid:pid, 432; pid, 17
##
```

```
## Fixed effects:
##                       Estimate Std. Error      df t value Pr(>|t|)
## (Intercept)            12.4421     0.6001 16.8479  20.735 1.99e-13 ***
## decompMUedit            3.6488     0.2748 20.8738  13.278 1.20e-11 ***
## intensity2              2.8781     0.4887 18.0755   5.889 1.39e-05 ***
## condition2              0.7251     0.5864 17.0660   1.237    0.233
## condition3             -0.3985     0.3686 17.1528  -1.081    0.295
## intensity2:condition2  -0.7219     0.6497 16.7563  -1.111    0.282
## intensity2:condition3  -0.5309     0.4187 18.8808  -1.268    0.220
## ---
## Signif. codes:  0 '***' 0.001 '**' 0.01 '*' 0.05 '.' 0.1 ' ' 1
##
## Correlation of Fixed Effects:
##             (Intr) dcmpMU intns2 cndtn2 cndtn3 int2:2
## decompMUedt -0.300
## intensity2  -0.139 -0.206
## condition2  -0.472 -0.260  0.058
## condition3  -0.726  0.059 -0.140  0.685
## intnsty2:c2  0.221  0.512 -0.121 -0.760 -0.430
## intnsty2:c3  0.374  0.308 -0.508 -0.573 -0.446  0.640
## optimizer (nloptwrap) convergence code: 0 (OK)
## boundary (singular) fit: see help('isSingular')
```

#### Model- & sandwich-based standard errors and p-values

```
## Model- & sandwich-based standard errors and p-values
## Click on the arrow for sandwich-based p-values.
```

```
## # A tibble: 7 × 6
##   term                  estimate `Model-based s.e.` `Model-based p-value`
##   <chr>                    <dbl>              <dbl>                 <dbl>
## 1 (Intercept)             12.4                0.600              1.55e-90
## 2 decompMUedit             3.65               0.275              2.33e-39
## 3 intensity2               2.88               0.489              4.23e- 9
## 4 condition2               0.725              0.586              2.16e- 1
## 5 condition3              -0.398              0.369              2.80e- 1
## 6 intensity2:condition2   -0.722              0.650              2.67e- 1
## 7 intensity2:condition3   -0.531              0.419              2.05e- 1
```

```
##   `Sandwich s.e.` `Sandwich p-value`
##             <dbl>              <dbl>
## 1           0.617           8.72e-13
## 2           0.272           6.12e-10
## 3           0.503           3.29e- 5
## 4           0.604           2.48e- 1
## 5           0.374           3.02e- 1
## 6           0.668           2.96e- 1
## 7           0.417           2.22e- 1
```

#### Interaction, intensity & condition p-values

The Wald statistics testing for an effect of the intensity:condition interaction term, for an effect of the intensity term and the intensity:condition interaction term, and for an effect of the condition term and the intensity:condition interaction term are:

```
##  test Fstat df_num df_denom  p_val sig
##   HTZ 0.806      2     14.6 0.4657
##  test Fstat df_num df_denom     p_val sig
##   HTZ  12.3      3     13.6 0.0003646 ***
##  test Fstat df_num df_denom  p_val sig
##   HTZ   1.9      4     12.7 0.1724
```

#### Post-hoc comparisons

Estimated marginal means (EMMs) for the interaction between intensity and condition for the random-slope-and-intercept model, while explicitly using cluster-robust standard errors (which accounts for non-independent data and heteroskedasticity), are reported below. This approach was used to ensure that the confidence intervals and subsequent p-values for the EMMs are more accurate and reliable.

```
##  intensity condition emmean    SE   df lower.CL upper.CL
##  1         1           14.3 0.592 16.9     13.0     15.5
##  2         1           17.1 0.698 17.2     15.7     18.6
##  1         2           15.0 0.563 16.4     13.8     16.2
##  2         2           17.1 0.722 17.6     15.6     18.7
##  1         3           13.9 0.396 16.9     13.0     14.7
##  2         3           16.2 0.547 16.2     15.1     17.4
##
## Results are averaged over the levels of: decomp
## Degrees-of-freedom method: satterthwaite
## Confidence level used: 0.95
```

\## condition = 1:
\## contrast	estimate	SE	df t.ratio p.value
\## intensity1 - intensity2	-2.88 0.503 18.1 -5.725 <.0001
\##
\## condition = 2:
\## contrast	estimate	SE	df t.ratio p.value
\## intensity1 - intensity2	-2.16 0.786 16.9 -2.743 0.0139
\##
\## condition = 3:
\## contrast	estimate	SE	df t.ratio p.value
\## intensity1 - intensity2	-2.35 0.463 17.4 -5.074 0.0001
\##
\## Results are averaged over the levels of: decomp
\## Degrees-of-freedom method: satterthwaite

## Motor unit discharge rate of matched and unique motor units based on the smoothed cumulative spike train model fitting and results

Fixed effects were fitted using a linear mixed-effects model. Model-based standard errors and p-values are presented. Sandwich variance estimation-based standard errors and p-values were also produced; these do not assume normality of the data. A Wald statistic based on the sandwich variance estimate was also produced to test the effect of factor condition.

### Random-intercept model with no interaction

This model fits the factors decomp (i.e. decomposition algorithm), intensity, and condition with random intercepts and fixed slopes. We first compared fitting the model by REML vs. ML, and ML resulted in a better fit:

\## $statistics
\##	aic	bic bayes.factor
\## no_interaction_REML 579.983 603.210	0.008
\## no_interaction_ML	570.252 593.479	129.727
\##
\## $predicted_differences
\##	0%	25%	50%	75% 100%
\## 0.000 0.000 0.001 0.002 0.003

### Random-intercept model with interaction

This model fits the factors decomp, intensity, condition, and the intensity x condition interaction with random intercepts and fixed slopes. The fixed-effects model with an intensity:condition interaction resulted in a marginally better fit:

```
## $statistics
##                       aic     bic bayes.factor
## no_interaction_ML 570.252 593.479        0.201
## interaction_ML    560.407 590.270        4.977
##
## $predicted_differences
##    0%   25%   50%   75%  100%
## 0.002 0.010 0.252 0.257 0.264
```

#### Model summary

```
## Linear mixed model fit by maximum likelihood . t-tests use Satterthwaite's
##   method [lmerModLmerTest]
## Formula: outcome ~ decomp + intensity * condition + (1 | pid)
##    Data: d
##
##      AIC      BIC   logLik deviance df.resid
##    560.4    590.3   -271.2    542.4      195
##
## Scaled residuals:
##     Min      1Q  Median      3Q     Max
## -2.9934 -0.5272  0.0161  0.5463  4.1409
##
## Random effects:
##  Groups   Name        Variance Std.Dev.
##  pid      (Intercept) 1.9219   1.3863
##  Residual             0.6169   0.7855
## Number of obs: 204, groups:  pid, 17
##
## Fixed effects:
##                       Estimate Std. Error       df t value Pr(>|t|)
## (Intercept)            11.4833     0.3664  22.6800  31.344  < 2e-16 ***
## decompMUedit           -0.6787     0.1100 187.0000  -6.171 4.12e-09 ***
## intensity2              1.9754     0.1905 187.0000  10.370  < 2e-16 ***
## condition2             -1.1588     0.1905 187.0000  -6.083 6.52e-09 ***
## condition3              0.1214     0.1905 187.0000   0.637 0.524625
## intensity2:condition2   0.4883     0.2694 187.0000   1.812 0.071540 .
## intensity2:condition3   1.0210     0.2694 187.0000   3.790 0.000203 ***
## ---
## Signif. codes:  0 '***' 0.001 '**' 0.01 '*' 0.05 '.' 0.1 ' ' 1
##
```

```
## Correlation of Fixed Effects:
##             (Intr) dcmpMU intns2 cndtn2 cndtn3 int2:2
## decompMUedt -0.150
## intensity2  -0.260  0.000
## condition2  -0.260  0.000  0.500
## condition3  -0.260  0.000  0.500  0.500
## intnsty2:c2  0.184  0.000 -0.707 -0.707 -0.354
## intnsty2:c3  0.184  0.000 -0.707 -0.354 -0.707  0.500
```

#### Model- & sandwich-based standard errors and p-values

```
## Model- & sandwich-based standard errors and p-values
## Click on the arrow for sandwich-based p-values.
## # A tibble: 7 × 6
##   term                  estimate `Model-based s.e.` `Model-based p-value`
##   <chr>                    <dbl>              <dbl>                 <dbl>
## 1 (Intercept)             11.5                0.366              4.54e-78
## 2 decompMUedit            -0.679              0.110              3.85e- 9
## 3 intensity2               1.98               0.191              2.40e-20
## 4 condition2              -1.16               0.191              6.13e- 9
## 5 condition3               0.121              0.191              5.25e- 1
## 6 intensity2:condition2    0.488              0.269              7.15e- 2
## 7 intensity2:condition3    1.02               0.269              2.01e- 4
##   `Sandwich s.e.` `Sandwich p-value`
##             <dbl>              <dbl>
## 1           0.352           4.52e-16
## 2           0.105           7.42e- 6
## 3           0.242           4.24e- 7
## 4           0.244           2.22e- 4
## 5           0.160           4.60e- 1
## 6           0.381           2.19e- 1
## 7           0.224           3.22e- 4
```

#### Interaction, intensity & condition p-values

The Wald statistics testing for an effect of the intensity:condition interaction term, for an effect of the intensity term and the intensity:condition interaction term, and for an effect of the condition term and the intensity:condition interaction term are:

```
##  test Fstat df_num df_denom     p_val sig
##   HTZ  13.8      2       15 0.0003977 ***
##  test Fstat df_num df_denom   p_val sig
##   HTZ  89.9      3       14 < 1e-04 ***
##  test Fstat df_num df_denom   p_val sig
##   HTZ  16.9      4       13 < 1e-04 ***
```

#### Post-hoc comparisons

Estimated marginal means (EMMs) for the interaction between intensity and condition for the random-intercept model, while explicitly using cluster-robust standard errors (which accounts for non-independent data and heteroskedasticity), are reported below. This approach was used to ensure that the confidence intervals and subsequent p-values for the EMMs are more accurate and reliable.

```
##  intensity condition emmean    SE   df lower.CL upper.CL
##  1         1          11.14 0.334 21.7    10.45     11.8
##  2         1          13.12 0.415 21.7    12.26     14.0
##  1         2           9.99 0.315 21.7     9.33     10.6
##  2         2          12.45 0.472 21.7    11.47     13.4
##  1         3          11.27 0.328 21.7    10.58     11.9
##  2         3          14.26 0.444 21.7    13.34     15.2
##
## Results are averaged over the levels of: decomp
## Degrees-of-freedom method: satterthwaite
## Confidence level used: 0.95
```

```
## condition = 1:
##  contrast                estimate    SE  df t.ratio p.value
##  intensity1 - intensity2    -1.98 0.242 187  -8.166  <.0001
##
## condition = 2:
##  contrast                estimate    SE  df t.ratio p.value
##  intensity1 - intensity2    -2.46 0.332 187  -7.413  <.0001
##
## condition = 3:
##  contrast                estimate    SE  df t.ratio p.value
##  intensity1 - intensity2    -3.00 0.174 187 -17.192  <.0001
##
## Results are averaged over the levels of: decomp
## Degrees-of-freedom method: satterthwaite
```

```
## intensity = 1:
##  contrast                estimate    SE  df t.ratio p.value
##  condition1 - condition2    1.159 0.244 187   4.741  <.0001
##  condition1 - condition3   -0.121 0.160 187  -0.758  0.4496
##  condition2 - condition3   -1.280 0.273 187  -4.688  <.0001
##
## intensity = 2:
##  contrast                estimate    SE  df t.ratio p.value
##  condition1 - condition2    0.671 0.280 187   2.393  0.0177
##  condition1 - condition3   -1.142 0.207 187  -5.518  <.0001
##  condition2 - condition3   -1.813 0.255 187  -7.099  <.0001
##
## Results are averaged over the levels of: decomp
## Degrees-of-freedom method: satterthwaite
## P value adjustment: holm method for 3 tests
```

### Random-slope-and-intercept model with interaction

This model fits the factors decomp, intensity, condition, and the intensity x condition interaction with random intercepts and random slopes. This random-slope-and-intercept model did not necessarily result in a better fit than the random-intercept model with an intensity:condition interaction:

```
## $statistics
##                                aic     bic bayes.factor
## interaction_ML             560.407 590.270     1015.376
## interaction_ML_randomSlope 484.663 604.116        0.001
##
## $predicted_differences
##    0%   25%   50%   75%  100%
## 0.004 0.163 0.380 0.651 2.125
```

#### Model summary

```
## Linear mixed model fit by maximum likelihood . t-tests use Satterthwaite's
##   method [lmerModLmerTest]
## Formula: outcome ~ decomp + intensity * condition + (decomp + intensity *
##     condition | pid)
##    Data: d
##
##      AIC      BIC   logLik deviance df.resid
##    484.7    604.1   -206.3    412.7      168
##
## Scaled residuals:
##     Min      1Q  Median      3Q     Max
## -2.8632 -0.4316  0.0136  0.5134  3.8324
##
```

\## Random effects:
\## Groups	Name	Variance Std.Dev. Corr
\## pid	(Intercept)	1.9135	1.3833
\##	decompMUedit	0.1377	0.3711	-0.45
\##	intensity2	0.8015	0.8953	0.17 -0.78
\##	condition2	0.8126	0.9015	-0.44 0.23 0.10
\##	condition3	0.2757	0.5250	-0.17 -0.49 0.32 0.04
\##	intensity2:condition2 2.0348	1.4265	0.46 -0.07 -0.48 -0.68 -0.30
\##	intensity2:condition3 0.5180	0.7197	0.41 0.30 -0.76 -0.42 -0.34
\## Residual	0.1486	0.3855
\##
\##
\##
\##
\##
\##
\##
\##	0.83
\##
\## Number of obs: 204, groups: pid, 17
\##
\## Fixed effects:
\##	Estimate Std. Error	df t value Pr(>|t|)
\## (Intercept)	11.4833	0.3430 17.0106 33.478 < 2e-16 ***
\## decompMUedit	-0.6787	0.1049 17.6862 -6.468 4.78e-06 ***
\## intensity2	1.9754	0.2364 17.0893	8.356 1.93e-07 ***
\## condition2	-1.1588	0.2378 17.0242 -4.873 0.000142 ***
\## condition3	0.1214	0.1580 17.5762	0.769 0.452293
\## intensity2:condition2	0.4883	0.3704 17.0047	1.318 0.204893
\## intensity2:condition3	1.0210	0.2190 17.4795	4.663 0.000208 ***
\## ---
\## Signif. codes: 0 '***' 0.001 '**' 0.01 '*' 0.05 '.' 0.1 ' ' 1
\##
\## Correlation of Fixed Effects:
\##	(Intr) dcmpMU intns2 cndtn2 cndtn3 int2:2
\## decompMUedt -0.415
\## intensity2	0.098 -0.614
\## condition2 -0.448 0.185 0.165
\## condition3 -0.217 -0.338 0.351 0.149
\## intnsty2:c2 0.451 -0.054 -0.508 -0.680 -0.301
\## intnsty2:c3 0.380 0.208 -0.728 -0.392 -0.471 0.725
\## optimizer (nloptwrap) convergence code: 0 (OK)
\## boundary (singular) fit: see help('isSingular')

#### Model- & sandwich-based standard errors and p-values

```
## Model- & sandwich-based standard errors and p-values
## Click on the arrow for sandwich-based p-values.
## # A tibble: 7 × 6
##   term                  estimate `Model-based s.e.` `Model-based p-value`
##   <chr>                    <dbl>              <dbl>                 <dbl>
## 1 (Intercept)             11.5                0.343              3.09e-76
## 2 decompMUedit            -0.679              0.105              1.04e- 9
## 3 intensity2               1.98               0.236              2.34e-14
## 4 condition2              -1.16               0.238              2.52e- 6
## 5 condition3               0.121              0.158              4.43e- 1
## 6 intensity2:condition2    0.488              0.370              1.89e- 1
## 7 intensity2:condition3    1.02               0.219              6.34e- 6
##   `Sandwich s.e.` `Sandwich p-value`
##             <dbl>              <dbl>
## 1           0.352           4.52e-16
## 2           0.105           7.42e- 6
## 3           0.242           4.24e- 7
## 4           0.244           2.22e- 4
## 5           0.160           4.60e- 1
## 6           0.381           2.19e- 1
## 7           0.224           3.22e- 4
```

#### Interaction, intensity & condition p-values

The Wald statistics testing for an effect of the intensity:condition interaction term, for an effect of the intensity term and the intensity:condition interaction term, and for an effect of the condition term and the intensity:condition interaction term are:

```
##  test Fstat df_num df_denom     p_val sig
##   HTZ  13.8      2       15 0.0003977 ***
##  test Fstat df_num df_denom   p_val sig
##   HTZ  89.9      3       14 < 1e-04 ***
##  test Fstat df_num df_denom   p_val sig
##   HTZ  16.9      4       13 < 1e-04 ***
```

#### Post-hoc comparisons

Estimated marginal means (EMMs) for the interaction between intensity and condition for the random-slope-and-intercept model, while explicitly using cluster-robust standard errors (which accounts for non-independent data and heteroskedasticity), are reported below. This approach was used to ensure that the confidence intervals and subsequent p-values for the EMMs are more accurate and reliable.

```
##  intensity condition emmean    SE df lower.CL upper.CL
##  1         1          11.14 0.334 17    10.44     11.8
##  2         1          13.12 0.415 17    12.24     14.0
##  1         2           9.99 0.315 17     9.32     10.7
##  2         2          12.45 0.472 17    11.45     13.4
##  1         3          11.27 0.328 17    10.57     12.0
##  2         3          14.26 0.444 17    13.32     15.2
##
## Results are averaged over the levels of: decomp
## Degrees-of-freedom method: satterthwaite
## Confidence level used: 0.95
## condition = 1:
##  contrast                estimate    SE   df t.ratio p.value
##  intensity1 - intensity2    -1.98 0.242 17.1  -8.166  <.0001
##
## condition = 2:
##  contrast                estimate    SE   df t.ratio p.value
##  intensity1 - intensity2    -2.46 0.332 17.0  -7.413  <.0001
##
## condition = 3:
##  contrast                estimate    SE   df t.ratio p.value
##  intensity1 - intensity2    -3.00 0.174 17.0 -17.192  <.0001
##
## Results are averaged over the levels of: decomp
## Degrees-of-freedom method: satterthwaite
```

```
## intensity = 1:
##  contrast                estimate    SE   df t.ratio p.value
##  condition1 - condition2    1.159 0.244 17.0   4.741  0.0006
##  condition1 - condition3   -0.121 0.160 17.6  -0.758  0.4587
##  condition2 - condition3   -1.280 0.273 17.0  -4.688  0.0006
##
## intensity = 2:
##  contrast                estimate    SE   df t.ratio p.value
##  condition1 - condition2    0.671 0.280 17.0   2.393  0.0285
##  condition1 - condition3   -1.142 0.207 17.0  -5.518  0.0001
##  condition2 - condition3   -1.813 0.255 17.0  -7.099  <.0001
##
## Results are averaged over the levels of: decomp
## Degrees-of-freedom method: satterthwaite
## P value adjustment: holm method for 3 tests
```

\## intensity = 1:
\## contrast	estimate	SE	df lower.CL upper.CL
\## condition1 - condition2	1.159 0.244 17.0	0.5099	1.808
\## condition1 - condition3	-0.121 0.160 17.6 -0.5454	0.303
\## condition2 - condition3	-1.280 0.273 17.0 -2.0052	-0.555
\##
\## intensity = 2:
\## contrast	estimate	SE	df lower.CL upper.CL
\## condition1 - condition2	0.671 0.280 17.0 -0.0734	1.414
\## condition1 - condition3	-1.142 0.207 17.0 -1.6921	-0.593
\## condition2 - condition3	-1.813 0.255 17.0 -2.4909	-1.135
\##
\## Results are averaged over the levels of: decomp
\## Degrees-of-freedom method: satterthwaite
\## Confidence level used: 0.95
\## Conf-level adjustment: bonferroni method for 3 estimates

## Active torque estimation from EMG amplitude in the REF condition model fitting and results

Fixed effects were fitted using a linear mixed-effects model. Model-based standard errors and p-values are presented. Sandwich variance estimation-based standard errors and p-values were also produced; these do not assume normality of the data. A Wald statistic based on the sandwich variance estimate was also produced to test the effect of factor condition.

### Random-intercept model with no interaction and without individual trials nested under participants

This model fits the factor EMG amplitude with random intercepts and fixed slopes. We first compared fitting the model by REML vs. ML, and the model fits were essentially equivalent:

```
## $statistics
##                         aic     bic bayes.factor
## no_interaction_REML 228.492 234.598        1.178
## no_interaction_ML   228.819 234.925        0.849
##
## $predicted_differences
##    0%   25%   50%   75%  100%
## 0.000 0.004 0.014 0.024 0.049
```

#### Model summary

```
## Linear mixed model fit by maximum likelihood . t-tests use Satterthwaite's
##   method [lmerModLmerTest]
## Formula: torque ~ emg + (1 | pid)
##    Data: d
##
##      AIC      BIC   logLik deviance df.resid
##    228.8    234.9   -110.4    220.8       30
##
## Scaled residuals:
##      Min       1Q   Median       3Q      Max
## -1.53041 -0.68110  0.07319  0.55054  1.36397
##
## Random effects:
##  Groups   Name        Variance Std.Dev.
##  pid      (Intercept) 44.06    6.638
##  Residual             14.61    3.822
## Number of obs: 34, groups:  pid, 17
##
## Fixed effects:
##             Estimate Std. Error      df t value Pr(>|t|)
## (Intercept)  -5.5444     3.0679 31.2763  -1.807   0.0804 .
## emg           1.4549     0.1046 16.6257  13.909 1.39e-10 ***
## ---
## Signif. codes:  0 '***' 0.001 '**' 0.01 '*' 0.05 '.' 0.1 ' ' 1
##
## Correlation of Fixed Effects:
##     (Intr)
## emg -0.824
```

#### Model- & sandwich-based standard errors and p-values

The full cluster-robust variance-covariance matrix was calculated, which includes the variances (SEs) and covariances (how much the estimates correlate with each other):

```
## Model- & sandwich-based standard errors and p-values
## Click on the arrow for sandwich-based p-values.
## # A tibble: 2 × 6
##   term        estimate `Model-based s.e.` `Model-based p-value` `Sandwich s.e.`
##   <chr>          <dbl>              <dbl>                 <dbl>           <dbl>
## 1 (Intercept)    -5.54              3.07               8.08e- 2           2.48
## 2 emg             1.45              0.105              1.28e-14           0.119

```

```
##   `Sandwich p-value`
##                <dbl>
## 1       0.0419
## 2       0.0000000352
```

```
## # Indices of model performance
##
## AIC   |  AICc |   BIC | R2 (cond.) | R2 (marg.) |   ICC |  RMSE | Sigma
## -----------------------------------------------------------------------
## 228.8 | 230.2 | 234.9 |      0.923 |      0.691 | 0.751 | 2.888 | 3.822
## On average, predictions of measured torque were inaccurate by: 2.888269 % MVT
```

\##
\## Repeated measures correlation
\##
\## r
\## 0.9656414
\##
\## degrees of freedom
\## 16
\##
\## p-value
\## 8.767303e-11
\##
\## 95% confidence interval
\## 0.9082261 0.9873744

## Intramuscular coherence analysis: Area under the curve within the delta band model fitting and results for algorithms combined

Fixed effects were fitted using a linear mixed-effects model. Model-based standard errors and p-values are presented. Sandwich variance estimation-based standard errors and p-values were also produced; these do not assume normality of the data. A Wald statistic based on the sandwich variance estimate was also produced to test the effect of factor condition.

### Random-intercept model with no interaction

This model fits the factors decomp (i.e. decomposition algorithm), intensity, and condition with random intercepts and fixed slopes. We first compared fitting the model by REML vs. ML, and REML resulted in a better fit:

\## $statistics
\##	aic	bic bayes.factor
\## no_interaction_REML 1275.270 1298.497	33.299
\## no_interaction_ML	1282.281 1305.508	0.030
\##
\## $predicted_differences
\##	0%	25%	50%	75% 100%
\## 0.002 0.009 0.011 0.023 0.034

### Random-intercept model with interaction

This model fits the factors decomp, intensity, condition, and the intensity x condition interaction with random intercepts and fixed slopes. The fixed-effects model with an intensity:condition interaction resulted in a marginally better fit:

```
## $statistics
##                         aic      bic bayes.factor
## no_interaction_REML 1275.27 1298.497        0.493
## interaction_REML    1267.22 1297.083        2.027
##
## $predicted_differences
##    0%   25%   50%   75%  100%
## 0.000 0.013 1.076 1.088 1.106
```

#### Model summary

```
## Linear mixed model fit by REML. t-tests use Satterthwaite's method [
## lmerModLmerTest]
## Formula: outcome ~ decomp + intensity * condition + (1 | pid)
##    Data: d
##
## REML criterion at convergence: 1249.2
##
## Scaled residuals:
##     Min      1Q  Median      3Q     Max
## -2.8635 -0.7031  0.0085  0.5992  3.5055
##
## Random effects:
##  Groups   Name        Variance Std.Dev.
##  pid      (Intercept) 20.54    4.532
##  Residual             24.03    4.902
## Number of obs: 204, groups:  pid, 17
##
## Fixed effects:
##                        Estimate Std. Error        df t value Pr(>|t|)
## (Intercept)            23.43003    1.42575  36.68261  16.433  < 2e-16 ***
## decompMUedit           -6.93337    0.68644 181.00000 -10.100  < 2e-16 ***
## intensity2             -4.01640    1.18895 181.00000  -3.378 0.000894 ***
## condition2             -0.09448    1.18895 181.00000  -0.079 0.936748
## condition3             -0.33671    1.18895 181.00000  -0.283 0.777347
## intensity2:condition2   2.17297    1.68144 181.00000   1.292 0.197891
## intensity2:condition3   4.33297    1.68144 181.00000   2.577 0.010764 *
## ---
## Signif. codes:  0 '***' 0.001 '**' 0.01 '*' 0.05 '.' 0.1 ' ' 1
##

```

```
## Correlation of Fixed Effects:
##             (Intr) dcmpMU intns2 cndtn2 cndtn3 int2:2
## decompMUedt -0.241
## intensity2  -0.417  0.000
## condition2  -0.417  0.000  0.500
## condition3  -0.417  0.000  0.500  0.500
## intnsty2:c2  0.295  0.000 -0.707 -0.707 -0.354
## intnsty2:c3  0.295  0.000 -0.707 -0.354 -0.707  0.500
```

#### Model- & sandwich-based standard errors and p-values

```
## Model- & sandwich-based standard errors and p-values
## Click on the arrow for sandwich-based p-values.
## # A tibble: 7 × 6
##   term                  estimate `Model-based s.e.` `Model-based p-value`
##   <chr>                    <dbl>              <dbl>                 <dbl>
## 1 (Intercept)            23.4                 1.43               1.17e-38
## 2 decompMUedit           -6.93                0.686              1.47e-19
## 3 intensity2             -4.02                1.19               8.81e- 4
## 4 condition2             -0.0945              1.19               9.37e- 1
## 5 condition3             -0.337               1.19               7.77e- 1
## 6 intensity2:condition2   2.17                1.68               1.98e- 1
## 7 intensity2:condition3   4.33                1.68               1.07e- 2
##   `Sandwich s.e.` `Sandwich p-value`
##             <dbl>              <dbl>
## 1            1.54           6.24e-11
## 2            1.04           5.22e- 6
## 3            1.45           1.36e- 2
## 4            1.34           9.45e- 1
## 5            1.13           7.69e- 1
## 6            1.47           1.58e- 1
## 7            1.33           5.05e- 3
```

#### Interaction, intensity & condition p-values

The Wald statistics testing for an effect of the intensity:condition interaction term, for an effect of the intensity term and the intensity:condition interaction term, and for an effect of the condition term and the intensity:condition interaction term are:

```
##  test Fstat df_num df_denom   p_val sig
##   HTZ  5.09      2       15 0.02056   *
##  test Fstat df_num df_denom   p_val sig
##   HTZ  4.92      3       14 0.01535   *
##  test Fstat df_num df_denom   p_val sig
##   HTZ  4.64      4       13 0.01515   *
```

#### Post-hoc comparisons

Estimated marginal means (EMMs) for the interaction between intensity and condition for the random-intercept model, while explicitly using cluster-robust standard errors (which accounts for non-independent data and heteroskedasticity), are reported below. This approach was used to ensure that the confidence intervals and subsequent p-values for the EMMs are more accurate and reliable.

```
##  intensity condition emmean   SE   df lower.CL upper.CL
##  1         1           20.0 1.54 32.8     16.8     23.1
##  2         1           15.9 1.18 32.8     13.5     18.3
##  1         2           19.9 1.46 32.8     16.9     22.8
##  2         2           18.0 1.57 32.8     14.8     21.2
##  1         3           19.6 1.48 32.8     16.6     22.6
##  2         3           19.9 1.60 32.8     16.7     23.2
##
## Results are averaged over the levels of: decomp
## Degrees-of-freedom method: satterthwaite
## Confidence level used: 0.95
```

```
## condition = 1:
##  contrast                estimate   SE  df t.ratio p.value
##  intensity1 - intensity2    4.016 1.45 181   2.770  0.0062
##
## condition = 2:
##  contrast                estimate   SE  df t.ratio p.value
##  intensity1 - intensity2    1.843 1.65 181   1.117  0.2655
##
## condition = 3:
##  contrast                estimate   SE  df t.ratio p.value
##  intensity1 - intensity2   -0.317 1.76 181  -0.179  0.8578
##
## Results are averaged over the levels of: decomp
## Degrees-of-freedom method: satterthwaite
```

```
## intensity = 1:
##  contrast                estimate    SE  df t.ratio p.value
##  condition1 - condition2   0.0945 1.340 181   0.070  1.0000
##  condition1 - condition3   0.3367 1.130 181   0.298  1.0000
##  condition2 - condition3   0.2422 0.680 181   0.356  1.0000
##
## intensity = 2:
##  contrast                estimate    SE  df t.ratio p.value
##  condition1 - condition2  -2.0785 1.080 181  -1.928  0.0964
##  condition1 - condition3  -3.9963 0.853 181  -4.686  <.0001
##  condition2 - condition3  -1.9178 0.964 181  -1.989  0.0964
##
## Results are averaged over the levels of: decomp
## Degrees-of-freedom method: satterthwaite
## P value adjustment: holm method for 3 tests
```

### Random-slope-and-intercept model with interaction

This model fits the factors decomp, intensity, condition, and the intensity x condition interaction with random intercepts and random slopes. This random-slope-and-intercept model did not result in a better fit than the random-intercept model with an intensity:condition interaction:

\## $statistics
\##	aic	bic bayes.factor
\## interaction_REML	1267.220 1297.083	18885288
\## interaction_REML_randomSlope 1211.139 1330.591	0
\##
\## $predicted_differences
\##	0%	25%	50%	75%	100%
\## 0.012 0.972 2.242 3.615 11.799

## Intramuscular coherence analysis: Area under the curve within the delta band following the manual DEMUSE approach

Fixed effects were fitted using a linear mixed-effects model. Model-based standard errors and p-values are presented. Sandwich variance estimation-based standard errors and p-values were also produced; these do not assume normality of the data. A Wald statistic based on the sandwich variance estimate was also produced to test the effect of factor condition.

### Random-intercept model with no interaction

This model fits the factors decomp (i.e. decomposition algorithm), intensity, and condition with random intercepts and fixed slopes. We first compared fitting the model by REML vs. ML, and REML resulted in a better fit:

\## $statistics
\##	aic	bic bayes.factor
\## no_interaction_REML 637.329 653.079	48.694
\## no_interaction_ML	645.100 660.850	0.021
\##
\## $predicted_differences
\##	0%	25%	50%	75% 100%
\## 0.002 0.005 0.011 0.016 0.034

### Random-intercept model with interaction

This model fits the factors decomp, intensity, condition, and the intensity x condition interaction with random intercepts and fixed slopes. The fixed-effects model with an intensity:condition interaction resulted in a marginally better fit:

```
## $statistics
##                         aic     bic bayes.factor
## no_interaction_REML 637.329 653.079        1.170
## interaction_REML    632.393 653.393        0.855
##
## $predicted_differences
##    0%   25%   50%   75%  100%
## 0.396 0.403 0.579 0.979 0.986
```

#### Model summary

```
## Linear mixed model fit by REML. t-tests use Satterthwaite's method [
## lmerModLmerTest]
## Formula: outcome ~ intensity * condition + (1 | pid)
##    Data: d
##
## REML criterion at convergence: 616.4
##
## Scaled residuals:
##      Min       1Q   Median       3Q      Max
## -1.86648 -0.56157  0.00946  0.46862  2.62738
##
## Random effects:
##  Groups   Name        Variance Std.Dev.
##  pid      (Intercept) 24.44    4.944
##  Residual             21.37    4.623
## Number of obs: 102, groups:  pid, 17
##
## Fixed effects:
##                       Estimate Std. Error     df t value Pr(>|t|)
## (Intercept)             23.694      1.642 39.617  14.433   <2e-16 ***
## intensity2              -3.022      1.586 80.000  -1.906   0.0602 .
## condition2              -1.232      1.586 80.000  -0.777   0.4394
## condition3              -1.171      1.586 80.000  -0.739   0.4622
## intensity2:condition2    3.119      2.243 80.000   1.391   0.1681
## intensity2:condition3    2.763      2.243 80.000   1.232   0.2216
## ---
## Signif. codes:  0 '***' 0.001 '**' 0.01 '*' 0.05 '.' 0.1 ' ' 1
##
```

```
## Correlation of Fixed Effects:
##             (Intr) intns2 cndtn2 cndtn3 int2:2
## intensity2  -0.483
## condition2  -0.483  0.500
## condition3  -0.483  0.500  0.500
## intnsty2:c2  0.341 -0.707 -0.707 -0.354
## intnsty2:c3  0.341 -0.707 -0.354 -0.707  0.500
```

#### Model- & sandwich-based standard errors and p-values

```
## Model- & sandwich-based standard errors and p-values
## Click on the arrow for sandwich-based p-values.
## # A tibble: 6 × 6
##   term                  estimate `Model-based s.e.` `Model-based p-value`
##   <chr>                    <dbl>              <dbl>                 <dbl>
## 1 (Intercept)              23.7                1.64              1.41e-25
## 2 intensity2               -3.02               1.59              5.97e- 2
## 3 condition2               -1.23               1.59              4.39e- 1
## 4 condition3               -1.17               1.59              4.62e- 1
## 5 intensity2:condition2     3.12               2.24              1.68e- 1
## 6 intensity2:condition3     2.76               2.24              2.21e- 1
##   `Sandwich s.e.` `Sandwich p-value`
##             <dbl>              <dbl>
## 1            1.74           3.21e-10
## 2            1.61           7.88e- 2
## 3            1.61           4.54e- 1
## 4            1.30           3.82e- 1
## 5            1.80           1.02e- 1
## 6            1.58           1.00e- 1
```

#### Interaction, intensity & condition p-values

The Wald statistics testing for an effect of the intensity:condition interaction term, for an effect of the intensity term and the intensity:condition interaction term, and for an effect of the condition term and the intensity:condition interaction term are:

```
##  test Fstat df_num df_denom  p_val sig
##   HTZ  1.78      2       15 0.2028
##  test Fstat df_num df_denom  p_val sig
##   HTZ  1.62      3       14 0.2294
##  test Fstat df_num df_denom  p_val sig
##   HTZ 0.991      4       13 0.4465
```

#### Post-hoc comparisons

Estimated marginal means (EMMs) for the interaction between intensity and condition for the random-intercept model, while explicitly using cluster-robust standard errors (which accounts for non-independent data and heteroskedasticity), are reported below. This approach was used to ensure that the confidence intervals and subsequent p-values for the EMMs are more accurate and reliable.

```
##  intensity condition emmean   SE   df lower.CL upper.CL
##  1         1           23.7 1.74 39.6     20.2     27.2
##  2         1           20.7 1.38 39.6     17.9     23.5
##  1         2           22.5 1.23 39.6     20.0     25.0
##  2         2           22.6 1.92 39.6     18.7     26.4
##  1         3           22.5 1.55 39.6     19.4     25.7
##  2         3           22.3 1.90 39.6     18.4     26.1
##
## Degrees-of-freedom method: satterthwaite
## Confidence level used: 0.95
## condition = 1:
##  contrast                estimate   SE df t.ratio p.value
##  intensity1 - intensity2   3.0223 1.61 80   1.878  0.0641
##
## condition = 2:
##  contrast                estimate   SE df t.ratio p.value
##  intensity1 - intensity2  -0.0971 1.55 80  -0.063  0.9502
##
## condition = 3:
##  contrast                estimate   SE df t.ratio p.value
##  intensity1 - intensity2   0.2597 2.04 80   0.127  0.8989
##
## Degrees-of-freedom method: satterthwaite
```

\## intensity = 1:
\## contrast	estimate	SE df t.ratio p.value
\## condition1 - condition2	1.2322 1.610 80	0.767 1.0000
\## condition1 - condition3	1.1714 1.300 80	0.898 1.0000
\## condition2 - condition3 -0.0607 0.834 80 -0.073 1.0000
\##
\## intensity = 2:
\## contrast	estimate	SE df t.ratio p.value
\## condition1 - condition2 -1.8872 1.280 80 -1.473 0.4016
\## condition1 - condition3 -1.5912 1.050 80 -1.514 0.4016
\## condition2 - condition3	0.2960 1.200 80	0.246 0.8064
\##
\## Degrees-of-freedom method: satterthwaite
\## P value adjustment: holm method for 3 tests

## Intramuscular coherence analysis: Area under the curve within the delta band following the automatic MUedit approach

Fixed effects were fitted using a linear mixed-effects model. Model-based standard errors and p-values are presented. Sandwich variance estimation-based standard errors and p-values were also produced; these do not assume normality of the data. A Wald statistic based on the sandwich variance estimate was also produced to test the effect of factor condition.

### Random-intercept model with no interaction

This model fits the factors decomp (i.e. decomposition algorithm), intensity, and condition with random intercepts and fixed slopes. We first compared fitting the model by REML vs. ML, and REML resulted in a better fit:

\## $statistics
\##	aic	bic bayes.factor
\## no_interaction_REML 635.875 651.625	46.770
\## no_interaction_ML	643.566 659.315	0.021
\##
\## $predicted_differences
\##	0%	25%	50%	75% 100%
\## 0.003 0.005 0.009 0.018 0.033

### Random-intercept model with interaction

This model fits the factors decomp, intensity, condition, and the intensity x condition interaction with random intercepts and fixed slopes. The fixed-effects model with an intensity:condition interaction resulted in a marginally better fit:

```
## $statistics
##                         aic     bic bayes.factor
## no_interaction_REML 635.875 651.625        0.070
## interaction_REML    625.317 646.317       14.211
##
## $predicted_differences
##    0%   25%   50%   75%  100%
## 0.481 0.602 1.188 1.736 1.857
```

#### Model summary

```
## Linear mixed model fit by REML. t-tests use Satterthwaite's method [
## lmerModLmerTest]
## Formula: outcome ~ intensity * condition + (1 | pid)
##    Data: d
##
## REML criterion at convergence: 609.3
##
## Scaled residuals:
##     Min      1Q  Median      3Q     Max
## -2.4246 -0.5039  0.0490  0.4698  3.3150
##
## Random effects:
##  Groups   Name        Variance Std.Dev.
##  pid      (Intercept) 22.91    4.786
##  Residual             19.82    4.452
## Number of obs: 102, groups:  pid, 17
##
## Fixed effects:
##                       Estimate Std. Error     df t value Pr(>|t|)
## (Intercept)             16.232      1.585 39.392  10.238 1.17e-12 ***
## intensity2              -5.011      1.527 80.000  -3.281  0.00153 **
## condition2               1.043      1.527 80.000   0.683  0.49653
## condition3               0.498      1.527 80.000   0.326  0.74521
## intensity2:condition2    1.226      2.160 80.000   0.568  0.57169
## intensity2:condition3    5.903      2.160 80.000   2.733  0.00772 **
## ---
## Signif. codes:  0 '***' 0.001 '**' 0.01 '*' 0.05 '.' 0.1 ' ' 1
##
```

```
## Correlation of Fixed Effects:
##             (Intr) intns2 cndtn2 cndtn3 int2:2
## intensity2  -0.482
## condition2  -0.482  0.500
## condition3  -0.482  0.500  0.500
## intnsty2:c2  0.341 -0.707 -0.707 -0.354
## intnsty2:c3  0.341 -0.707 -0.354 -0.707  0.500
```

#### Model- & sandwich-based standard errors and p-values

```
## Model- & sandwich-based standard errors and p-values
## Click on the arrow for sandwich-based p-values.
## # A tibble: 6 × 6
##   term                  estimate `Model-based s.e.` `Model-based p-value`
##   <chr>                    <dbl>              <dbl>                 <dbl>
## 1 (Intercept)             16.2                 1.59              5.74e-17
## 2 intensity2              -5.01                1.53              1.45e- 3
## 3 condition2               1.04                1.53              4.96e- 1
## 4 condition3               0.498               1.53              7.45e- 1
## 5 intensity2:condition2    1.23                2.16              5.71e- 1
## 6 intensity2:condition3    5.90                2.16              7.49e- 3
##   `Sandwich s.e.` `Sandwich p-value`
##             <dbl>              <dbl>
## 1            1.70       0.0000000527
## 2            1.54       0.00499
## 3            1.27       0.424
## 4            1.18       0.678
## 5            1.51       0.427
## 6            1.29       0.000319
```

#### Interaction, intensity & condition p-values

The Wald statistics testing for an effect of the intensity:condition interaction term, for an effect of the intensity term and the intensity:condition interaction term, and for an effect of the condition term and the intensity:condition interaction term are:

```
##  test Fstat df_num df_denom    p_val sig
##   HTZ  10.3      2       15 0.001533  **
##  test Fstat df_num df_denom    p_val sig
##   HTZ  7.85      3       14 0.002587  **
##  test Fstat df_num df_denom     p_val sig
##   HTZ  12.6      4       13 0.0002096 ***
```

#### Post-hoc comparisons

Estimated marginal means (EMMs) for the interaction between intensity and condition for the random-intercept model, while explicitly using cluster-robust standard errors (which accounts for non-independent data and heteroskedasticity), are reported below. This approach was used to ensure that the confidence intervals and subsequent p-values for the EMMs are more accurate and reliable.

```
##  intensity condition emmean   SE   df lower.CL upper.CL
##  1         1           16.2 1.70 39.4    12.79     19.7
##  2         1           11.2 1.26 39.4     8.67     13.8
##  1         2           17.3 1.96 39.4    13.32     21.2
##  2         2           13.5 1.44 39.4    10.57     16.4
##  1         3           16.7 1.55 39.4    13.59     19.9
##  2         3           17.6 1.50 39.4    14.59     20.7
##
## Degrees-of-freedom method: satterthwaite
## Confidence level used: 0.95
## condition = 1:
##  contrast                estimate   SE df t.ratio p.value
##  intensity1 - intensity2    5.011 1.54 80   3.253  0.0017
##
## condition = 2:
##  contrast                estimate   SE df t.ratio p.value
##  intensity1 - intensity2    3.784 1.90 80   1.987  0.0503
##
## condition = 3:
##  contrast                estimate   SE df t.ratio p.value
##  intensity1 - intensity2   -0.893 1.72 80  -0.520  0.6042
##
## Degrees-of-freedom method: satterthwaite
## intensity = 1:
##  contrast                estimate    SE df t.ratio p.value
##  condition1 - condition2   -1.043 1.270 80  -0.821  1.0000
##  condition1 - condition3   -0.498 1.180 80  -0.423  1.0000
##  condition2 - condition3    0.545 1.090 80   0.502  1.0000
##
## intensity = 2:
##  contrast                estimate    SE df t.ratio p.value
##  condition1 - condition2   -2.270 1.030 80  -2.211  0.0299
##  condition1 - condition3   -6.401 0.869 80  -7.362  <.0001
##  condition2 - condition3   -4.132 1.120 80  -3.679  0.0008
##
## Degrees-of-freedom method: satterthwaite
## P value adjustment: holm method for 3 tests
```

## References

Avrillon S, Hug F, Baker SN, Gibbs C & Farina D (2024). Tutorial on MUedit: An open-source software for identifying and analysing the discharge timing of motor units from electromyographic signals. *J Electromyogr Kinesiol* **77,** 102886.

Del Vecchio A, Holobar A, Falla D, Felici F, Enoka RM & Farina D (2020). Tutorial: Analysis of motor unit discharge characteristics from high-density surface EMG signals. *J Electromyogr Kinesiol* **53,** 102426.

Holobar A, Minetto MA, Botter A, Negro F & Farina D (2010). Experimental analysis of accuracy in the identification of motor unit spike trains from high-density surface EMG. *IEEE Trans Neural Syst Rehabil Eng* **18,** 221–229.

Holobar A & Zazula D (2007*a*). Gradient convolution kernel compensation applied to surface electromyograms. In *Independent Component Analysis and Signal Separation*, ed. Davies ME, James CJ, Abdallah SA & Plumbley MD, Lecture Notes in Computer Science, pp. 617–624. Springer Berlin Heidelberg, Berlin, Heidelberg. Available at: http://link.springer.com/10.1007/978-3-540-74494-8_77 [Accessed August 14, 2025].

Holobar A & Zazula D (2007*b*). Multichannel blind source separation using convolution kernel compensation. *IEEE Trans Signal Process* **55,** 4487–4496.

Hug F, Avrillon S, Del Vecchio A, Casolo A, Ibanez J, Nuccio S, Rossato J, Holobar A & Farina D (2021). Analysis of motor unit spike trains estimated from high-density surface electromyography is highly reliable across operators. *J Electromyogr Kinesiol* **58,** 102548.

Murks N, Škarabot J, Kramberger M, Divjak M, Sedej G, Valenčič T, Connelly CD, Thomason H & Holobar A (2025). The efficiency of manual editing of high-density surface electromyogram decomposition depends on the recorded muscle and contraction level but less on the operator’s experience. *IEEE Trans Neural Syst Rehabil Eng* **33,** 3367–3376.

Wade L, Needham L, Evans M, McGuigan P, Colyer S, Cosker D & Bilzon J (2023). Examination of 2D frontal and sagittal markerless motion capture: Implications for markerless applications ed. Gu Y. *PLoS ONE* **18,** e0293917.
